# Comparative Effects of Therapeutic Exercise and Manual Therapy Techniques on Self-Reported Disability in Chronic Non-Specific Low Back Pain: A Network Meta-Analysis

**DOI:** 10.3390/jcm15124809

**Published:** 2026-06-21

**Authors:** Miguel Robles-García, Juan Luis Sánchez González, José Luis Sánchez-Sánchez, Laura Calderón-Díez, Miguel Santos Del Rey, Javier Martín-Vallejo

**Affiliations:** 1 Departamento de Anatomia e Histología, Facultad de Medicina, Campus Miguel de Unamuno, Universidad de Salamanca, s/n, 37007 Salamanca, Spain; mroblesgarcia@usal.es (M.R.-G.); lauca@usal.es (L.C.-D.); msdr@usal.es (M.S.D.R.); 2 Departamento de Medicina, Universidad de Salamanca, Instituto de Investigación Biomédica de Salamanca (IBSAL), Campus Miguel de Unamuno s/n, 37007 Salamanca, Spain; 3 Departamento de Enfermería y Fisioterapia, Universidad de Salamanca, Instituto de Investigación Biomédica de Salamanca (IBSAL), Campus Miguel de Unamuno s/n, 37007 Salamanca, Spain; jlsanchez@usal.es; 4 Departamento de Estadística, Universidad de Salamanca, Instituto de Investigación Biomédica de Salamanca (IBSAL), Campus Miguel de Unamuno s/n, 37007 Salamanca, Spain; jmv@usal.es

**Keywords:** chronic low back pain, non-specific low back pain, therapeutic exercise, manual therapy, disability, network meta-analysis, Pilates, stabilization exercise, soft tissue manipulation

## Abstract

**Background/Objectives:** Chronic non-specific low back pain is a leading cause of disability. Although therapeutic exercise and manual therapy are commonly recommended, their relative effects are often interpreted using broad therapeutic categories. This network meta-analysis aimed to compare the relative effectiveness of specific therapeutic exercise and manual therapy techniques on post-treatment self-reported disability in adults with chronic non-specific low back pain. **Methods:** A systematic review and frequentist random-effects network meta-analysis were conducted according to Cochrane recommendations and PRISMA-NMA guidance. The protocol was registered in PROSPERO (CRD42022331411). Randomized controlled trials including adults aged 18–65 years with chronic non-specific low back pain were searched in CENTRAL, PubMed, PEDro, Google Scholar, and SciELO up to 31 March 2026. Disability was assessed using the Roland–Morris Disability Questionnaire or Oswestry Disability Index. Effects were synthesized as standardized mean differences. Risk of bias was assessed with RoB 2, and confidence in network estimates was evaluated using CINeMA. **Results:** Forty-five studies were included. Compared with control/placebo, the largest favorable estimates were observed for equipment-based Pilates, stabilization with motor control, stabilization exercise, soft tissue manipulation, and Pilates Mat. Equipment-based Pilates showed the largest favorable estimate with moderate-confidence evidence, and soft tissue manipulation also showed moderate-confidence evidence. However, heterogeneity was substantial, and confidence in most favorable exercise estimates was low. **Conclusions:** Specific exercise and manual therapy techniques may reduce post-treatment disability in adults with chronic non-specific low back pain. Equipment-based Pilates and soft tissue manipulation showed favorable signals supported by moderate-confidence evidence. However, the findings do not support a definitive hierarchy of efficacy or categorical superiority of therapeutic exercise over manual therapy.

## 1. Introduction

Low back pain is the leading cause of disability worldwide and one of the conditions with the greatest potential need for rehabilitation [1]. Its burden shows an increasing trajectory. In 2020, low back pain affected 619 million people worldwide, and this figure is projected to rise to 843 million by 2050 [2]. Approximately 90% of cases are classified as non-specific [3]. Low back pain is considered chronic when symptoms persist for more than 12 weeks from onset [4]. Beyond its clinical consequences, non-specific chronic low back pain (NSCLBP) generates a substantial economic, healthcare, and social burden in both high-income and low- and middle-income countries [5,6].

Because NSCLBP has a particularly relevant impact on functional limitation, self-reported disability is a central outcome for assessing the clinical effects of interventions. In this context, current clinical guidelines recommend non-surgical, person-centered care, with therapeutic exercise as one of its main pillars. Certain manual therapy techniques are also considered, especially when they are integrated into multimodal strategies adapted to patient characteristics and preferences [4,7,8,9,10,11,12].

Randomized controlled trials (RCTs) evaluating different forms of therapeutic exercise and manual therapy techniques in patients with NSCLBP have increased substantially in recent years. However, this body of evidence remains difficult to interpret due to variability in the interventions, heterogeneity in dosage and supervision, diversity of comparators, and the use of different instruments to assess disability. Pairwise meta-analysis can quantitatively synthesize direct comparisons, but it is limited when multiple potentially relevant interventions have not been compared directly with each other. In this context, network meta-analysis (NMA) provides a methodological framework for combining direct and indirect evidence and estimating the relative effectiveness of multiple interventions within the same analysis [13,14].

Several previous NMAs have examined the comparative efficacy of different therapeutic exercise modalities in patients with chronic low back pain. Pilates, strengthening exercises, core-based exercises, and motor control or stabilization programs have repeatedly been identified among the interventions with the most favorable relative performance for pain and disability [15,16,17,18,19,20]. However, these NMAs have mainly focused on comparisons between exercise modalities. Manual therapy has usually been considered as a broad comparator, an undifferentiated conservative treatment, or a component of more heterogeneous categories. This approach limits the possibility of drawing specific clinical inferences about concrete manual techniques, such as spinal manipulation or soft tissue manipulation. It also makes it more difficult to compare specific exercise and manual therapy modalities in a clinically meaningful way.

Consistent with this issue, the Cochrane review on exercise for chronic low back pain compared different exercise modalities not only with control but also with several non-invasive physiotherapy treatments. It was observed that exercise was probably more effective than advice or education alone, and also more effective than electrotherapy. However, no clear differences were identified when exercise was compared with manual therapy treatments [21]. As these comparisons were based on broad therapeutic categories, an important clinical question remains unresolved: whether a more specific classification of therapeutic exercise and manual therapy techniques could allow a more precise interpretation of their relative efficacy on self-reported disability.

Broad therapeutic labels such as “exercise therapy” or “manual therapy” may group interventions that differ substantially in therapeutic rationale, delivery requirements, progression, supervision and expected clinical use. For example, equipment-based Pilates and Mat Pilates share general principles but differ in external support, load progression and sensorimotor feedback; similarly, stabilization exercise with explicit motor-control retraining is not equivalent to non-specific trunk stabilization, and soft tissue manipulation should not be interpreted as clinically interchangeable with spinal manipulation. This lack of specificity may limit the usefulness of previous evidence syntheses for individualized rehabilitation decisions. Therefore, a more specific classification of therapeutic exercise and manual therapy techniques may help clinicians interpret which interventions show the most favorable disability outcomes, while also identifying where uncertainty remains. Moreover, treatment effects may also depend on dose, session duration, supervision, progression strategy, adherence and home practice, which may contribute to heterogeneity and limit the direct clinical transferability of broad therapeutic categories.

The protocol for the present study already identified the need to compare selected therapeutic exercise and manual therapy techniques using NMA. This approach was intended to reduce the heterogeneity produced by grouping clinically different interventions within the same category [13,14]. Since protocol registration, González-Gómez et al. reported a small advantage of therapeutic exercise over manual therapy for long-term disability. Nevertheless, the certainty of the evidence was very low, and the authors could not establish conclusive differences between the two modalities when analyzed as isolated treatments [22]. Because their study used pairwise meta-analysis, it could not simultaneously explore multiple specific modalities that had not been directly compared with each other [13,14,22]. Therefore, the objective of the present NMA was to compare the relative efficacy of specific therapeutic exercise and manual therapy techniques on self-reported disability in adults with NSCLBP at the end of the interventions. To address this objective, we applied a nodal classification based on the categories prespecified in the protocol and refined it using clinical and methodological criteria. This was done to improve the reproducibility of the classification and the clinical interpretability of the network.

## 2. Materials and Methods

This NMA was conducted following Cochrane methodological recommendations. It was reported according to the PRISMA extension statement for systematic reviews incorporating network meta-analyses of healthcare interventions: PRISMA-NMA [23]. The protocol was registered in PROSPERO on 20 May 2022 (registration ID: CRD42022331411).

### 2.1. Deviations from the Protocol

The present manuscript evaluates the outcome of self-reported disability at the end of the intervention. The pain outcome was not analyzed or interpreted in this manuscript.

The intervention nodes were defined according to the protocol. During the classification process and before quantitative analysis, operational refinements were introduced to improve reproducibility and clinical interpretability. Specifically, classical stabilization exercises (ST) were separated from stabilization exercises with an explicit motor control component (STMC). Mat Pilates programs (Pilates MAT) were also separated from programs in which apparatus use was a structural component of the intervention (Pilates APP). In addition, some active comparators could not be clearly assigned to the main nodes or to the control/placebo node. These were therefore included as accessory connectivity nodes: general exercise (GE), back school (BS), stretching (STRET), and usual care (UC).

Risk of bias was assessed using the Cochrane RoB 2 tool. The PEDro <6 exclusion criterion was prespecified in the registered protocol and was applied before quantitative synthesis as a minimum methodological adequacy filter to reduce the likelihood that trials with very limited methodological reporting or conduct contributed unstable or poorly interpretable data to the disability network. However, excluding trials on the basis of a summary methodological score may introduce quality-related selection bias and may obscure domain-specific sources of bias. This threshold was not used as a substitute for domain-level risk-of-bias assessment, nor was it used to weight studies, rank interventions, or determine the certainty of the evidence. Because PEDro summary scores and RoB 2 assess overlapping but non-equivalent constructs, the implications of this eligibility criterion are acknowledged in the limitations.

Although the protocol specified a fixed-effect model, the analysis was performed using a random-effects model. This decision was made after data extraction and before any quantitative network analysis, based on the clinical and methodological variability identified across eligible trials, including differences in intervention content, dose, duration, supervision, comparator characteristics and disability instruments.

The protocol was refined to assess the confidence in the network estimates for disability using CINeMA, a GRADE-based framework specifically developed for network meta-analysis. This addition was made to improve the interpretability and transparency of the network estimates. The assessment was restricted to the post-treatment disability outcome, which was the outcome addressed in the present manuscript and did not modify the eligibility criteria, intervention nodes, statistical model, or network effect estimates.

A detailed comparison between the registered PROSPERO protocol and the final review methods is provided in Supplementary Table S9.

### 2.2. Search Strategy

Two independent reviewers (FJMV and MRG) conducted a systematic search in CENTRAL, PubMed, PEDro, Google Scholar, and SciELO databases, up to 31 March 2026. The search aimed to identify randomized controlled trials (RCTs) assessing the effectiveness of manual therapy and therapeutic exercise techniques for reducing disability in people with NSCLBP.

The search strategy combined terms and Boolean operators. The specific details of the search strategies used in each database are provided in the Supplementaries Table S1a,b.

No language restrictions were applied. The Rayyan tool was used to identify duplicates and organize references. Disagreements were resolved by consensus or by a third independent reviewer (MSDR).

### 2.3. Eligibility Criteria

The inclusion criteria were defined according to the PICOS framework: participants, interventions, comparators, outcomes, and study design.

Participants: We included RCTs conducted in adults aged 18 to 65 years with chronic non-specific low back pain, defined as low back pain lasting 12 weeks or longer [4].

Type of intervention: We included trials in which the main intervention corresponded to one of the therapeutic exercise or manual therapy techniques prespecified in the protocol: progressive strengthening/resistance exercise (STR), stabilization exercises (ST), Pilates, McKenzie method (MDT), spinal manipulation (SM), soft tissue manipulation (STM), or dry needling (DN). Interventions were considered regardless of their frequency, intensity, or duration.

Comparator: The main comparator was pure control/placebo. This was defined as minimal intervention, waiting list, placebo, or isolated minimal education without a structured therapeutic exercise or manual therapy program.

Outcomes: The outcome was self-reported disability measured with the Roland–Morris Disability Questionnaire (RMDQ) or the Oswestry Disability Index (ODI). Both instruments are widely validated for assessing disability related to low back pain [24,25].

### 2.4. Study Design: RCTs

Studies were excluded if they specifically recruited participants with low back pain of identifiable origin, including diagnosed diskogenic or disk-related low back pain, radiculopathy, nerve-root involvement, post-surgical low back pain, recurrent low back pain, or other specific spinal pathology. Imaging-based exclusion of disc-related degenerative findings was not required when trials classified participants as having chronic non-specific low back pain. Studies were also excluded when follow-up was shorter than four weeks, when the PEDro score was <6, or when the intervention could not be assigned to a defined node. Additive designs were accepted only when both groups received an equivalent background co-intervention. This ensured that the differential effect could be attributed to the specific intervention under evaluation or to its placebo. Studies with unbalanced co-interventions or non-divisible multimodal comparators were excluded.

### 2.5. Classification of Interventions

Intervention nodes were established according to the protocol. During the classification process, and before quantitative analysis, the divisions described in the deviations section were applied. The final nodes were defined as follows:

Exercise-related interventions: Stabilization Exercises (ST), Stabilization Exercises With Motor Control (STMC), Strengthening Exercises (STR), Pilates Mat (PMAT), Equipment-based Pilates (PAPP), McKenzie technique (MDT).

Manual therapy-related interventions: Lumbar Spinal Manipulation (SM), Soft Tissue Manipulation (STM), Dry Needling (DN).

Control intervention: Control/Placebo (CTRL).

Accessory comparators: Back School (BS), Stretching (STRET), Usual Care (UC), General Exercise (GE).

The complete operational definitions for all intervention nodes are provided in the Supplementary Material (Table S2). Briefly, soft tissue manipulation was operationalised as physiotherapy techniques involving direct manual treatment of muscular or fascial tissues, including massage, myofascial techniques, or other soft tissue procedures.

Intervention-node classification was performed independently by two reviewers (FJMV and MRG), using the protocol-defined categories and the operational definitions reported in Supplementary Table S2. Disagreements were resolved by consensus; when consensus could not be reached, a third independent reviewer (MSDR) adjudicated the final node assignment.

### 2.6. Data Extraction

Two reviewers (FJMV and MRG) independently extracted the following data: author, country, year of publication, randomisation method, type of blinding, population characteristics, age, percentage of women, body mass index, sample size, intervention, intervention duration, losses to follow-up, post-treatment outcomes, and disability scale used. When discrepancies were found in the extracted outcomes, a third reviewer (MSDR) assessed the corresponding studies.

For disability outcomes, baseline and post-treatment values were extracted from the RMDQ and ODI scales. When both instruments were available for the same sample, RMDQ values were selected because of their superior construct validity [26].

### 2.7. Risk-of-Bias Assessment

Two reviewers (FJMV and MRG) independently assessed the risk of bias of the RCTs included in the analysis using the Cochrane Risk of Bias 2 (RoB 2) tool. Disagreements were resolved by consensus or by a third independent reviewer (MSDR).

### 2.8. Data Analysis

The analysis was conducted using a random-effects model. The network analysis was carried out using the frequentist perspective. The unbiased estimator of the standardized mean difference was used as the effect size because studies included different disability instruments and scales. Effect sizes were interpreted according to Cohen’s criteria, where values below 0.20 were classified as small, those between 0.20 and 0.80 as moderate, and values exceeding 0.80 as large. Transitivity was explored descriptively by tabulating available potential effect modifiers across direct comparisons. Available quantitative variables included baseline disability, disability instrument, treatment duration, age, sex distribution, body mass index, PEDro score and RoB 2 overall judgment. These variables were not incorporated as covariates in the network meta-analysis model, and no formal subgroup, meta-regression or adjusted network analyses were performed. Intervention-delivery variables, including frequency, supervision, progression, protocol fidelity/adherence and home practice, were extracted separately because they were heterogeneously described across trials and could not be consistently summarized quantitatively. The Q test and I^2^ were calculated to measure heterogeneity within designs (Q_W_) and inconsistency between (Q_I_) designs. Because Q_I_ is a global measure of inconsistency that cannot identify specific sources of discrepancy and may be inflated by substantial heterogeneity [27], the overall Q-test for the design-by-treatment interaction model was also computed. This model accounts for two distinct sources of discrepancy within the network. The first is loop inconsistency, which arises when direct evidence for a given comparison conflicts with the indirect evidence derived from closed treatment loops. The second is design inconsistency, which pertains to differences in effect estimates across studies evaluating different sets of treatments [28]. Inconsistency was also explored by aggregate mean values of the design and quality variables recorded, and by comparing the direct and indirect estimates of the different comparisons. The mean path length, minimal Parallelism and Net Heat Plot [29] procedures were used to analyze the validity of the results. The first coefficients indicate the consistency of the comparisons, and the heatplot indicates by the size of the squares and the background color how important the estimate of the comparison between a treatment in the rest of the comparisons and the inconsistency of the designs attributable to the rest of the designs, respectively. The p-score [30] was used as a procedure to rank the treatments involved according to their effect. Publication bias was explored using the Comparison-adjusted funnel plot [31]. Confidence intervals were calculated at 95% confidence, and a significance level of 5% was set. The netmeta (Netmeta 3.2.0) and dmetar libraries (dmetar 0.1.0) of the R statistics program were used to perform the analyses.

### 2.9. Certainty of the Evidence

Confidence in the network estimates for post-treatment disability was assessed using the CINeMA framework, a GRADE-based approach specifically developed for network meta-analysis [32]. The assessment considered six domains: within-study bias, reporting bias, indirectness, imprecision, heterogeneity, and incoherence.

Because disability was synthesized using standardized mean differences across different instruments, no single instrument-specific minimal clinically important difference could be applied consistently across the network. Therefore, a ±0.20 SMD decision threshold was defined before final CINeMA confidence adjudication and applied uniformly to imprecision, heterogeneity, and incoherence judgments. This value was selected as a conservative boundary for a small standardized effect, consistent with the conventional interpretation of Cohen-type standardized mean differences, and was used only as a methodological decision aid for judging whether uncertainty crossed a potentially relevant effect boundary. It should not be interpreted as an ODI- or RMDQ-specific minimal clinically important difference, and the threshold-dependent nature of the CINeMA ratings is acknowledged in the limitations [32,33].

Automatic CINeMA domain-level judgments were reviewed against additional network diagnostics defined before final confidence adjudication, including prediction intervals, contribution matrix, direct–indirect comparisons, design-level sensitivity analyses, minimal parallelism, mean path length, and the net heat plot. Manual adjudication was conservative and downgrade-only (manual adjudication fully documented in the Supplementary Material Text S1). Final confidence ratings were classified as high, moderate, low, or very low and reported in a CINeMA-based Summary of Findings table.

## 3. Results

**Figure 1:**
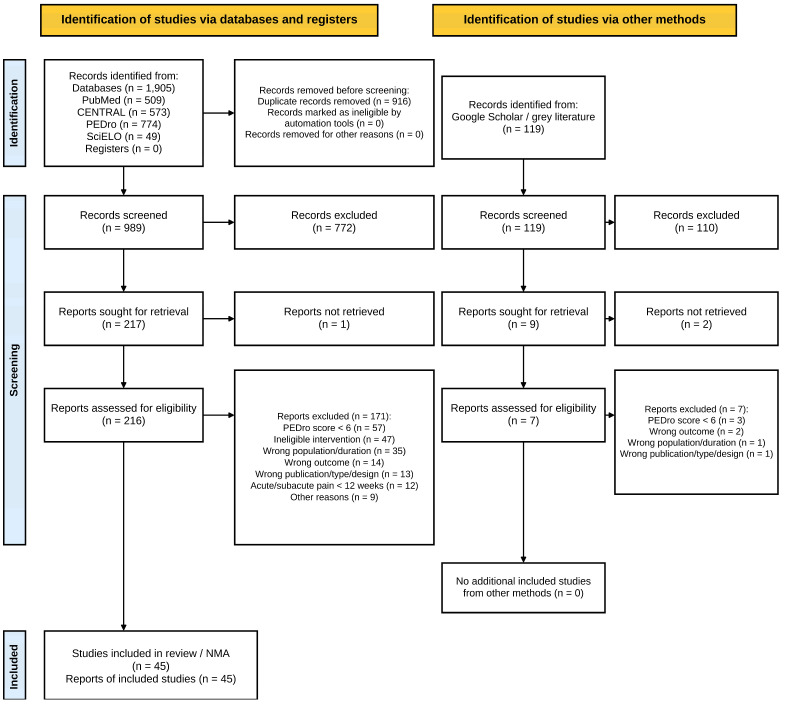
PRISMA flow diagram for the systematic review and network meta-analysis. Note. Adapted from PRISMA 2020 and PRISMA NMA.

Up to 31 March 2026, a total of 2024 records were identified: 1905 through database searches and 119 through other methods. 45 studies were included in the NMA [34,35,36,37,38,39,40,41,42,43,44,45,46,47,48,49,50,51,52,53,54,55,56,57,58,59,60,61,62,63,64,65,66,67,68,69,70,71,72,73,74,75,76,77,78]. The flow diagram is shown in Figure 1. Because no studies met the eligibility criteria for the dry needling intervention group, this node was removed from the analysis. Excluded studies and reasons for exclusion are reported in Supplementary Table S3. According to the PRISMA flow diagram, 57 full-text records were excluded with a PEDro score <6 as the primary reason recorded for PRISMA reporting. This category should be interpreted as the primary recorded reason rather than as an exclusive reason for exclusion, because some records could also have met other exclusion criteria.

### 3.1. Study Characteristics

The characteristics of the included studies are provided in the Supplementary Table S4a, together with the distribution of intervention nodes in the analytical network, Supplementary Table S4b, and the detailed description of the interventions, Supplementary Table S5.

### 3.2. Risk-of-Bias Assessment

**Figure 2:**
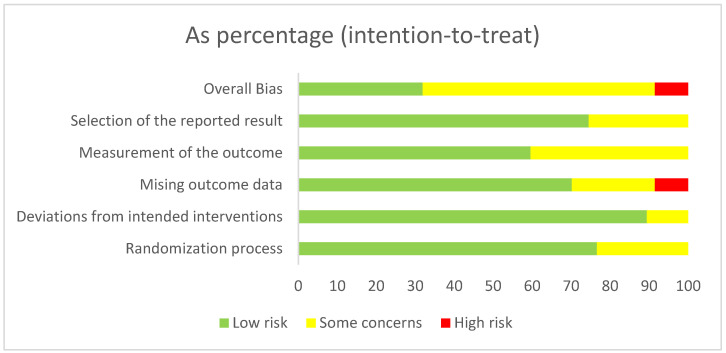
Risk of bias assessment across domains using the RoB 2 tool (intention-to-treat analysis). Note. Percentages indicate the distribution of studies across risk-of-bias judgments (low risk, some concerns, high risk) for each domain of the RoB 2 tool, based on intention-to-treat analyses.

A total of 48 comparisons from 45 studies were assessed using the Cochrane Risk of Bias 2 (RoB 2) tool. 15 comparisons (31.3%) were judged as low risk of bias, 29 (60.4%) as having some concerns, and 4 (8.3%) as high risk of bias (Figure 2). The risk-of-bias assessment of included intervention comparisons for disability outcome is presented in the Supplementary Figure S1.

Study characteristics and potential effect modifiers across direct comparisons, including sample sizes, sociodemographic data, baseline disability and PEDro quality scores, are detailed in Supplementary Table S6. The mean treatment duration was 6.5 weeks (range: 4–10). Mean age was balanced between groups (42.74 vs. 42.86 years), although participants in the STRET vs. CTRL and STMC vs. STR designs were younger on average. Most study designs favored a higher female-to-male ratio, except for four specific comparisons (STR vs. CTRL, MDT vs. UC, STMC vs. ST, and STMC vs. STR). Specific gender imbalances were identified in the MDT vs. UC design. Furthermore, lower PEDro scale scores within the eligible range (scores of 6–7) were observed for the STMC vs. ST, STR vs. GE, PILATES MAT vs. GE, ST vs. CTRL, and STMC vs. STR comparisons. Intervention-delivery variables, including frequency, supervision, progression, protocol fidelity/adherence and home practice, are reported in Supplementary Table S8. These variables were used for descriptive characterization of the evidence base and were not included as adjustment variables in the network model.

**Figure 3:**
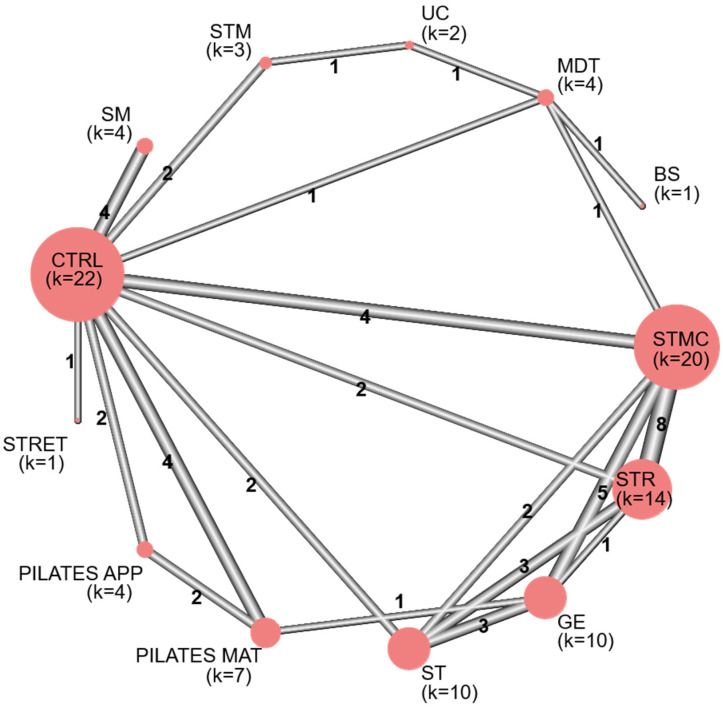
Network graph. Note. The diameter of the nodes is proportional to the number of studies (k) assigned to the intervention and the thickness of the line to the number of studies of the direct comparison.

The value of the heterogeneity test Q_T_ is 576.14 (*p* < 0.001 and I2 = 93.8%). The test of heterogeneity within designs was significant (Q_W_ = 576.14; df = 36; *p* < 0.001), and the Q_I_ test for inconsistency was statistically significant, indicating relevant between-design variability (Q_I_ = 63.01; df = 15; *p* < 0.001). However, the global inconsistency Q-test for the design-by-treatment interaction model was non-significant (Q = 3.74; df = 15, *p* = 0.998). This discrepancy arises because the Q_I_ test measures global inconsistency, which can be confounded by high between-study heterogeneity. Furthermore, it evaluates general inconsistency without partitioning the specific discrepancies stemming from the interaction between treatment effects and study designs, which are explicitly accounted for in the Q-test of the design-by-treatment interaction model. The network graph appears in Figure 3, where comparisons between the most common interventions can be visualized.

**Figure 4:**
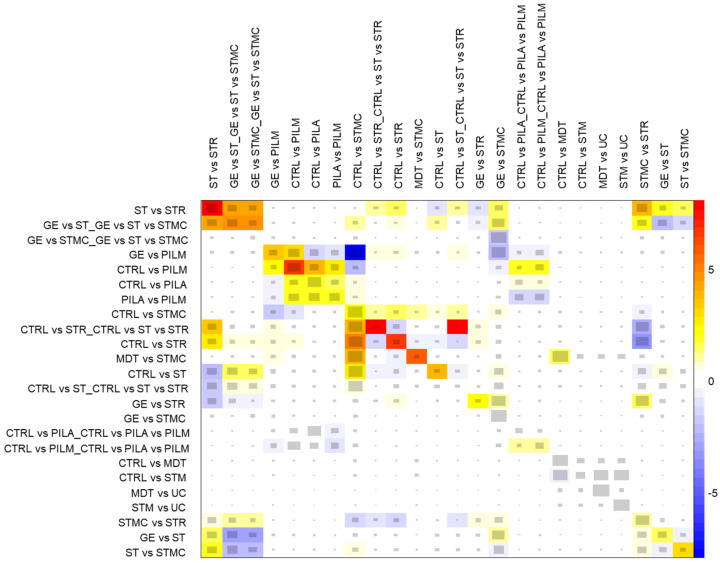
The heat map of the network of designs included in the network meta-analysis. Note. Heatmap of the design-by-treatment interaction model displaying the contribution matrix and standardized discrepancies. The size of the inner gray squares represents the leverage (statistical influence) of the treatment design on the horizontal axis on the network estimate on the vertical axis, with larger squares indicating greater contribution. The background color gradient maps the standardized residuals (Z-scores) of the discrepancies, centered at zero (white), which represents perfect consistency. Values deviating from zero—both warm colors (positive residuals) and cool colors (negative residuals)—indicate the magnitude and direction of network inconsistency. The intense coloration along the main diagonal highlights the specific designs that act as the primary drivers of the overall network inconsistency. PILM = Pilates Mat; PILA = Pilates Apparatus.

The sensitivity analysis, conducted by systematically excluding individual treatment designs, revealed that ST vs. STR, Pilates Mat vs. CTRL, STMC vs. CTRL, and ST vs. STR vs. CTRL were identified as localized sources of network inconsistency. Specifically, the isolation and removal of each of these designs yielded a substantial decrease of more than 7 points in the global Q-test score. To further contextualize these localized instabilities within the broader network architecture, Supplementary Figure S2 illustrates the contribution of direct evidence alongside coefficients for minimal parallelism and mean path length. Notably, several comparisons are characterized by high mean path lengths and low minimal parallelism; these architectural patterns reinforce the primary role of indirect evidence and highlight a limited redundancy within those specific network nodes. The Net Heat Plot (Figure 4) confirms these localized discrepancies; it shows that while the ST vs. STR design itself exhibits high internal consistency, it contributed to the inconsistency observed in the multi-arm designs in which it is embedded. Additionally, the Pilates Mat vs. CTRL design displays a high level of internal inconsistency. Ultimately, these combined diagnostic findings supported a conservative interpretation of the corresponding network estimates and supported the conservative adjudication of CINeMA confidence ratings where appropriate.

Supplementary Table S7 presents direct, indirect, and network estimates. No statistically significant differences were observed between direct and indirect estimates; however, the largest discrepancies were noted in the comparisons of Pilates Mat vs. GE, Pilates Mat vs. CTRL, ST vs. CTRL.

**Figure 5:**
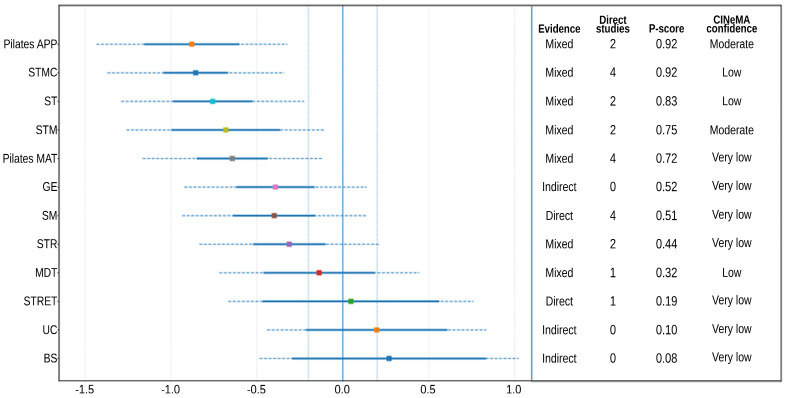
Network estimates versus control with P-score and CINeMA confidence for post-treatment disability. Note. Negative standardized mean difference values favor the intervention over control. Solid horizontal lines represent 95% confidence intervals, whereas dashed horizontal lines represent 95% prediction intervals. The vertical line at 0 indicates no effect, and the dotted vertical lines at ±0.20 indicate the SMD decision threshold used for CINeMA judgments of imprecision, heterogeneity and incoherence. This threshold should not be interpreted as an ODI- or RMDQ-specific minimal clinically important difference. Interventions are ordered according to P-score values; however, P-scores should be interpreted as descriptive ranking metrics only and not as a stand-alone hierarchy of treatment efficacy. Evidence type, number of direct studies and final CINeMA confidence ratings are displayed alongside each estimate to support interpretation according to effect size, uncertainty, heterogeneity, directness of evidence and confidence in the network estimate.

Compared with CTRL, the largest favorable point estimates for post-treatment disability were observed for Pilates APP (SMD = −0.879, 95% CI −1.156 to −0.601; 95% PrI −1.434 to −0.323; P-score = 0.92; moderate confidence), STMC (SMD = −0.856, 95% CI −1.044 to −0.667; 95% PrI −1.370 to −0.342; P-score = 0.92; low confidence), ST (SMD = −0.757, 95% CI −0.988 to −0.527; 95% PrI −1.290 to −0.225; P-score = 0.83; low confidence), STM (SMD = −0.681, 95% CI −0.998 to −0.365; 95% PrI −1.259 to −0.104; P-score = 0.75; moderate confidence), and Pilates MAT (SMD = −0.643, 95% CI −0.851 to −0.436; 95% PrI −1.165 to −0.121; P-score = 0.72; very low confidence). GE, SM, STR, MDT, STRET, UC, and BS showed smaller point estimates, wider uncertainty, and/or lower confidence versus CTRL. Negative SMD values favor the intervention. For clinical interpretation, the largest favorable estimates versus control ranged from approximately −0.64 to −0.88 SMD. Based on conventional Cohen-type benchmarks for standardized effects, these values correspond to moderate-to-large standardized effects [33]. However, because disability was synthesized using SMDs across ODI and RMDQ, these values cannot be translated into a single instrument-specific minimal clinically important difference. Therefore, clinical interpretation was based on the joint consideration of effect size, 95% confidence intervals, 95% prediction intervals, P-score values and CINeMA confidence ratings, rather than on statistical significance or ranking position alone, as summarized in Table 1 and Figure 5.

Moderate-confidence evidence supported Pilates APP and STM, whereas low-confidence evidence supported STMC and ST. Pilates MAT and most remaining comparisons were supported by very-low-confidence evidence. Thus, confidence ratings did not always parallel ranking values, and interventions with similar P-score positions should not be interpreted as having equivalent credibility.

The funnel plot reveals an asymmetrical distribution, with a lack of studies in the lower-left quadrant and several outliers on the right, potentially indicating large effect sizes in small-sample studies; see Supplementary Figure S3. Although Egger’s test was non-significant, the association between large standard errors and large effect sizes may suggest a potential risk of publication bias. However, it is worth noting that this pattern is driven by only a small subset of the included studies, indicating that this potential asymmetry is localized rather than widespread across the network. Reporting bias was therefore judged as undetected/no downgrade in CINeMA.

## 4. Discussion

This NMA was designed to compare the relative efficacy of specific therapeutic exercise and manual therapy techniques on self-reported disability in adults with non-specific chronic low back pain at the end of the intervention. Pilates APP, STMC, ST, STM, and Pilates MAT showed the most favorable signals compared with control. However, the high heterogeneity of the network and the limited certainty of the evidence require cautious interpretation. These findings should be viewed as signals of potential benefit rather than as a definitive hierarchy of clinical efficacy. Although the most favorable point estimates were of moderate-to-large standardized magnitude, their clinical interpretation should remain cautious. The use of SMDs allowed ODI and RMDQ outcomes to be analyzed within the same network, but it also prevents a direct and uniform translation of the results into scale-specific clinically important changes. Accordingly, clinical interpretation should consider effect size, confidence intervals, prediction intervals, risk of bias, heterogeneity, indirectness, P-score values and CINeMA confidence ratings jointly, rather than treating ranking metrics as evidence of clinical superiority [30,79,80].

In our network, Pilates APP showed the largest favorable estimate and shared the highest-ranking position, with moderate confidence, which supports its cautious clinical consideration compared with interventions supported by lower-certainty evidence. STMC and ST also showed favorable estimates and high-ranking positions, but both comparisons were supported by low-confidence evidence. By contrast, STM showed a favorable signal with moderate confidence, supporting its cautious clinical consideration despite its lower position in the P-score ranking. Given the small differences in relative efficacy between the best-ranked interventions and the presence of moderate-confidence evidence for both exercise-based and manual therapy approaches, our findings do not support a categorical superiority of therapeutic exercise over manual therapy.

Previous literature has highlighted that the terms therapeutic exercise and manual therapy include highly heterogeneous interventions. This makes both clinical interpretation and estimation of relative effects difficult when pairwise comparisons or overly broad categories are used [13,14,21,22]. The contribution of the present study was to explore whether a more specific division of therapeutic exercise and manual therapy techniques could improve clinical interpretation.

This nodal division is particularly relevant for exercise interventions. Mat Pilates and apparatus-based Pilates share general principles, such as control, precision, breathing, and body awareness. However, apparatus use may modify movement assistance, sensorimotor feedback, and load progression, making their separation clinically justifiable [81]. Similarly, stabilization exercises with motor control are not equivalent to any lumbar stabilization program, because they include specific instructions, feedback, or retraining strategies aimed at lumbopelvic and trunk control [70,82,83]. Moreover, distinguishing stabilization/motor control from progressive strengthening was intended to avoid combining motor learning-oriented interventions with programs mainly focused on load, strength, or muscular endurance [18,84]. This separation was intended to improve clinical interpretability, although it also reduces the size of some nodes and requires cautious interpretation of the estimates.

The present findings are partly consistent with previous NMAs focused on therapeutic exercise. Fernández-Rodríguez et al. identified Pilates, strength training, core-based exercises, and mind–body exercise among the most favorable options for pain and disability in adults with chronic low back pain [16]. Hayden et al. also showed that some exercise modalities appear to be more effective than others, with Pilates and McKenzie ranking favorably for pain and functional limitation. However, they also emphasized the importance of dose, delivery format, and cointerventions [20]. Owen et al. and Li et al. reported similar findings, showing that different active exercise modalities may behave differently within a treatment network for chronic low back pain [15,17]. Zhao et al. further suggested that the effects of exercise depend not only on the type of intervention, but also on prescription parameters such as frequency, session duration, and total program duration [84].

Nevertheless, comparisons with these NMAs should be made with caution. In previous reviews, categories such as core-based exercise, stabilization, motor control, strengthening, or Pilates may have been partially grouped or overlapped. In our study, these interventions were separated into more specific nodes [16,17,18,19,20]. This methodological difference may explain why some interventions, such as STR, occupied a less prominent position in our network. This does not necessarily contradict previous evidence; rather, it may reflect the loss of the pooled effect produced when clinically related techniques are grouped together. Regarding MDT, its position in our network is consistent with Fernández-Rodríguez et al., where it did not show a clear advantage over control for this outcome [16]. In the NMA by Hayden et al., MDT ranked highest for disability, although its advantage was reduced after adjustment for dose and cointerventions [20]. Similarly, the low position of the STRET node is consistent with previous literature [16,17,20], although the limited number of comparisons in our network should also be considered.

The comparison with evidence directly contrasting therapeutic exercise and manual therapy should also be interpreted from this perspective. The Cochrane review on exercise for chronic low back pain showed that exercise is probably more effective than education or advice alone, and more effective than electrotherapy. However, it found no clear differences compared with manual therapy when these treatments were analyzed as broad comparator categories [21]. González-Gómez et al. found a small advantage of therapeutic exercise over manual therapy for long-term disability but concluded that the evidence was insufficient to establish conclusive differences between both modalities as isolated treatments. This was due to heterogeneity, the small number of studies, and the very low certainty of the evidence [22]. Our findings are consistent with this cautious interpretation, but they add a different perspective: the absence of categorical superiority between therapeutic families does not mean that all techniques behave similarly, nor that manual therapy should be interpreted as a homogeneous block.

The behavior of manual therapy within our network reinforces this interpretation. Soft tissue manipulation was among the interventions with a favorable signal compared with control and was the node with the highest relative certainty among the best-positioned interventions. By contrast, spinal manipulation showed a favorable signal of smaller magnitude and very low certainty. This difference suggests that the general category “manual therapy” may conceal different effects across specific manual techniques. External evidence also supports a cautious interpretation. Coulter et al. and de Zoete et al. showed that spinal manipulation or mobilization may improve pain and function in chronic low back pain, but these reviews do not clarify which specific manual technique provides greater relative value compared with specific exercise modalities [85,86]. Dos Santos et al. observed that adding manual therapy to exercise may improve disability in certain contexts. However, the evidence was limited and does not clearly establish which patients benefit, or which manual technique provides greater incremental value [87].

Dry needling was prespecified as a manual therapy node but could not be analyzed because no eligible RCT fulfilled the PICO and operational eligibility criteria required to populate this node in the disability network. This should not be interpreted as evidence that dry needling is ineffective for NSCLBP. Rather, it indicates a lack of eligible comparative evidence for the specific question addressed in this review: post-treatment self-reported disability measured with ODI or RMDQ, using an intervention contrast that allowed the effect of dry needling to be isolated or attributed to a balanced additive design. This distinction is clinically relevant because previous systematic evidence in chronic low back pain suggests that dry needling may have short-term effects on pain, particularly when combined with other therapies, but does not provide clear evidence of improvement in disability. Future trials should evaluate dry needling using clearly defined comparators, balanced co-interventions, adequate follow-up and standardized disability outcomes to allow its integration into future comparative evidence networks [88].

Variability in dose and intervention delivery may explain part of the heterogeneity observed in the network. Descriptive inspection of potential effect modifiers also showed variability across direct comparisons in baseline disability, age, sex distribution, BMI availability and methodological quality (Supplementary Table S6). The included programs differed in duration, frequency, supervision, progression, protocol fidelity, and use of home exercise (Supplementary Table S8). These differences are clinically relevant because exercise-prescription parameters, including frequency, session duration and total program duration, may influence treatment effects in chronic low back pain [84]. Similarly, therapeutic exercise progression in non-specific low back pain is heterogeneous and often insufficiently described, which may limit reproducibility and translation into clinical practice [89]. Recent meta-research using the i-CONTENT tool also showed that dosage and adherence are among the exercise-intervention domains with greater uncertainty in chronic low back pain trials [90]. Home-exercise delivery may be another relevant source of variability, as recent evidence suggests that unsupervised home exercise is less effective than supervised in-person exercise for short-term pain and functional disability in NSCLBP [91]. Finally, adherence should be interpreted as a complex, context-dependent implementation factor rather than as a simple binary variable; recent systematic evidence indicates that adherence to exercise therapy in NSCLBP is influenced by internal, external and intervention-related factors, including beliefs, motivation, supervision, feedback, program design, confidence and home-exercise support [92,93]. Therefore, two interventions classified within the same node may differ substantially in their real-world application. The nodes should be interpreted as clinically reasonable categories, but not as fully homogeneous interventions.

From a clinical perspective, self-reported disability is a broad outcome. This focus on disability is also supported by observational evidence showing that spinal pain is associated with functional decline and limitations in daily activities, although such evidence is contextual and not directly comparable to the intervention trials included in the present NMA [94]. It is influenced by pain, movement tolerance, perceived threat, self-efficacy, motor control, and functional participation [3,4]. This may explain why different interventions show similar improvements in disability at the end of the intervention, because of convergence through partially shared mechanisms, even if they are not equivalent. Pilates and stabilization/motor control programs may act through control, coordination, and progressive exposure to movement [81,83]. Manual techniques are currently explained through neurophysiological, contextual, and symptom-modulation mechanisms, rather than through an exclusively biomechanical interpretation. They may therefore influence pain, mechanical sensitivity, perceived threat, and movement tolerance [95,96]. However, this explanation should be considered exploratory, because the present NMA did not assess clinical mediators or physiological mechanisms.

### 4.1. Clinical Implications

From a clinical standpoint, the results are consistent with careful clinical consideration of structured, supervised interventions with potential for progression, particularly those based on Pilates APP, STMC, ST, and Pilates MAT. However, the favorable position of STM and the absence of clear statistically significant differences between these interventions prevent us from concluding that therapeutic exercise is globally superior to manual therapy for reducing self-reported disability at the end of the intervention. The findings are consistent with current recommendations, which favor non-surgical, person-centered care. In this approach, exercise is a therapeutic pillar, while manual therapy may be considered as part of a multimodal strategy when clinically justified [4,97,98].

In routine clinical care, the applicability of these findings will also be conditioned by the therapist’s familiarity with each technique, the patient’s expectations and preferences, and the feasibility of providing supervised or equipment-based programs in a given care setting. Therefore, treatment selection should integrate the overall certainty and applicability of the evidence with clinical reasoning and shared decision-making, rather than relying on the network estimates in isolation.

In patients with low initial tolerance to movement, high perceived threat, or symptoms that limit active participation, manual therapy may have a complementary role as a symptom-modulation strategy and as a means of improving movement tolerance. This may be particularly relevant when it is integrated with education and active progression. However, profile-based considerations should be understood as hypotheses for clinical reasoning and shared decision-making rather than as subgroup-specific treatment effects established by this NMA. Patient-level treatment-effect modifiers have been explored in persistent low back pain exercise trials using individual participant data meta-analysis, and baseline individual factors associated with outcomes after manual therapy have also been reviewed; however, these data do not establish subgroup-specific effects for the intervention nodes evaluated in the present network [99,100]. Available evidence also appears to support manual therapy primarily as a component of multimodal strategies rather than as a substitute for exercise or self-management [22,87,96,101].

### 4.2. Implications for Research

The results suggest several priorities for future research. In the short term, adequately powered head-to-head RCTs are needed between the nodes showing the most favorable signals, especially Pilates APP, STMC, ST, STM, and Pilates MAT. These trials should include adequate sample sizes, harmonized disability outcomes, predefined post-treatment and follow-up time points, and transparent reporting of dose, frequency, session duration, progression, supervision, adherence, protocol fidelity and home exercise. This is particularly relevant because exercise-prescription parameters may influence treatment effects in chronic low back pain [84], exercise progression is often heterogeneous and insufficiently described [89], and adherence is influenced by patient-, intervention- and context-related factors [92,93]. Improving intervention reporting and therapeutic standardization is therefore important to distinguish whether observed differences are attributable to the intervention type itself or to how the intervention is delivered.

In the longer term, future research should investigate treatment-effect modifiers and response subgroups using prespecified subgroup analyses or individual participant data approaches. Individual participant data meta-analysis has specifically examined exercise treatment-effect modifiers in persistent low back pain [99], and baseline individual factors associated with outcomes after manual therapy have recently been reviewed [100]; however, these approaches have not established subgroup-specific effects for the intervention nodes evaluated in the present NMA. Variables such as baseline disability, movement tolerance, kinesiophobia, symptom duration, expectations, self-efficacy, sensitisation, motor-control impairments, age, sex and BMI may help explain heterogeneity in response to exercise or manual therapy techniques. Future trials should also evaluate combined or sequential strategies, particularly those in which manual therapy is used as initial support for active exposure or to improve movement tolerance, without assuming in advance that its effect is independent of, or superior to, exercise [87].

### 4.3. Limitations

This study has several limitations that affect the interpretation of its findings. First, although the protocol included pain and function/disability, the present manuscript focused on self-reported disability at the end of the intervention. Therefore, no conclusions can be drawn about pain or about medium- or long-term effects. Second, the exclusion of diskogenic pain was based on the diagnostic labels and eligibility criteria reported in the original trials. Therefore, although studies explicitly recruiting participants with diskogenic or disc-related low back pain were excluded, some participants classified as having NSCLBP may have had unidentified disc-related changes, as most trials did not systematically use imaging to rule them out. Third, the network showed high heterogeneity, probably related to differences in population, intervention definitions, dose, supervision, outcome measures, and comparator characteristics. Potential effect modifiers and intervention-delivery characteristics were tabulated descriptively in Supplementary Tables S6 and S8, but they were not incorporated into adjusted network models because reporting was incomplete, heterogeneous and sparse across several comparisons. Therefore, residual intransitivity or confounding by intervention delivery cannot be excluded. Fourth, greater nodal specificity improves clinical interpretability, but it also reduces the size of some nodes, increases reliance on indirect evidence, and may make the analysis more sensitive to incomplete descriptions of intervention protocols. Fifth, dry needling could not be analyzed because no studies met the PICO and operational eligibility criteria required to populate this node in the disability network. As a result, the representation of manual therapy was limited to spinal manipulation and soft tissue manipulation. Sixth, the use of a PEDro score threshold as an eligibility criterion is another limitation of this review. Although the PEDro <6 exclusion criterion was prespecified in the registered protocol and was applied before quantitative synthesis, excluding trials on the basis of a summary methodological score may introduce quality-related selection bias. Finally, the CINeMA assessment and the ±0.20 SMD decision threshold were not specified in the original PROSPERO protocol. They were introduced after registration as a methodological refinement to improve transparency in the interpretation of network estimates. This refinement did not modify eligibility criteria, intervention nodes, the statistical model, or effect estimates. Because several automatic CINeMA judgments may not fully capture network-level instability identified by design-level sensitivity analyses, contribution profiles, and the net heat plot, final confidence ratings were conservatively adjudicated. Alternative thresholds or adjudication rules could have led to different confidence ratings; therefore, the CINeMA ratings should be interpreted as conservative and threshold-dependent. Reporting bias was not downgraded because no comparison-specific missing-evidence signal was identified, although small-study effects or missing evidence cannot be completely excluded. These limitations require the findings to be interpreted as a more precise reading of the available evidence rather than as a definitive hierarchy of efficacy.

## 5. Conclusions

This network meta-analysis suggests that several specific therapeutic exercise and manual therapy techniques may reduce post-treatment self-reported disability in adults with chronic non-specific low back pain when compared with control. Among the interventions with the most favorable point estimates, equipment-based Pilates and soft tissue manipulation showed favorable estimates supported by moderate-confidence evidence, whereas stabilization exercises with motor control and stabilization exercises were supported by low-confidence evidence, and Pilates Mat by very low-confidence evidence. However, the high heterogeneity of the network, the absence of clear statistically significant differences between the interventions with the most favorable estimates, and the threshold-dependent CINeMA adjudication require cautious interpretation. Overall, the results should be considered signals of potential post-treatment benefit rather than a definitive hierarchy of efficacy, and they do not support a categorical superiority of therapeutic exercise over manual therapy.

## Figures and Tables

**Table 1:** CINeMA-Based Summary of Findings and Final Confidence Ratings for Post-Treatment Disability Versus CTRL.

Intervention vs. CTRL	Evidence	Direct Studies	NMA SMD [95% CI]	95% PrI	P-Score	Final Confidence
Pilates APP vs. CTRL	Mixed	2	−0.879 [−1.156, −0.601]	[−1.434, −0.323]	0.92	Moderate
STMC vs. CTRL	Mixed	4	−0.856 [−1.044, −0.667]	[−1.370, −0.342]	0.92	Low
ST vs. CTRL	Mixed	2	−0.757 [−0.988, −0.527]	[−1.290, −0.225]	0.83	Low
STM vs. CTRL	Mixed	2	−0.681 [−0.998, −0.365]	[−1.259, −0.104]	0.75	Moderate
Pilates MAT vs. CTRL	Mixed	4	−0.643 [−0.851, −0.436]	[−1.165, −0.121]	0.72	Very low
GE vs. CTRL	Indirect	0	−0.392 [−0.619, −0.164]	[−0.922, 0.139]	0.52	Very low
SM vs. CTRL	Direct	4	−0.399 [−0.639, −0.160]	[−0.935, 0.137]	0.51	Very low
STR vs. CTRL	Mixed	2	−0.312 [−0.522, −0.101]	[−0.835, 0.211]	0.44	Very low
MDT vs. CTRL	Mixed	1	−0.138 [−0.461, 0.186]	[−0.719, 0.444]	0.32	Low
STRET vs. CTRL	Direct	1	0.047 [−0.467, 0.561]	[−0.667, 0.760]	0.19	Very low
UC vs. CTRL	Indirect	0	0.197 [−0.214, 0.608]	[−0.441, 0.835]	0.10	Very low
BS vs. CTRL	Indirect	0	0.269 [−0.296, 0.833]	[−0.485, 1.022]	0.08	Very low

Note. SMD = standardized mean difference; CI = confidence interval; PrI = prediction interval; CTRL = control; APP = apparatus-based Pilates; MAT = mat Pilates; STMC = stabilization exercises with motor control; ST = stabilization exercises; STM = soft tissue manipulation; SM = spinal manipulation; STR = strengthening or resistance exercise; MDT = McKenzie/Mechanical Diagnosis and Therapy; STRET = stretching; UC = usual care; BS = back school; GE = general exercise; Final confidence ratings were conservatively adjudicated from automatic CINeMA domain-level judgments and additional network diagnostics using a downgrade-only GRADE-style approach within the CINeMA framework. The ±0.20 SMD threshold was defined before final confidence adjudication and should not be interpreted as an ODI- or RMDQ-specific minimal clinically important difference. P-score values describe ranking only. The adjudication criteria are reported in Supplementary Material Text S1.

## Data Availability

Data supporting the findings of this study are available within the article and its Supplementary Materials. Additional extracted datasets and analysis files can be made available from the corresponding author upon reasonable request.

## Supplementary Materials

The following supporting information can be downloaded at https://www.mdpi.com/article/10.3390/jcm15124809/s1; Table S1a,b, database search strategies and Google Scholar/gray-literature searches; Table S2, operational definitions of intervention nodes; Table S3, excluded full-text records and reasons for exclusion; Table S4a,b, general study characteristics and distribution of intervention nodes; Table S5, characteristics of included comparisons and intervention extraction; Table S6, potential effect modifiers and transitivity assessment across direct comparisons; Table S7, direct–indirect decomposition of network estimates; Table S8, intervention delivery characteristics; Table S9, comparison between the registered PROSPERO protocol and final review methods; Figure S1, risk-of-bias assessment; Figure S2, evidence-flow and network-architecture diagnostics; Figure S3, comparison-adjusted funnel plot; Supplementary Material Text S1, CINeMA adjudication rules and rationale; Supplementary Checklist S1, PRISMA 2020 Checklist. Reference [102] are cited in the supplementary materials.

## Abbreviations

The following abbreviations are used in this manuscript:

NSCLBP	Non-Specific Chronic Low Back Pain
LBP	Low Back Pain
NMA	Network Meta-Analysis
ST	Stabilization Exercises
STMC	Stabilization Exercises with Motor Control
BS	Back School
GE	General Exercise
STR	Strengthening Exercises
STRET	Stretching
UC	Usual Care
CTRL	Control
STM	Soft Tissue Manipulation
SM	Spinal Lumbar Manipulation
DN	Dry Needling
PMAT	Pilates Mat
PAPP	Pilates Apparatus
MDT	McKenzie Method
ODI	Oswestry Disability Index
RMDQ	Roland–Morris Disability Questionnaire
