# Supplementary material for: Comparative Effects of Therapeutic Exercise and Manual Therapy Techniques on Self-Reported Disability in Chronic Non-Specific Low Back Pain: A Network Meta-Analysis
Source: J Clin Med. 2026 Jun 21;15(12):4809. doi: 10.3390/jcm15124809 (PMC13301920; doi:10.3390/jcm15124809)
Supplement: Supplementary file 1 [file jcm-15-04809-s001.zip › jcm-4372196-supplementary.pdf]

Supplementary Material

Tables

Table S1a

Database search strategies and records included in the PRISMA flow diagram

Source	Search strategy or filters	Displayed results	Records included in PRISMA
PubMed	(("Low Back Pain"[MeSH Terms] OR "Chronic Low Back Pain"[Text Word]) AND ("Adult"[MeSH Terms] OR "Young Adult"[MeSH Terms] OR "Middle Aged"[MeSH Terms])) AND ("Exercise Therapy"[MeSH Terms] OR "Resistance Training"[MeSH Terms] OR "Pilates Exercise"[MeSH Terms] OR "Exercise Movement Techniques"[MeSH Terms] OR "Manipulation, Spinal"[MeSH Terms] OR "Musculoskeletal Manipulations"[MeSH Terms] OR "Dry Needling"[MeSH Terms] OR "McKenzie method"[Text Word] OR "Motor Control Exercise"[Text Word] OR "Stabilization Exercise"[Text Word] OR "Manual Therapy"[Text Word]) AND ("Disability Evaluation"[MeSH Terms] OR "Roland-Morris Disability Questionnaire"[Text Word] OR "Oswestry Disability Index"[Text Word] OR "RMDQ"[Text Word] OR "ODI"[Text Word]) AND (randomized controlled trial[Publication Type] OR clinical trial[Publication Type])	509	509
CENTRAL	#1 Population: [mh "Low Back Pain"] OR "chronic low back pain":ti,ab,kw<br>#2 Age: [mh Adult] OR [mh "Young Adult"] OR [mh "Middle Aged"]<br>#3 Exercise intervention: [mh "Exercise Therapy"] OR [mh "Resistance Training"] OR [mh "Pilates Exercise"] OR "McKenzie method":ti,ab,kw OR "Motor Control Exercise":ti,ab,kw OR "Stabilization Exercise":ti,ab,kw<br>#4 Manual/dry-needling intervention: [mh "Manipulation, Spinal"] OR [mh "Musculoskeletal Manipulations"] OR [mh "Dry Needling"] OR "Manual Therapy":ti,ab,kw<br>#5 Outcomes: [mh "Disability Evaluation"] OR "Roland-Morris":ti,ab,kw OR "Oswestry":ti,ab,kw OR "RMDQ":ti,ab,kw OR "ODI":ti,ab,kw<br>Final combination: #1 AND #2 AND (#3 OR #4) AND #5	573	573
PEDro search 1	Therapy: strength training<br>Body part: stretching, mobilisation, manipulation, massage<br>Subdiscipline: musculoskeletal<br>Topic: chronic pain<br>Method: clinical trial<br>Minimum PEDro score: 6	398	394
PEDro search 2	Therapy: strength training<br>Body part: lumbar spine, sacro-iliac joint, or pelvis<br>Subdiscipline: musculoskeletal<br>Topic: chronic pain<br>Method: clinical trial<br>Minimum PEDro score: 6	385	380
PEDro total	Sum of PEDro exportable/retrieved records used in the PRISMA flow diagram.	783	774
SciELO	(low back pain) AND (chronic) AND (randomized) AND (adult) OR (pilates based exercises) OR (resistance training) OR (McKenzie therapy) OR (stabilization exercises) OR (dry needling) OR (spinal manipulation) OR (musculoskeletal manipulation)	49	49
Total databases	Sum of database records. The PRISMA count uses exportable/retrieved records for PEDro.	1914	1905
Registers	No trial registers were searched/reported in the PRISMA flow diagram.	0	0

Note. Searches were recorded up to 31 March 2026. For PEDro, interface-displayed results were 398 and 385; the values in parentheses in the original search log were interpreted as exportable/retrieved records and were therefore used for the PRISMA count: 394 + 380 = 774. Minor typographical inconsistencies in source names and controlled-filter labels were standardized for reporting; counts were unchanged.

Table S1b

Google Scholar and grey-literature searches included under identification via other methods

Topic	Google Scholar search query	Estimated/displayed results	Exportable records screened and included in PRISMA
Spinal manipulation	allintitle: "spinal manipulation" "randomized controlled trial" "chronic low back pain"	10	10
Strengthening	allintitle: "strengthening" "randomized controlled trial" "chronic low back pain"	24	14
Pilates	allintitle: "pilates" "randomized controlled trial" "chronic low back pain"	46	39
McKenzie	allintitle: "McKenzie" "randomized controlled trial" "chronic low back pain"	27	17
Stabilization exercises	allintitle: "stabilization exercises" "randomized controlled trial" "chronic low back pain"	38	28
Dry needling	allintitle: "dry needling" "randomized controlled trial" "chronic low back pain"	11	11
Soft-tissue manipulation	Search term recorded; no records retrieved.	0	0
Total Google Scholar / grey literature	Sum of exportable records screened and used under identification via other methods in the PRISMA flow diagram.	156	119

Note. Google Scholar reports approximate/estimated results. To maintain consistency with the PRISMA flow diagram, only exportable records retrieved and screened were counted. The exportable records came to 119, whereas estimated/displayed results summed to 156.

PRISMA consistency summary

PRISMA source category	Calculation	Records
Databases	PubMed 509 + CENTRAL 573 + PEDro 774 + SciELO 49	1,905
Other methods	Google Scholar / grey literature exportable records	119
Registers	None reported	0

Abbreviations. CENTRAL = Cochrane Central Register of Controlled Trials; PRISMA = Preferred Reporting Items for Systematic Reviews and Meta-Analyses.

Table S2

Operational Definitions of the Intervention Nodes Included in the Network Meta-Analysis

Intervention node	Operational definition
Spinal manipulation	Physiotherapy interventions including manual techniques applied to the spine with the aim of modifying spinal joint function or mobility.
Soft tissue manipulation	Physiotherapy techniques involving direct manual treatment of muscular or fascial tissues, including massage, myofascial techniques, or other soft tissue procedures.
Stabilization exercises (ST)	Classical lumbar stabilization exercises that do not include specific feedback, explicit motor control instructions, or structured retraining of movement patterns.
Stabilization exercises with motor control (STMC)	Lumbar stabilization exercises that include specific feedback, explicit instructions, or structured strategies aimed at improving motor control of the lumbar, pelvic, or trunk region.
Mat Pilates (Pilates MAT)	Pilates-based exercises performed predominantly on a mat or floor, with or without minor accessories such as balls, rollers, elastic bands, or magic circles.
Equipment-based Pilates (Pilates APP)	Pilates-based interventions in which specific Pilates equipment, such as the Reformer, Cadillac, Chair, Barrel, or other spring-resistance apparatus, represents a structural and recurrent component of the program.
Dry needling	Invasive intervention involving the insertion of needles into myofascial trigger points located in muscles of the lumbar and/or pelvitrochanteric region.
Progressive strengthening or resistance exercise	Exercises are designed to progressively increase strength, endurance, or muscle mass of the lumbar, pelvic, or trunk region through graded resistance or load progression.
General exercise (GE)	Exercise interventions combining at least two types of exercise, such as aerobic training, strengthening, mobility, or flexibility exercises, where no single component is clearly dominant or classifiable under another specific node.
Stretching (STRET)	Interventions in which stretching is the only active therapeutic component.
Back school (BS)	Educational or therapeutic programs based on back school principles, including postural education, ergonomic advice, and/or structured exercise booklets typical of this approach.
Control	Minimal intervention, placebo, sham intervention, waiting list, or minimal education-only comparator not involving a structured active therapeutic exercise or manual therapy program.
Usual care (UC)	Multicomponent physiotherapy or standard clinical care including several therapeutic modalities, such as electrotherapy, thermotherapy, general advice, or non-specific physiotherapy treatment.

Note. Intervention nodes were defined to ensure clinical coherence and reproducibility within the network meta-analysis. Studies including additive designs were considered eligible only when co-interventions were balanced across arms, so that the differential effect could be attributed to the specific intervention node under evaluation. Equipment-based Pilates includes mixed mat and apparatus programs when Pilates equipment represents a relevant and recurrent component of the intervention.

Table S3

Articles Excluded After Full-Text Assessment and Reasons for Exclusion

Record No. (merged)	Decision	Exclusion criterion	First author	Year	Title	Journal/Source	DOI	URL	Language	Authors	Reviewers
1	Excluded	Ineligible intervention: multimodal protocol	Divya	2021	Effect of lumbar stabilization exercises and thoracic mobilization with strengthening exercises on pain level, thoracic kyphosis, and functional disability in chronic low back pain	Journal of Complementary & Integrative Medicine 2021 Jun;18(2):419-424	10.1515/jcim-2019-0327	https://pubmed.ncbi.nlm.nih.gov/32712591/	English	Divya; Parveen A; Nuhmani S; Ejaz Hussain M; Hussain Khan M	MRG, JMV
2	Excluded	Wrong population	Farajzadeh F	2017	Effects of McGill stabilization exercise on pain and disability, range of motion and dynamic balance indices in patients with chronic nonspecific low back pain	Journal of Babol University of Medical Sciences 2017 Oct;19(10):21-27	10.1589/jpts.30.481	https://pubmed.ncbi.nlm.nih.gov/29706690/	English	Farajzadeh F; Ghaderi F; Asghari Jafarabadi M; Azghani MR; Eteraf Oskoui MA; Rezaie M; Ghorbanpour A	MRG, JMV
3	Excluded	Methodological quality: PEDro score lower than 6 (PEDro < 6)	Stankovic A	2012	Lumbar stabilization exercises in addition to strengthening and stretching exercises reduce pain and increase function in patients with chronic low back pain: randomized clinical open-label study	Turkiye Fiziksel Tip ve Rehabilitasyon Dergisi [Turkish Journal of Physical Medicine and Rehabilitation] 2012;58(3):177-183	10.4274/tftr.22438	https://www.cochranelibrary.com/central/doi/10.1002/central/CN-00901821/full	English	Stankovic A; Lazovic M; Kocic M; Dimitrijevic L; Stankovic I; Zlatanovic D; Dimitrijevic I	MRG, JMV
4	Excluded	Ineligible intervention: multimodal protocol	Geisser ME	2005	A randomized, controlled trial of manual therapy and specific adjuvant exercise for chronic low back pain	The Clinical Journal of Pain 2005 Nov-Dec;21(6):463-470	10.1097/01.ajp.0000135237.89834.23	https://pubmed.ncbi.nlm.nih.gov/16215330/	English	Geisser ME; Wiggert EA; Haig AJ; Colwell MO	MRG, JMV
5	Excluded	Acute/subacute pain: pain duration of less than 12 weeks	Lalkate S	2020	A comparative study effectiveness of conventional physiotherapy versus yoga therapy on pain, core muscle endurance, lumbar flexion range of motion and functional disability in patients with chronic mechanical low back pain	Indian Journal of Physiotherapy and Occupational Therapy 2020 Oct-Dec;14(4):177-182	10.37506/ijpot.v14i4.11322	https://medicopublication.com/index.php/ijpot/article/view/11322	English	Lalkate S; Agrawal R; Agashe GK	MRG, JMV
6	Excluded	Ineligible intervention: multimodal protocol	Udermann BE	2004	Combining lumbar extension training with McKenzie therapy: effects on pain, disability, and psychosocial functioning in chronic low back pain patients	Gundersen Lutheran Medical Journal 2004 Sep;3(2):7-12		https://convergemedtech.com/wp-content/uploads/2025/02/Combining-lumbar-extension-training-with-McKenzie-therapy-Effects-on-pain-disability-and-psychosocial-functioning-in-chronic-low-back-pain-patients.pdf	English	Udermann BE; Mayer JM; Donelson RG; Graves JE; Murray SR	MRG, JMV
7	Excluded	Acute/subacute pain: pain duration of less than 12 weeks	Petersen T	2007	One-year follow-up comparison of the effectiveness of McKenzie treatment and strengthening training for patients with chronic low back pain: outcome and prognostic factors [with consumer summary]	Spine 2007 Dec 15;32(26):2948-2956	10.1097/BRS.0b013e31815cda4a	https://pubmed.ncbi.nlm.nih.gov/18091486/	English	Petersen T; Larsen K; Jacobsen S	MRG, JMV
8	Excluded	Methodological quality: PEDro score lower than 6 (PEDro < 6)	Ghroubi S	2007	Les lombalgiques chroniques et manipulations vertebrales. Etude prospective a propos de 64 cas (Chronic low back pain and vertebral manipulation) [French]	Annales de Readaptation et de Medecine Physique 2007 Oct;50(7):570-576	10.1016/j.annrmp.2007.02.012	https://www.sciencedirect.com/science/article/abs/pii/S0168605407000487	French	Ghroubi S; Elleuch H; Baklouti S; Elleuch MH	MRG, JMV
9	Excluded	Methodological quality: PEDro score lower than 6 (PEDro < 6)	Goldby LJ	2006	A randomized controlled trial investigating the efficiency of musculoskeletal physiotherapy on chronic low back disorder [with consumer summary]	Spine 2006 May 1;31(10):1083-1093	10.1097/01.brs.0000216464.37504.64	https://pubmed.ncbi.nlm.nih.gov/16648741/	English	Goldby LJ; Moore AP; Doust J; Trew ME	MRG, JMV
10	Excluded	Acute/subacute pain: pain duration of less than 12 weeks	Hansen FR	1993	Intensive, dynamic back-muscle exercises, conventional physiotherapy, or placebo-control treatment of low-back pain. A randomized, observer-blind trial	Spine 1993 Jan;18(1):98-108	10.1097/00007632-199301000-00015	https://pubmed.ncbi.nlm.nih.gov/8434332/	English	Hansen FR; Bendix T; Skov P; Jensen CV; Kristensen JH; Krohn L; Schioeler H	MRG, JMV
11	Excluded	Ineligible intervention: multimodal protocol	Khalil TM	1992	Stretching in the rehabilitation of low-back pain patients	Spine 1992 Mar;17(3):311-317	10.1097/00007632-199203000-00012	https://pubmed.ncbi.nlm.nih.gov/1533060/	English	Khalil TM; Asfour SS; Martinez LM; Waly SM; Rosomoff RS; Rosomoff HL	MRG, JMV
12	Excluded	Wrong outcome	Hasanpour-Dehkordi, Ali	2017	A comparison of the effects of Pilates and McKenzie training on pain and general health in men with chronic low back pain: a randomized trial	Indian journal of palliative care	10.4103/0973-1075.197945	https://pubmed.ncbi.nlm.nih.gov/28216860/	English	Hasanpour-Dehkordi, Ali; Dehghani, Arman; Solati, Kamal	MRG, JMV
13	Excluded	Wrong outcome	Hosseinifar, Mohammad	2013	The effects of stabilization and McKenzie exercises on transverse abdominis and multifidus muscle thickness, pain, and disability: a randomized controlled trial in nonspecific chronic low back pain	Journal of physical therapy science	10.1589/jpts.25.1541	https://pubmed.ncbi.nlm.nih.gov/24409016/	English	Hosseinifar, Mohammad; Akbari, Mohammad; Behtash, Hamid; Amiri, Mohsen; Sarrafzadeh, Javad	MRG, JMV
14	Excluded	Wrong outcome	Halliday, Mark H	2016	A randomized controlled trial comparing the McKenzie method to motor control exercises in people with chronic low back pain and a directional preference	Journal of Orthopaedic & Sports Physical Therapy	10.2519/jospt.2016.6379	https://pubmed.ncbi.nlm.nih.gov/27170524/	English	Halliday, Mark H; Pappas, Evangelos; Hancock, Mark J; Clare, Helen A; Pinto, Rafael Z; Robertson, Gavin; Ferreira, Paulo H	MRG, JMV
15	Excluded	Wrong outcome	Halliday, Mark H	2019	A randomized clinical trial comparing the McKenzie method and motor control exercises in people with chronic low back pain and a directional preference: 1-year follow-up	Physiotherapy	10.1016/j.physio.2018.12.004	https://pubmed.ncbi.nlm.nih.gov/31204031/	English	Halliday, Mark H; Pappas, Evangelos; Hancock, Mark J; Clare, Helen A; Pinto, Rafael Z; Robertson, Gavin; Ferreira, Paulo H	MRG, JMV
16	Excluded	Study not retrievable	Hosseinifar, Mohammad	2009	The effects of McKenzie and lumbar stabilization exercises on the improvement of function and pain in patients with chronic low back pain: a randomized controlled trial	2009		https://scispace.com/papers/the-effects-of-mckenzie-and-lumbar-stabilization-exercises-18ud103l4w	Arabic	Hosseinifar, Mohammad; Akbari, Asghar; SHAHRAKINASAB, AAF	MRG, JMV
17	Excluded	Wrong publication type	Halliday, Mark H	2015	A randomized controlled trial comparing McKenzie therapy and motor control exercises on the recruitment of trunk muscles in people with chronic low back pain: a trial protocol	Physiotherapy	10.1016/j.physio.2014.07.001	https://pubmed.ncbi.nlm.nih.gov/25442673/	English	Halliday, Mark H; Ferreira, Paulo H; Hancock, Mark J; Clare, Helen A	MRG, JMV
18	Excluded	Wrong population	Ghanjal, Ali	2018	INVESTIGATING THE EFFECT OF NEUROMUSCULAR FACILITATION AND STABILIZATION EXERCISES ON PAIN, ALGOPHOBIA, KINESIOPHOBIA AND NECK RANGE OF MOTION IN PATIENTS WITH CHRONIC NON-SPECIFIC NECK PAIN. Akbari-Fakhrabadi M, Kordi R. The role of a multistep core stability exercise program in the treatment of...	Asian spine journal	ISSN 2229-5402	https://pharmacophorejournal.com/article/investigating-the-effect-of-neuromuscular-facilitation-and-stabilization-exercises-on-pain-algophobia-kinesiophobia-and-neck-range-of-motion-in-patients-with-chronic-non-specific-n...	English	Ghanjal, Ali; Ashtiani, Ahmadreza Askari; Hatef, Boshra; Nouraeisarjo, Salman	MRG, JMV
19	Excluded	Methodological quality: PEDro score lower than 6 (PEDro < 6)	Chakraborty, Jayanta		Comparative Study of Motor Control Exercises and Global Core Stabilization Exercises on Pain, ROM and Function in Subjects		ISSN: 2249-9571	https://www.ijhsr.org/IJHSR_Vol.9_Issue.8_Aug2019/18.pdf	English	Chakraborty, Jayanta; Kumar, Pravin; Sarkar, Bibhuti	MRG, JMV

Record No. (merged)	Decision	Exclusion criterion	First author	Year	Title	Journal/Source	DOI	URL	Language	Authors	Reviewers
					with Chronic Nonspecific Low Back Pain-A Randomized Clinical Trial						
20	Excluded	Methodological quality: PEDro score lower than 6 (PEDro < 6)	Fernández, EM		Effectiveness of the Back School and the Pilates Method in disability and pain of patients with chronic non-specific low back pain. Randomized controlled clinical trial. López-Mesa MM (1)(2), Cabrerizo-Fernández JJ (1), López-Román A (3), Rodríguez		10.21203/rs.3.rs-27957/v1	https://europepmc.org/article/ppr/ppr219780	English	Fernández, EM	MRG, JMV
21	Excluded	No ≥4-week follow-up	Dsa, Cassandra F	2014	To assess the effect of modified pilates compared to conventional core stabilization exercises on pain and disability in chronic non-specific low back pain-randomized controlled trial	Indian Journal of Physiotherapy and Occupational Therapy	10.5958/0973-5674.2014.00382.7	https://indianjournals.com/article/ijpot-8-3-039	English	Dsa, Cassandra F; Rengaramanujam, Kanagaraj; Kudchadkar, Mahendra S	MRG, JMV
22	Excluded	Wrong study design	Clark, Brian C	2018	A randomized control trial to determine the effectiveness and physiological effects of spinal manipulation and spinal mobilization compared to each other and a sham condition in patients with chronic low back pain: Study protocol for The RELIEF Study	Contemporary clinical trials	10.1016/j.cct.2018.05.012	https://pubmed.ncbi.nlm.nih.gov/29792940/	English	Clark, Brian C; Russ, David W; Nakazawa, Masato; France, Christopher R; Walkowski, Stevan; Law, Timothy D; Applegate, Megan; Mahato, Niladri; Lietkam, Samuel; Odenthal, James	MRG, JMV
23	Excluded	Wrong outcome	Sarker, Kanchan	2020	Comparative clinical effects of spinal manipulation, core stability exercise, and supervised exercise on pain intensity, segmental instability, and health-related quality of life among patients with chronic nonspecific low back pain: A randomized control trial	Journal of Natural Science, Biology and Medicine	10.4103/jnsbm.JNSBM_101_19	https://jnsbm.org/wp-content/uploads/2021/07/JNatScBiolMed-11-1-27.pdf	English	Sarker, Kanchan; Sethi, Jasobanta; Mohanty, Umasankar	MRG, JMV
24	Excluded	Methodological quality: PEDro score lower than 6 (PEDro < 6)	Sarker, Kanchan Kumar	2017	Effect of spinal manipulation on specific changes in segmental instability, pain sensitivity and health-related quality of life among patients with chronic non-specific low back pain-A randomized clinical trial	Annual Research & Review in Biology	10.9734/ARRB/2017/35926	https://journalarrb.com/index.php/ARRB/article/view/1036	English	Sarker, Kanchan Kumar; Sethi, Jasobanta; Mohanty, Umasankar	MRG, JMV
25	Excluded	Ineligible intervention: multimodal protocol	Haas, M	2012	OA06. 04. Dose-response of spinal manipulation for chronic low back pain: pain and disability outcomes from a randomized controlled trial	BMC Complementary and Alternative Medicine	10.1186/1472-6882-12-S1-O24	https://pmc.ncbi.nlm.nih.gov/articles/PMC3373330/		Haas, M; Vavrek, D; Peterson, D	MRG, JMV
26	Excluded	Wrong outcome	Miller ER	2005	A comparison of the McKenzie approach to a specific spine stabilization program for chronic low back pain	The Journal of Manual & Manipulative Therapy 2005;13(2):103-112	10.1179/106698105790824996	https://www.tandfonline.com/doi/abs/10.1179/106698105790824996	English	Miller ER; Schenk RJ; Karnes JL; Rousselle JG	MRG, JMV
27	Excluded	Ineligible intervention: multimodal protocol	Niemisto L	2005	Cost-effectiveness of combined manipulation, stabilizing exercises, and physician consultation compared to physician consultation alone for chronic low back pain: a prospective randomized trial with 2-year follow-up [with consumer summary]	Spine 2005 May 15;30(10):1109-1115	10.1097/01.brs.0000162569.00685.7b	https://pubmed.ncbi.nlm.nih.gov/15897822/	English	Niemisto L; Rissanen P; Sarna S; Lahtinen-Suopanki T; Lindgren KA; Hurri H	MRG, JMV
28	Excluded	Wrong outcome	Ajimsha MS	2014	Effectiveness of myofascial release in the management of chronic low back pain in nursing professionals	Journal of Bodywork and Movement Therapies 2014 Apr;18(2):273-281	10.1016/j.jbmt.2013.05.007	https://pubmed.ncbi.nlm.nih.gov/24725797/	English	Ajimsha MS; Daniel B; Chithra S	MRG, JMV
29	Excluded	Methodological quality: PEDro score lower than 6 (PEDro < 6)	Ali S	2013	Effectiveness of core stabilization exercises versus McKenzie's exercises in chronic lower back pain	Medical Forum Monthly 2013 Dec;24(12):82-85	10.12669/PJMS.334.12664	https://www.academia.edu/66779658/Effectiveness_of_Core_Stabilization_Exercises_versus_Mckenzie_s_Exercises_in_Chronic_Lower_Back_Pain	English	Ali S; Ali SM; Memon KN	MRG, JMV
30	Excluded	Methodological quality: PEDro score lower than 6 (PEDro < 6)	You Y-L	2015	The effect of six weeks of sling exercise training on trunk muscular strength and endurance for clients with low back pain	Journal of Physical Therapy Science 2015 Aug;27(8):2591-2596	10.1589/jpts.27.2591	https://pubmed.ncbi.nlm.nih.gov/26356255/	English	You Y-L; Su T-K; Liaw L-J; Wu W-L; Chu I-H; Guo L-Y	MRG, JMV
31	Excluded	Methodological quality: PEDro score lower than 6 (PEDro < 6)	Steele J	2013	A randomised controlled trial of limited range of motion lumbar extension exercise in chronic low back pain [with consumer summary]	Spine 2013 Jul 1;38(15):1245-1252	10.1097/BRS.0b013e318291b526	https://pubmed.ncbi.nlm.nih.gov/23514876/	English	Steele J; Bruce-Low S; Smith D; Jessop D; Osborne N	MRG, JMV
32	Excluded	Wrong publication type	Cecchi F	2012	Predictors of functional outcome in patients with chronic low back pain undergoing back school, individual physiotherapy or spinal manipulation [with consumer summary]	European Journal of Physical and Rehabilitation Medicine 2012 Sep;48(3):371-378		https://pubmed.ncbi.nlm.nih.gov/22569488/	English	Cecchi F; Negrini S; Pasquini G; Paperini A; Conti AA; Chiti M; Zaina F; Macchi C; Molino-Lova R	MRG, JMV
33	Excluded	Methodological quality: PEDro score lower than 6 (PEDro < 6)	Tsauo J-Y	2009	The effectiveness of a functional training programme for patients with chronic low back pain -- a pilot study	Disability and Rehabilitation 2009;31(13):1100-1106	10.1080/09638280802511047	https://pubmed.ncbi.nlm.nih.gov/19802926/	English	Tsauo J-Y; Chen W-H; Liang H-W; Jang Y	MRG, JMV
34	Excluded	Methodological quality: PEDro score lower than 6 (PEDro < 6)	Marshall P	2008	Self-report measures best explain changes in disability compared with physical measures after exercise rehabilitation for chronic low back pain [with consumer summary]	Spine 2008 Feb 1;33(3):326-338	10.1097/BRS.0b013e31816233eb	https://pubmed.ncbi.nlm.nih.gov/18303467/	English	Marshall P; Murphy B	MRG, JMV
35	Excluded	Methodological quality: PEDro score lower than 6 (PEDro < 6)	Mohan Kumar G	2020	Comparative effect of Mulligan's mobilisation versus stabilisation exercise on chronic nonspecific low back pain: a pilot study	Indian Journal of Public Health Research & Development 2020 Feb;11(2):1283-1288	10.37506/v11/i1/2020/ijphrd/194019	https://medicopublication.com/index.php/ijphrd/article/view/875	English	Mohan Kumar G; Paul J; Sundaram MS; Mahendranath P	MRG, JMV
36	Excluded	No ≥4-week follow-up	Celenay ST	2019	Adding connective tissue manipulation to physiotherapy for chronic low back pain improves pain, mobility, and well-being: a randomized controlled trial	Journal of Exercise Rehabilitation 2019 Apr;15(2):308-315	10.12965/jer.1836634.317	https://pubmed.ncbi.nlm.nih.gov/31111018/	English	Celenay ST; Kaya DO; Ucurum SG	MRG, JMV
37	Excluded	No ≥4-week follow-up	Endamli DB	2019	Investigation of fascial treatment effectiveness on pain, flexibility, functional level, and kinesiophobia in patients with chronic low back pain	Physiotherapy Quarterly 2019;27(3):1-5	10.5114/pq.2019.86461	https://physioquart.awf.wroc.pl/pdf-109507-103592?filename=Investigation-of-fascial-.pdf	English	Endamli DB; Bayramlar K; Turhan B	MRG, JMV
38	Excluded	Methodological quality: PEDro score lower than 6 (PEDro < 6)	da Silva PHB	2018	The effect of the Pilates method on the treatment of chronic low back pain: a clinical, randomized, controlled study	Brazilian Journal of Pain 2018 Jan-Mar;1(1):21-28	10.5935/2595-0118.20180006	https://www.scielo.br/j/brjp/a/wg6KjcSSQrW6QPYV69TWMjB/?lang=en	English	da Silva PHB; da Silva DF; Oliveira JKS; de Oliveira FB	MRG, JMV
39	Excluded	Acute/subacute pain: pain duration of less than 12 weeks	Domingo-Esteban S	2017	Posibles beneficios de la pinza rodada en el dolor y la incapacidad en la lumbalgia inespecifica cronica (Potential benefits of skin rolling in pain and disability in non-specific chronic low back pain) [Spanish]	Fisioterapia 2017 Mar-Apr;39(2):60-67	10.1016/j.ft.2016.09.001	https://www.elsevier.es/es-revista-fisioterapia-146-articulo-posibles-beneficios-pinza-rodada-el-S0211563816301006	Spanish	Domingo-Esteban S; Navas-Camara FJ	MRG, JMV
40	Excluded	Methodological quality: PEDro score lower than 6 (PEDro < 6)	Branchini M	2016	Fascial manipulation for chronic aspecific low back pain: a single blinded randomized controlled trial	F1000Research 2016 Nov 3;4(1208):Epub	10.12688/f1000research.6890.2	https://pubmed.ncbi.nlm.nih.gov/26834998/	English	Branchini M; Lopopolo F; Andreoli E; Loreti I; Marchand AM; Stecco A	MRG, JMV
41	Excluded	Methodological quality: PEDro score lower than 6 (PEDro < 6)	Szulc P	2015	Impact of McKenzie method therapy enriched by muscular energy techniques on subjective and objective parameters related to spine	Medical Science Monitor 2015 Sep 29;21:2918-2932	10.12659/MSM.894261	https://pubmed.ncbi.nlm.nih.gov/26418868/	English	Szulc P; Wendt M; Waszak M; Tomczak M; Cieslik K; Trzaska T	MRG, JMV

Record No. (merged)	Decision	Exclusion criterion	First author	Year	Title	Journal/Source	DOI	URL	Language	Authors	Reviewers
					function in patients with chronic low back pain						
42	Excluded	Wrong study design	Garcia AN	2011	Effects of two physical therapy interventions in patients with chronic non-specific low back pain: feasibility of a randomized controlled trial	Revista Brasileira de Fisioterapia [Brazilian Journal of Physical Therapy] 2011 Sep-Oct;15(5):420-427	10.1590/s1413-35552011005000019	https://pubmed.ncbi.nlm.nih.gov/22002187/	English	Garcia AN; Gondo FLB; Costa RA; Cyrillo FN; Costa LOP	MRG, JMV
43	Excluded	Ineligible intervention: multimodal protocol	Koldas Dogan S	2008	Comparison of three different approaches in the treatment of chronic low back pain	Clinical Rheumatology 2008 Jul;27(7):873-881	10.1007/s10067-007-0815-7	https://pubmed.ncbi.nlm.nih.gov/18188660/	English	Koldas Dogan S; Sonel Tur B; Kurtais Y; Atay MB	MRG, JMV
44	Excluded	Methodological quality: PEDro score lower than 6 (PEDro < 6)	Marshall PW	2008	Muscle activation changes after exercise rehabilitation for chronic low back pain	Archives of Physical Medicine and Rehabilitation 2008 Jul;89(7):1305-1313	10.1016/j.apmr.2007.11.051	https://pubmed.ncbi.nlm.nih.gov/18586132/	English	Marshall PW; Murphy BA	MRG, JMV
45	Excluded	Wrong publication type	Kuukkanen T	2007	Effectiveness of a home exercise programme in low back pain: a randomized five-year follow-up study	Physiotherapy Research International 2007 Dec;12(4):213-224	10.1002/pri.378	https://pubmed.ncbi.nlm.nih.gov/17849435/	English	Kuukkanen T; Mlki E; Kautiainen H; Pohjolainen T	MRG, JMV
46	Excluded	Methodological quality: PEDro score lower than 6 (PEDro < 6)	Gladwell V	2006	Does a program of Pilates improve chronic non-specific low back pain?	Journal of Sport Rehabilitation 2006;15(4):338-350	10.1123/jsr.15.4.338	https://journals.humankinetics.com/view/journals/jsr/15/4/article-p338.xml	English	Gladwell V; Head S; Haggar M; Beneke R	MRG, JMV
47	Excluded	Ineligible intervention: no eligible node	Unal M	2020	Investigating the effects of myofascial induction therapy techniques on pain, function and quality of life in patients with chronic low back pain	Journal of Bodywork and Movement Therapies 2020 Oct;24(4):188-195	10.1016/j.jbmt.2020.07.014	https://pubmed.ncbi.nlm.nih.gov/33218510/	English	Unal M; Evcik E; Kocaturk M; Algun ZC	MRG, JMV
48	Excluded	Methodological quality: PEDro score lower than 6 (PEDro < 6)	Fahmy E	2019	Efficacy of spinal extension exercise program versus muscle energy technique in treatment of chronic mechanical low back pain	Egyptian Journal of Neurology, Psychiatry and Neurosurgery 2019 Dec 2;55(77):Epub	10.1186/s41983-019-0124-5	https://link.springer.com/article/10.1186/s41983-019-0124-5	English	Fahmy E; Shaker H; Ragab W; Helmy H; Gaber M	MRG, JMV
49	Excluded	Ineligible intervention: multimodal protocol	Licciardone JC	2003	Osteopathic manipulative treatment for chronic low back pain: a randomized controlled trial [with consumer summary]	Spine 2003 Jul 1;28(13):1355-1362	10.1097/01.BRS.0000067110.61471.7D	https://pubmed.ncbi.nlm.nih.gov/12838090/	English	Licciardone JC; Stoll ST; Fulda KG; Russo DP; Siu J; Winn W; Swift J Jr	MRG, JMV
50	Excluded	Secondary publication of an excluded RCT	Mannion AF	2001	Comparison of three active therapies for chronic low back pain: results of a randomized clinical trial with one-year follow-up	Rheumatology 2001 Jul;40(7):772-778	10.1093/rheumatology/40.7.772	https://pubmed.ncbi.nlm.nih.gov/11477282/	English	Mannion AF; Muntener M; Taimela S; Dvorak J	MRG, JMV
51	Excluded	Wrong population	Bendix AF	1994	Intensiv tvaerfaglig rygbehandling -- to kontrollerede, prospektive undersogelser (Intensive multidisciplinary treatment for chronic low back pain) [Danish]	Ugeskrift for Laeger 1994 Apr 18;156(16):2388-2391,2394-2395		https://cdn.mdedge.com/files/s3fs-public/issues/articles/media_7562829_ccjm63_1-0062.pdf	English	Bendix AF; Bendix T; Vaegter K; Busch E; Kirkbak S; Ostenfeld S	MRG, JMV
52	Excluded	Wrong outcome	Baskan O	2021	Effectiveness of a clinical Pilates program in women with chronic low back pain: a randomized controlled trial	The Annals of Clinical and Analytical Medicine 2021 Sep;12(Suppl 4):478-482	10.4328/ACAM.20648	https://dn720006.ca.archive.org/0/items/effectiveness-of-a-clinical-pilates-program-in-women-with-chronic-low-back-pain-/10.4328%E2%96%A102ACAM.20648.pdf	English	Baskan O; Cavlak U; Baskan E	MRG, JMV
53	Excluded	Methodological quality: PEDro score lower than 6 (PEDro < 6)	Mazloum V	2018	The effects of selective Pilates versus extension-based exercises on rehabilitation of low back pain	Journal of Bodywork and Movement Therapies 2018 Oct;22(4):999-1003	10.1016/j.jbmt.2017.09.012	https://pubmed.ncbi.nlm.nih.gov/30368347/	English	Mazloum V; Sahebozamani M; Barati A; Nakhaee N; Rabiei P	MRG, JMV
54	Excluded	Wrong publication type	Steele J	2016	A randomized controlled trial of the effects of isolated lumbar extension exercise on lumbar kinematic pattern variability during gait in chronic low back pain	PM&R 2016 Feb;8(2):105-114	10.1016/j.pmrj.2015.06.012	https://pubmed.ncbi.nlm.nih.gov/26107538/	English	Steele J; Bruce-Low S; Smith D; Jessop D; Osborne N	MRG, JMV
55	Excluded	Ineligible intervention: no eligible node	Alfuth M	2016	Chronischer lumbaler ruckenschmerz: vergleich zwischen mobilisationstraining und training der rumpfstabilisierenden muskulatur (Chronic low back pain: comparison of mobilization and core stability exercises) [German]	Der Orthopade 2016 Jul;45(7):579-590	10.1007/s00132-016-3233-1	https://pubmed.ncbi.nlm.nih.gov/26864586/	German	Alfuth M; Cornely D	MRG, JMV
56	Excluded	Methodological quality: PEDro score lower than 6 (PEDro < 6)	Kofotolis N	2016	Effects of Pilates and trunk strengthening exercises on health-related quality of life in women with chronic low back pain	Journal of Back and Musculoskeletal Rehabilitation 2016;29(4):649-659	10.3233/BMR-160665	https://pubmed.ncbi.nlm.nih.gov/26922845/	English	Kofotolis N; Kellis E; Vlachopoulos SP; Gouitas I; Theodorakis Y	MRG, JMV
57	Excluded	Wrong publication type	Ferreira PH	2010	Changes in recruitment of transversus abdominis correlate with disability in people with chronic low back pain [with consumer summary]	British Journal of Sports Medicine 2010 Dec;44(16):1166-1172	10.1136/bjsm.2009.061515	https://pubmed.ncbi.nlm.nih.gov/19474006/	English	Ferreira PH; Ferreira ML; Maher CG; Refshauge K; Herbert RD; Hodges PW	MRG, JMV
58	Excluded	Wrong outcome	Rasmussen J	2008	Manipulation does not add to the effect of extension exercises in chronic low-back pain (LBP). A randomized, controlled, double blind study	Joint, Bone, Spine 2008 Dec;75(6):708-713	10.1016/j.jbspin.2007.12.011	https://pubmed.ncbi.nlm.nih.gov/19028434/	English	Rasmussen J; Laetgaard J; Lindecrona AL; Qvistgaard E; Bliddal H	MRG, JMV
59	Excluded	No possibility of safe data extraction	Mohseni-Bandpei MA	2006	A prospective randomised controlled trial of spinal manipulation and ultrasound in the treatment of chronic low back pain [with consumer summary]	Physiotherapy 2006 Mar;92(1):34-42	10.1016/j.physio.2005.05.005	https://www.sciencedirect.com/science/article/abs/pii/S0031940605001124	English	Mohseni-Bandpei MA; Critchley J; Staunton T; Richardson B	MRG, JMV
60	Excluded	No possibility of safe data extraction	Alp A	2014	Efficacy of core-stabilization exercise and its comparison with home-based conventional exercise in low back pain patients	Turkiye Fiziksel Tip ve Rehabilitasyon Dergisi [Turkish Journal of Physical Medicine and Rehabilitation] 2014;60(Suppl 1):S36-S42	10.5152/tftrd.2014.26817	https://ftrdergisi.com/pdf/f6118de3-f656-440d-95f2-50c620149951/articles/tftrd.2014.26817/S36-S421.pdf	English	Alp A; Mengi G; Avsaroglu AH; Mert M; Sigirli D	MRG, JMV
61	Excluded	Wrong outcome	Zheng Z	2012	Therapeutic evaluation of lumbar tender point deep massage for chronic non-specific low back pain	Chung I Tsa Chih Ying Wen Pan [Journal of Traditional Chinese Medicine] 2012 Dec;32(4):534-537	10.1016/s0254-6272(13)60066-7	https://pubmed.ncbi.nlm.nih.gov/23427384/	English	Zheng Z; Wang J; Gao Q; Hou J; Ma L; Jiang C; Chen G	MRG, JMV
62	Excluded	Ineligible intervention: multimodal protocol	Haas M	2014	Dose-response and efficacy of spinal manipulation for care of chronic low back pain: a randomized controlled trial [with consumer summary]	The Spine Journal 2014 Jul;14(7):1106-1116	10.1016/j.spinee.2013.07.468	https://pubmed.ncbi.nlm.nih.gov/24139233/	English	Haas M; Vavrek D; Peterson D; Polissar N; Neradilek MB	MRG, JMV
63	Excluded	Ineligible intervention: multimodal protocol	Sahin N	2011	Effectiveness of back school for treatment of pain and functional disability in patients with chronic low back pain: a randomized controlled trial	Journal of Rehabilitation Medicine 2011 Feb;43(3):224-229	10.2340/16501977-0650.	https://pubmed.ncbi.nlm.nih.gov/21305238/	English	Sahin N; Albayrak I; Durmus B; Ugurlu H	MRG, JMV
64	Excluded	Wrong population	Quinn K	2011	Do patients with chronic low back pain benefit from attending Pilates classes after completing conventional physiotherapy treatment?	Physiotherapy Ireland 2011;32(1):5-12	10.3233/PPR-2011-3210	https://journals.sagepub.com/doi/10.3233/PPR-2011-32102	English	Quinn K; Barry S; Barry L	MRG, JMV
65	Excluded	Ineligible intervention: no eligible node	Shirado O	2010	Multicenter randomized controlled trial to evaluate the effect of home-based exercise on patients with chronic low back pain: the Japan low back pain exercise therapy study [with consumer	Spine 2010 Aug 1;35(17):E811-E819	10.1097/BRS.0b013e3181d7a4d2	https://pubmed.ncbi.nlm.nih.gov/20628332/	English	Shirado O; Doi T; Akai M; Hoshino Y; Fujino K; Hayashi K; Marui E; Iwaya T	MRG, JMV

Record No. (merged)	Decision	Exclusion criterion	First author	Year	Title	Journal/Source	DOI	URL	Language	Authors	Reviewers
					summary]						
66	Excluded	Ineligible intervention: multimodal protocol	Bronfort G	1996	Trunk exercise combined with spinal manipulative or NSAID therapy for chronic low back pain: a randomized, observer-blinded clinical trial	Journal of Manipulative and Physiological Therapeutics 1996 Nov-Dec;19(9):570-582		https://pubmed.ncbi.nlm.nih.gov/8976475/	English	Bronfort G; Goldsmith CH; Nelson CF; Boline PD; Anderson AV	MRG, JMV
67	Excluded	Duplicate record identified at full-text screening	van Dillen LR	2021	Effect of motor skill training in functional activities versus strength and flexibility exercise on function in people with chronic low back pain: a randomized clinical trial [with consumer summary]	JAMA Neurology 2021 Apr;78(4):385-395	10.1001/jamaneurol.2020.4821	https://pubmed.ncbi.nlm.nih.gov/33369625/	English	van Dillen LR; Lanier VM; Steger-May K; Wallendorf M; Norton BJ; Civello JM; Czuppon SL; Francois...	MRG, JMV
68	Excluded	Ineligible intervention: multimodal protocol	Magalhaes MO	2018	Effectiveness of graded activity versus physiotherapy in patients with chronic nonspecific low back pain: midterm follow up results of a randomized controlled trial [with consumer summary]	Brazilian Journal of Physical Therapy 2018 Jan-Feb;22(1):82-91	10.1016/j.bjpt.2017.07.002	https://pubmed.ncbi.nlm.nih.gov/28803704/	English	Magalhaes MO; Comachio J; Ferreira PH; Pappas E; Marques AP	MRG, JMV
69	Excluded	Ineligible intervention: multimodal protocol	Zafereo J	2018	Regional manual therapy and motor control exercise for chronic low back pain: a randomized clinical trial	The Journal of Manual & Manipulative Therapy 2018;26(4):193-202	10.1080/10669817.2018.1433283	https://pmc.ncbi.nlm.nih.gov/articles/PMC7734956/	English	Zafereo J; Wang-Price S; Roddey T; Brizzolara K	MRG, JMV
70	Excluded	Ineligible intervention: multimodal protocol	Kumar S	2017	Effectiveness of Ayurvedic massage (Sahacharadi Tila) in patients with chronic low back pain: a randomized controlled trial	Journal of Alternative & Complementary Medicine 2017 Feb;23(2):109-115	10.1089/acm.2015.0272	https://pubmed.ncbi.nlm.nih.gov/27704865/	English	Kumar S; Rampp T; Kessler C; Jeitler M; Dobos GJ; Ludtke R; Meier L; Michalsen A	MRG, JMV
71	Excluded	Acute/subacute pain: pain duration of less than 12 weeks	Hsieh LL	2004	A randomized controlled clinical trial for low back pain treated by acupressure and physical therapy	Preventive Medicine 2004 Jul;39(1):168-176	10.1016/j.ypmed.2004.01.036	https://www.sciencedirect.com/science/article/abs/pii/S009174350400057X	English	Hsieh LL; Kuo C; Yen M; Chen TH	MRG, JMV
72	Excluded	Acute/subacute pain: pain duration of less than 12 weeks	Andersson GB	1999	A comparison of osteopathic spinal manipulation with standard care for patients with low back pain	The New England Journal of Medicine 1999 Nov 4;341(19):1426-1431	10.1056/NEJM199911043411903	https://pubmed.ncbi.nlm.nih.gov/10547405/	English	Andersson GB; Lucente T; Davis AM; Kappler RE; Lipton JA; Leurgans S	MRG, JMV
73	Excluded	Ineligible intervention: multimodal protocol	Sturion LA	2020	Comparison between high-velocity low-amplitude manipulation and muscle energy technique on pain and trunk neuromuscular postural control in male workers with chronic low back pain: a randomised crossover trial	South African Journal of Physiotherapy 2020 Oct 26;76(a1420):Epub	10.4102/sajp.v76i1.1420	https://pubmed.ncbi.nlm.nih.gov/33241157/	English	Sturion LA; Nowotny AH; Barillec F; Barette G; Santos GK; Teixeira FA; Fernandes KP; da Silva R	MRG, JMV
74	Excluded	Ineligible intervention: no eligible node	Ali M	2019	Comparison of two mobilization techniques in management of chronic non-specific low back pain	Journal of Bodywork and Movement Therapies 2019 Oct;23(4):918-923	10.1016/j.jbmt.2019.02.020.	https://pubmed.ncbi.nlm.nih.gov/31733783/	English	Ali M; Sethi K; Noohu M	MRG, JMV
75	Excluded	Ineligible intervention: multimodal protocol	Tavares FAG	2017	Immediate effects of joint mobilization compared to sham and control intervention for pain intensity and disability in chronic low back pain patients: randomized controlled clinical trial	Revista Dor 2017 Jan-Mar;18(1):2-7	10.5935/1806-0013.20170002	https://www.scielo.br/j/rdor/a/wvsKwvgTbYK3XVkGdPp7VMz/?format=html&lang=en	English	Tavares FAG; Chaves TC; Silva ED; Guerreiro GD; Goncalves JF; de Albuquerque AAA	MRG, JMV
76	Excluded	Ineligible intervention: multimodal protocol	de Oliveira Meirelles F	2020	Osteopathic manipulation treatment versus therapeutic exercises in patients with chronic nonspecific low back pain: a randomized, controlled and double-blind study	Journal of Back and Musculoskeletal Rehabilitation 2020;33(3):367-377	10.3233/BMR-181355	https://pubmed.ncbi.nlm.nih.gov/31658037/	English	de Oliveira Meirelles F; de Oliveira Muniz Cunha JC; da Silva EB	MRG, JMV
77	Excluded	Wrong study design	Dayanir IO	2020	Comparison of three manual therapy techniques as trigger point therapy for chronic nonspecific low back pain: a randomized controlled pilot trial	Journal of Alternative & Complementary Medicine 2020 Apr;26(4):291-299	10.1089/acm.2019.0435	https://pubmed.ncbi.nlm.nih.gov/32023423/	English	Dayanir IO; Birinci T; Kaya Mutlu E; Akcetin MA; Akdemir AO	MRG, JMV
78	Excluded	Ineligible intervention: multimodal protocol	Saracoglu I	2020	The effectiveness of pain neuroscience education combined with manual therapy and home exercise for chronic low back pain: a single-blind randomized controlled trial	Physiotherapy Theory and Practice 2020 Aug 19:Epub ahead of print	10.1080/09593985.2020.1809046	https://pubmed.ncbi.nlm.nih.gov/32812478/	English	Saracoglu I; Arik MI; Afsar E; Gokpinar HH	MRG, JMV
79	Excluded	Wrong outcome	Sarker KK	2019	Effect of spinal manipulation on pain sensitivity, postural sway, and health-related quality of life among patients with non-specific chronic low back pain: a randomised control trial	Journal of Clinical and Diagnostic Research 2019 Feb;13(2):YC01-YC05	10.7860/JCDR/2019/38074.12578	https://www.jcdr.net/articles/PDF/12578/38074_CE[Ra1]_F(SL)_PF1_(AJ_KM)_PN(SL).pdf	English	Sarker KK; Sethi J; Mohanty U	MRG, JMV
80	Excluded	Wrong outcome	Klassen E	2019	Klassische massage und akupunktur bei chronischem ruckenschmerz -- randomisierte nichtunterlegenheitsstudie (Classical massage and acupuncture in chronic back pain -- non-inferiority randomised trial) [German]	Zeitschrift fur Orthopadie und Unfallchirurgie 2019 Jun;157(3):263-269	10.1055/a-0715-2332	https://pubmed.ncbi.nlm.nih.gov/30321900/	German	Klassen E; Wiebelitz KR; Beer AM	MRG, JMV
81	Excluded	Acute/subacute pain: pain duration of less than 12 weeks	Bronfort G	2011	Supervised exercise, spinal manipulation, and home exercise for chronic low back pain: a randomized clinical trial	The Spine Journal 2011 Jul;11(7):585-598	10.1016/j.spinee.2011.01.036	https://pubmed.ncbi.nlm.nih.gov/21622028/	English	Bronfort G; Maiers MJ; Evans RL; Schulz CA; Bracha Y; Svendsen KH; Grimm RH Jr; Owens EF Jr; Garv...	MRG, JMV
82	Excluded	Wrong population	Hsieh LL	2006	Treatment of low back pain by acupressure and physical therapy: randomised controlled trial [with consumer summary]	BMJ 2006 Mar 25;332(7543):696-700	10.1136/bmj.38744.672616.AE	https://pmc.ncbi.nlm.nih.gov/articles/PMC1410852/	English	Hsieh LL; Kuo CH; Lee LH; Yen AM; Chien KL; Chen TH	MRG, JMV
83	Excluded	Ineligible intervention: multimodal protocol	Niemisto L	2003	A randomized trial of combined manipulation, stabilizing exercises, and physician consultation compared to physician consultation alone for chronic low back pain [with consumer summary]	Spine 2003 Oct 1;28(19):2185-2191	10.1097/01.BRS.0000085096.62603.61	https://pubmed.ncbi.nlm.nih.gov/14520029/	English	Niemisto L; Lahtinen-Suopanki T; Rissanen P; Lindgren K; Sarna S; Hurri H	MRG, JMV
84	Excluded	Duplicate record identified at full-text screening	Natour J	2015	Pilates improves pain, function and quality of life in patients with chronic low back pain: a randomized controlled trial [with consumer summary]	Clinical Rehabilitation 2015 Jan;29(1):59-68	10.1177/0269215514538981	https://pubmed.ncbi.nlm.nih.gov/24965957/	English	Natour J; Cazotti LA; Ribeiro LH; Baptista AS; Jones A	MRG, JMV
85	Excluded	Ineligible intervention: multimodal protocol	Shnayderman I	2013	An aerobic walking programme versus muscle strengthening programme for chronic low back pain: a randomized controlled trial [with consumer summary]	Clinical Rehabilitation 2013 Mar;27(3):207-214	10.1177/0269215512453353	https://pubmed.ncbi.nlm.nih.gov/22850802/	English	Shnayderman I; Katz-Leurer M	MRG, JMV
86	Excluded	Wrong outcome	Mostagi FQRC	2015	Pilates versus general exercise effectiveness on pain and functionality in non-specific chronic low back pain subjects	Journal of Bodywork and Movement Therapies 2015 Oct;19(4):636-645	10.1016/j.jbmt.2014.11.009	https://pubmed.ncbi.nlm.nih.gov/26592221/	English	Mostagi FQRC; Dias JM; Pereira LM; Obara K; Mazuquin BF; Silva MF; Silva MAC; de Campos RR; Barre...	MRG, JMV
87	Excluded	Ineligible intervention: no eligible node	Gunay S	2014	The effect of the muscle endurance training on the chronic low back pain	Turk Fizyoterapi ve Rehabilitasyon Dergisi [Turkish Journal of Physiotherapy and Rehabilitation] 2014;25(1):28-34	10.7603/s40680-014-0004-y	https://link.springer.com/article/10.7603/s40680-014-0004-y	English	Gunay S; Yildirim Y; Karadibak D	MRG, JMV
88	Excluded	Ineligible intervention: no eligible node	Marshall PWM	2013	Pilates exercise or stationary cycling for chronic non-specific low	Spine 2013 Jul 1;38(15):E952-E959	10.1097/BRS.0b013e318297c1e	https://pubmed.ncbi.nlm.nih.gov/23615384/	English	Marshall PWM; Kennedy S;	MRG, JMV

Record No. (merged)	Decision	Exclusion criterion	First author	Year	Title	Journal/Source	DOI	URL	Language	Authors	Reviewers
					back pain: does it matter? A randomized controlled trial with 6-month follow-up [with consumer summary]		5			Brooks C; Lonsdale C	
89	Excluded	Secondary publication of an included trial — overlapping population	França FR	2012	Effects of muscular stretching and segmental stabilization on functional disability and pain in patients with chronic low back pain: a randomized, controlled trial	Journal of Manipulative and Physiological Therapeutics 2012 May;35(4):279-285	10.1016/j.jmpt.2012.04.012	https://doi.org/10.1016/j.jmpt.2012.04.012	English	França FR; Burke TN; Caffaro RR; Ramos LA; Marques AP	MRG, JMV
90	Excluded	No ≥4-week follow-up	Bhadauria EA	2017	Comparative effectiveness of lumbar stabilization, dynamic strengthening, and Pilates on chronic low back pain: randomized clinical trial	Journal of Exercise Rehabilitation 2017 Aug;13(4):477-485	10.12965/jer.1734972.486	https://pubmed.ncbi.nlm.nih.gov/29114516/	English	Bhadauria EA; Gurudut P	MRG, JMV
91	Excluded	Ineligible intervention: multimodal protocol	Verbrugghe J	2019	Exercise intensity matters in chronic nonspecific low back pain rehabilitation	Medicine and Science in Sports and Exercise 2019;51(12):2434-2442	10.1249/MSS.0000000000002078	https://pubmed.ncbi.nlm.nih.gov/31269004/	English	Verbrugghe J; Agten A; Stevens S; Hansen D; Demoulin C; Eijnde BO; Vandenabeele F; Timmermans A	MRG, JMV
92	Excluded	Acute/subacute pain: pain duration of less than 12 weeks	van der Roer N	2008	Intensive group training protocol versus guideline physiotherapy for patients with chronic low back pain: a randomised controlled trial	European Spine Journal 2008 Sep;17(9):1193-1200	10.1007/s00586-008-0718-6	https://pubmed.ncbi.nlm.nih.gov/18663487/	English	van der Roer N; van Tulder M; Barendse J; Knol D; van Mechelen W; de Vet H	MRG, JMV
93	Excluded	Wrong population	Helmhout PH	2004	Comparison of a high-intensity and a low-intensity lumbar extensor training program as minimal intervention treatment in low back pain: a randomized trial	European Spine Journal 2004 Oct;13(6):537-547	10.1007/s00586-004-0671-y	https://pubmed.ncbi.nlm.nih.gov/15095072/	English	Helmhout PH; Harts CC; Staal JB; Candel MJ; de Bie RA	MRG, JMV
94	Excluded	Methodological quality: PEDro score lower than 6 (PEDro < 6)	Raoul T	2019	Effets d'un programme de renforcement musculaire des extenseurs du rachis chez les triathletes lombalgiques chroniques. Etude controlee randomisee chez 67 sportifs (Effects of a muscle strengthening program designed for spine extensors in triathletes with chronic back pain. Randomized controlled ...	Journal de Traumatologie du Sport 2019 Sep;36(3):183-193	10.1016/j.jts.2019.07.011	https://www.sciencedirect.com/science/article/abs/pii/S0762915X19300737	French	Raoul T; Malferiot J; Barizien N; Lhuissier FJ	MRG, JMV
95	Excluded	Ineligible intervention: multimodal protocol	Durmus D	2014	How effective is a modified exercise program on its own or with back school in chronic low back pain? A randomized-controlled clinical trial	Journal of Back and Musculoskeletal Rehabilitation 2014;27(4):553-561	10.3233/BMR-140481	https://pubmed.ncbi.nlm.nih.gov/24867906/	English	Durmus D; Unal M; Kuru O	MRG, JMV
96	Excluded	Ineligible intervention: multimodal protocol	Smith D	2011	The effect of lumbar extension training with and without pelvic stabilization on lumbar strength and low back pain	Journal of Back and Musculoskeletal Rehabilitation 2011;24(4):241-249	10.3233/BMR-2011-0301	https://pubmed.ncbi.nlm.nih.gov/22142713/	English	Smith D; Bissell G; Bruce-Low S; Wakefield C	MRG, JMV
97	Excluded	No possibility of safe data extraction	Morone G	2011	Quality of life improved by multidisciplinary back school program in patients with chronic non-specific low back pain: a single blind randomized controlled trial	European Journal of Physical and Rehabilitation Medicine 2011 Dec;47(4):533-541		https://pubmed.ncbi.nlm.nih.gov/21508915/	English	Morone G; Paolucci T; Alcuri MR; Vulpiani MC; Matano A; Bureca I; Paolucci S; Saraceni VM	MRG, JMV
98	Excluded	Wrong population	Mannion AF	1999	A randomized clinical trial of three active therapies for chronic low back pain	Spine 1999 Dec 1;24(23):2435-2448	10.1097/00007632-199912010-00004	https://pubmed.ncbi.nlm.nih.gov/10626305/	English	Mannion AF; Muntener M; Taimela S; Dvorak J	MRG, JMV
99	Excluded	Wrong population	Ehsani F	2019	The effects of stabilization exercise on the thickness of lateral abdominal muscles during standing tasks in women with chronic low back pain: a randomized triple-blinded clinical trial study	Journal of Sport Rehabilitation 2020 Sep;29(7):942-951	10.1123/jsr.2019-0058	https://pubmed.ncbi.nlm.nih.gov/31821992/	English	Ehsani F; Hedayati R; Bagheri R; Jaberzadeh S	MRG, JMV
100	Excluded	Ineligible intervention: multimodal protocol	Iversen VM	2018	Resistance band training or general exercise in multidisciplinary rehabilitation of low back pain? A randomized trial [with consumer summary]	Scandinavian Journal of Medicine & Science in Sports 2018 Sep;28(9):2074-2083	10.1111/sms.13091	https://pubmed.ncbi.nlm.nih.gov/29603805/	English	Iversen VM; Vasseljen O; Mork PJ; Gismervik S; Bertheussen GF; Salvesen O; Fimland MS	MRG, JMV
101	Excluded	Methodological quality: PEDro score lower than 6 (PEDro < 6)	Albert Anand U	2014	A study to analyse the efficacy of modified Pilates based exercises and therapeutic exercises in individuals with chronic non specific low back pain: a randomized controlled trial	International Journal of Physiotherapy and Research 2014 May-Jun;2(3):525-529	ISSN 2321-1822	https://www.ijmhr.org/ijpr_articles_vol2_3/IJPR-2014-636.pdf	English	Albert Anand U; Mariet Caroline P; Arun B; Lakshmi Gomathi G	MRG, JMV
102	Excluded	Methodological quality: PEDro score lower than 6 (PEDro < 6)	Shaughnessy M	2004	A pilot study to investigate the effect of lumbar stabilisation exercise training on functional ability and quality of life in patients with chronic low back pain	International Journal of Rehabilitation Research 2004;27(4):297-301	10.1097/00004356-200412000-00007	https://pubmed.ncbi.nlm.nih.gov/15572993/	English	Shaughnessy M; Caulfield B	MRG, JMV
103	Excluded	Methodological quality: PEDro score lower than 6 (PEDro < 6)	Santaella da Fonseca Lopes de Sousa K	2009	Assessment of a biofeedback program to treat chronic low back pain	Journal of Musculoskeletal Pain 2009;17(4):369-377	10.3109/10582450903284828	https://www.tandfonline.com/doi/full/10.3109/10582450903284828	English	Santaella da Fonseca Lopes de Sousa K; Garcia Orfale A; Mara Meireles S; Roberto Leite J; Natour J	MRG, JMV
104	Excluded	Methodological quality: PEDro score lower than 6 (PEDro < 6)	Freitas CD	2008	Estudo comparativo entre exercicios com dinamometro isocinetico e bola terapeutica na lombalgia cronica de origem mecanica (Comparison between isokinetic dynamometer and therapeutic ball exercises in chronic low-back pain of mechanical origin) [Portuguese]	Fisioterapia e Pesquisa [Physical Therapy and Research] 2008 Oct-Dec;15(4):380-386	10.1590/S1809-29502008000400011	https://www.scielo.br/j/fp/a/BTPH9fWWkctdcBNrkvcFhNd/?format=html&lang=pt	Portuguese	Freitas CD; Greve JMD	MRG, JMV
105	Excluded	Methodological quality: PEDro score lower than 6 (PEDro < 6)	Kofotolis ND	2008	Sequentially allocated clinical trial of rhythmic stabilization exercises and TENS in women with chronic low back pain [with consumer summary]	Clinical Rehabilitation 2008 Feb;22(2):99-111	10.1177/0269215507080122	https://pubmed.ncbi.nlm.nih.gov/18212032/	English	Kofotolis ND; Vlachopoulos SP; Kellis E	MRG, JMV
106	Excluded	Methodological quality: PEDro score lower than 6 (PEDro < 6)	Kang J-I	2016	Effect of exhalation exercise on trunk muscle activity and Oswestry Disability Index of patients with chronic low back pain	Journal of Physical Therapy Science 2016 Jun;28(6):1738-1742	10.1589/jpts.28.1738	https://pubmed.ncbi.nlm.nih.gov/27390406/	English	Kang J-I; Jeong D-K; Choi H	MRG, JMV
107	Excluded	Methodological quality: PEDro score lower than 6 (PEDro < 6)	Lee J-S	2016	The effects of strength exercise and walking on lumbar function, pain level, and body composition in chronic back pain patients	Journal of Exercise Rehabilitation 2016 Oct;12(5):463-470	10.12965/jer.1632650.325	https://pmc.ncbi.nlm.nih.gov/articles/PMC5091063/	English	Lee J-S; Kang S-J	MRG, JMV
108	Excluded	Methodological quality: PEDro score lower than 6 (PEDro < 6)	Akodu A	2016	Comparative efficacy of core stabilization exercise and Pilates exercise on patients with non-specific chronic low back pain	Revista Romana de Kinetoterapie [Romanian Journal of Physical Therapy] 2016 Dec;22(38):13-21	10.1016/j.physio.2016.10.304	https://revrokineto.uoradea.ro/22_38/2.rovrekineto_22_38_Akodu.pdf	English	Akodu A; Okonkwo S; Akinbo S	MRG, JMV
109	Excluded	Methodological quality: PEDro score lower than 6 (PEDro < 6)	Shamsi MB	2015	Comparing core stability and traditional trunk exercise on chronic low back pain patients using three functional lumbopelvic stability tests	Physiotherapy Theory and Practice 2015;31(2):89-98	10.3109/09593985.2014.959144	https://pubmed.ncbi.nlm.nih.gov/25317504/	English	Shamsi MB; Sarrafzadeh J; Jamshidi A	MRG, JMV
110	Excluded	Methodological quality: PEDro score lower than 6 (PEDro < 6)	Kong Y-S	2015	The effects of prone bridge exercise on the Oswestry disability index and proprioception of patients with chronic low back pain	Journal of Physical Therapy Science 2015 Sep;27(9):2749-2752	10.1589/jpts.27.2749	https://pmc.ncbi.nlm.nih.gov/articles/PMC4616086/	English	Kong Y-S; Jang G-U; Park S	MRG, JMV
111	Excluded	Methodological quality: PEDro score lower than 6 (PEDro < 6)	Goel P	2010	Effects of local versus global stabilizer strengthening in chronic low back pain	Indian Journal of Physiotherapy and Occupational Therapy 2010 Jan-Mar;4(1):68-74	10.1016/j.clinbiomech.2019.12.028	https://pubmed.ncbi.nlm.nih.gov/31923778/	English	Goel P; Veqar Z; Quddus N	MRG, JMV

Record No. (merged)	Decision	Exclusion criterion	First author	Year	Title	Journal/Source	DOI	URL	Language	Authors	Reviewers
112	Excluded	Methodological quality: PEDro score lower than 6 (PEDro < 6)	Sipaviciene S	2020	Effect of different exercise programs on non-specific chronic low back pain and disability in people who perform sedentary work [with consumer summary]	Clinical Biomechanics 2020 Mar;73:17-27	10.1016/j.clinbiomech.2019.12.028	https://pubmed.ncbi.nlm.nih.gov/31923778/	English	Sipaviciene S; Kliziene I	MRG, JMV
113	Excluded	Methodological quality: PEDro score lower than 6 (PEDro < 6)	Mane NP	2019	Effect of motor control training on isolated lumbar stabilizer and core muscle training in chronic low back pain patients	Indian Journal of Public Health Research & Development 2019 Jul;10(7):37-42	10.5958/0976-5506.2019.01533.X	https://openurl.ebsco.com/EPDB%3Agcd%3A9%3A29288713/detailv2?sid=ebsco%3Aplink%3Ascholar&id=ebsco%3Agcd%3A138553143&crl=f&link_origin=www.google.com	English	Mane NP; Varadharajulu G; Shinde S	MRG, JMV
114	Excluded	Methodological quality: PEDro score lower than 6 (PEDro < 6)	Ojoawo AO	2017	Comparative effectiveness of two stabilization exercise positions on pain and functional disability of patients with low back pain	Journal of Exercise Rehabilitation 2017 Jun;13(3):363-371	https://doi.org/10.12965//jer.1734932.466	https://www.e-jer.org/journal/view.php?number=2013600394	English	Ojoawo AO; Hassan MA; Olaogun MOB; Johnson EO; Mbada CE	MRG, JMV
115	Excluded	Methodological quality: PEDro score lower than 6 (PEDro < 6)	Shamsi M	2017	Comparison of spinal stability following motor control and general exercises in nonspecific chronic low back pain patients [with consumer summary]	Clinical Biomechanics 2017 Oct;48:42-48	10.1016/j.clinbiomech.2017.07.006	https://pubmed.ncbi.nlm.nih.gov/28728077/	English	Shamsi M; Sarrafzadeh J; Jamshidi A; Arjmand N; Ghezelbash F	MRG, JMV
116	Excluded	Methodological quality: PEDro score lower than 6 (PEDro < 6)	Khandhar RY	2020	Comparative effect of trunk balance exercise over conventional back care exercise in patients with chronic mechanical low back pain	Indian Journal of Public Health Research & Development 2020 Jun;11(6):786-791	10.37506/ijphrd.v11i6.9880	https://medicopublication.com/index.php/ijphrd/article/view/9880	English	Khandhar RY; Sathya P; Paul J	MRG, JMV
117	Excluded	Methodological quality: PEDro score lower than 6 (PEDro < 6)	Johannsen F	1995	Exercises for chronic low back pain: a clinical trial	The Journal of Orthopaedic and Sports Physical Therapy 1995 Aug;22(2):52-59	10.2519/jospt.1995.22.2.52	https://pubmed.ncbi.nlm.nih.gov/7581431/	English	Johannsen F; Remvig L; Kryger P; Beck P; Warming S; Lybeck K; Dreyer V; Larsen LH	MRG, JMV
118	Excluded	Methodological quality: PEDro score lower than 6 (PEDro < 6)	Risch SV	1993	Lumbar strengthening in chronic low back pain patients. Physiologic and psychological benefits	Spine 1993 Feb;18(2):232-238		https://pubmed.ncbi.nlm.nih.gov/8185727/	English	Risch SV; Norvell NK; Pollock ML; Risch ED; Langer H; Fulton M; Graves JE; Leggett SH	MRG, JMV
119	Excluded	Methodological quality: PEDro score lower than 6 (PEDro < 6)	Cortell-Tormo JM	2018	Effects of functional resistance training on fitness and quality of life in females with chronic nonspecific low-back pain	Journal of Back and Musculoskeletal Rehabilitation 2018;31(1):95-105	10.3233/BMR-169684	https://pubmed.ncbi.nlm.nih.gov/28826168/	English	Cortell-Tormo JM; Sanchez PT; Chulvi-Medrano I; Tortosa-Martinez J; Manchado-Lopez C; Llana-Bello...	MRG, JMV
120	Excluded	Methodological quality: PEDro score lower than 6 (PEDro < 6)	Ki C	2016	The effects of forced breathing exercise on the lumbar stabilization in chronic low back pain patients	Journal of Physical Therapy Science 2016 Dec;28(12):3380-3383	10.1589/jpts.28.3380	https://pubmed.ncbi.nlm.nih.gov/28174456/	English	Ki C; Heo M; Kim H-Y; Kim E-J	MRG, JMV
121	Excluded	Methodological quality: PEDro score lower than 6 (PEDro < 6)	Guven Z	2003	Kronik mekanik bel agrili hastalarda lumbopelvik stabilizasyon egzersiz egitiminin etkinligi (Effectiveness of lumbopelvic stabilization exercise education in patients with chronic mechanical low back pain) [Turkish]	Turkiye Fiziksel Tip ve Rehabilitasyon Dergisi [Turkish Journal of Physical Medicine and Rehabilitation] 2003;49(5):12-17	10.3233/BMR-169684	https://openaccess.marmara.edu.tr/entities/publication/44933f42-08e6-459f-8857-d9d3ab8f3813	Turkish	Guven Z; Marangozoglu I; Gunduz OH	MRG, JMV
122	Excluded	Ineligible intervention: multimodal protocol	Licciardone JC	2013	Osteopathic manual treatment and ultrasound therapy for chronic low back pain: a randomized controlled trial	Annals of Family Medicine 2013 Mar-Apr;11(2):122-129	10.1370/afm.1468	https://pubmed.ncbi.nlm.nih.gov/23508598/	English	Licciardone JC; Minotti DE; Gatchel RJ; Kearns CM; Singh KP	MRG, JMV
123	Excluded	Ineligible intervention: multimodal protocol	Ahmed UA	2021	Effects of dynamic stabilization exercises and muscle energy technique on selected biopsychosocial outcomes for patients with chronic non-specific low back pain: a double-blind randomized controlled trial	Scandinavian Journal of Pain 2021;21(3):495-511	10.1515/sjpain-2020-0133	https://pubmed.ncbi.nlm.nih.gov/33641272/	English	Ahmed UA; Maharaj SS; van Oosterwijck J	MRG, JMV
124	Excluded	Methodological quality: PEDro score lower than 6 (PEDro < 6)	Kell RT	2009	A comparison of two forms of periodized exercise rehabilitation programs in the management of chronic nonspecific low-back pain [with consumer summary]	Journal of Strength & Conditioning Research 2009 Mar;23(2):513-523	10.1519/JSC.0b013e3181918a6e	https://pubmed.ncbi.nlm.nih.gov/19209082/	English	Kell RT; Asmundson GJG	MRG, JMV
125	Excluded	Ineligible intervention: no eligible node	Kim B	2020	Core Stability and Hip Exercises Improve Physical Function and Activity in Patients with Non-Specific Low Back Pain: A Randomized Controlled Trial.	The Tohoku journal of experimental medicine	10.1620/tjem.251.193	https://pubmed.ncbi.nlm.nih.gov/32669487/	English	Kim B; Yim J	MRG, JMV
126	Excluded	Methodological quality: PEDro score lower than 6 (PEDro < 6)	Zaworski K	2021	The effectiveness of manual therapy and proprioceptive neuromuscular facilitation compared to kinesiotherapy: a four-arm randomized controlled trial.	European journal of physical and rehabilitation medicine	10.23736/S1973-9087.21.06344-9	https://pubmed.ncbi.nlm.nih.gov/33650840/	English	Zaworski K; Latosiewicz R	MRG, JMV
127	Excluded	Secondary publication of an included trial — overlapping population	Cruz-Díaz D	2018	The effectiveness of 12 weeks of Pilates intervention on disability, pain and kinesiophobia in patients with chronic low back pain: a randomized controlled trial.	Clinical rehabilitation	10.1177/0269215518768393	https://pubmed.ncbi.nlm.nih.gov/29651872/	English	Cruz-Díaz D; Romeu M; Velasco-González C; Martínez-Amat A; Hita-Contreras F	MRG, JMV
128	Excluded	Methodological quality: PEDro score lower than 6 (PEDro < 6)	Inani SB	2013	Effect of core stabilization exercises versus conventional exercises on pain and functional status in patients with non-specific low back pain: a randomized clinical trial.	Journal of back and musculoskeletal rehabilitation	10.3233/BMR-2012-0348	https://pubmed.ncbi.nlm.nih.gov/23411647/	English	Inani SB; Selkar SP	MRG, JMV
129	Excluded	Methodological quality: PEDro score lower than 6 (PEDro < 6)	Borges TP	2012	Massage application for occupational low back pain in nursing staff.	Revista latino-americana de enfermagem	10.1590/s0104-11692012000300012	https://pubmed.ncbi.nlm.nih.gov/22991113/	English	Borges TP; Greve JM; Monteiro AP; da Silva RE; Giovani AM; da Silva MJ	MRG, JMV
130	Excluded	Methodological quality: PEDro score lower than 6 (PEDro < 6)	Nechvátal P	2022	Comparison of the effect of the McKenzie method and spiral stabilization in patients with low back pain: A prospective, randomized clinical trial.	Journal of back and musculoskeletal rehabilitation	10.3233/BMR-210055	https://pubmed.ncbi.nlm.nih.gov/34657873/	English	Nechvátal P; Hitrík T; Kendrová LD; Macej M	MRG, JMV
131	Excluded	Wrong publication type	Miyamoto GC	2016	Effectiveness and Cost-Effectiveness of Different Weekly Frequencies of Pilates for Chronic Low Back Pain: Randomized Controlled Trial.	Physical therapy	10.2522/ptj.20150404	https://pubmed.ncbi.nlm.nih.gov/26294680/	English	Miyamoto GC; Moura KF; Franco YR; Oliveira NT; Amaral DD; Branco AN; Silva ML; Lin C; Cabral CM	MRG, JMV
132	Excluded	Methodological quality: PEDro score lower than 6 (PEDro < 6)	Oh YJ	2020	Comparison of Effects of Abdominal Draw-In Lumbar Stabilization Exercises with and without Respiratory Resistance on Women with Low Back Pain: A Randomized Controlled Trial.	Medical science monitor : international medical journal of experimental and clinical research	10.12659/MSM.921295	https://pubmed.ncbi.nlm.nih.gov/32182226/	English	Oh YJ; Park SH; Lee MM	MRG, JMV
133	Excluded	Acute/subacute pain: pain duration of less than 12 weeks	Cherkin DC	1998	A comparison of physical therapy, chiropractic manipulation, and provision of an educational booklet for the treatment of patients with low back pain.	The New England journal of medicine	10.1056/NEJM199810083391502	https://pubmed.ncbi.nlm.nih.gov/9761803/	English	Cherkin DC; Deyo RA; Battié M; Street J; Barlow W	MRG, JMV
134	Excluded	Wrong publication type	Yang M	2014	Effectiveness of Chinese massage therapy (Tui Na) for chronic low back pain: study protocol for a randomized controlled trial.	Trials	10.1186/1745-6215-15-418	https://pubmed.ncbi.nlm.nih.gov/25352050/	English	Yang M; Feng Y; Pei H; Deng S; Wang M; Xiao X; Zheng H; Lai Z; Chen J; Li X; He X; Liang F	MRG, JMV

Record No. (merged)	Decision	Exclusion criterion	First author	Year	Title	Journal/Source	DOI	URL	Language	Authors	Reviewers
135	Excluded	Wrong publication type	Garcia AN	2015	Efficacy of the McKenzie method in patients with chronic nonspecific low back pain: a protocol of randomized placebo-controlled trial.	Physical therapy	10.2522/ptj.20140208	https://pubmed.ncbi.nlm.nih.gov/25278336/	English	Garcia AN; Costa Lda C; Hancock MJ; de Almeida MO; de Souza FS; Costa LO	MRG, JMV
136	Excluded	Methodological quality: PEDro score lower than 6 (PEDro < 6)	Patti A	2016	Pain Perception and Stabilometric Parameters in People With Chronic Low Back Pain After a Pilates Exercise Program: A Randomized Controlled Trial.	Medicine	10.1097/MD.0000000000002414	https://pubmed.ncbi.nlm.nih.gov/26765419/	English	Patti A; Bianco A; Paoli A; Messina G; Montalto MA; Bellafiore M; Battaglia G; Iovane A; Palma A	MRG, JMV
137	Excluded	Acute/subacute pain: pain duration of less than 12 weeks	Kendall KD	2015	The effect of the addition of hip strengthening exercises to a lumbopelvic exercise programme for the treatment of non-specific low back pain: A randomized controlled trial.	Journal of science and medicine in sport	10.1016/j.jsams.2014.11.006	https://pubmed.ncbi.nlm.nih.gov/25467200/	English	Kendall KD; Emery CA; Wiley JP; Ferber R	MRG, JMV
138	Excluded	Wrong population	Masharawi Y	2013	The effect of non-weight bearing group-exercising on females with non-specific chronic low back pain: a randomized single blind controlled pilot study.	Journal of back and musculoskeletal rehabilitation	10.3233/BMR-130391	https://pubmed.ncbi.nlm.nih.gov/23948819/	English	Masharawi Y; Nadaf N	MRG, JMV
139	Excluded	Acute/subacute pain: pain duration of less than 12 weeks	Helmhout PH	2008	Isolated lumbar extensor strengthening versus regular physical therapy in an army working population with nonacute low back pain: a randomized controlled trial.	Archives of physical medicine and rehabilitation	10.1016/j.apmr.2007.12.050	https://pubmed.ncbi.nlm.nih.gov/18675396/	English	Helmhout PH; Harts CC; Viechtbauer W; Staal JB; de Bie RA	MRG, JMV
140	Excluded	Methodological quality: PEDro score lower than 6 (PEDro < 6)	Kohlbeck FJ	2005	Supplemental care with medication-assisted manipulation versus spinal manipulation therapy alone for patients with chronic low back pain.	Journal of manipulative and physiological therapeutics	10.1016/j.jmpt.2005.03.003	https://pubmed.ncbi.nlm.nih.gov/15883577/	English	Kohlbeck FJ; Haldeman S; Hurwitz EL; Dagenais S	MRG, JMV
141	Excluded	Methodological quality: PEDro score lower than 6 (PEDro < 6)	Paolucci T	2012	Psychological features and outcomes of the Back School treatment in patients with chronic non-specific low back pain. A randomized controlled study.	European journal of physical and rehabilitation medicine		https://pubmed.ncbi.nlm.nih.gov/22095057/	English	Paolucci T; Morone G; Iosa M; Fusco A; Alcuri R; Matano A; Bureca I; Saraceni VM; Paolucci S	MRG, JMV
142	Excluded	Methodological quality: PEDro score lower than 6 (PEDro < 6)	Andrade SC	2008	[Back school for patients with non-specific chronic low-back pain: benefits from the association of an exercise program with patient's education].	Acta reumatologica portuguesa		https://pubmed.ncbi.nlm.nih.gov/19107089/	Portuguese	Andrade SC; Araújo AG; Vilar MJ	MRG, JMV
143	Excluded	Methodological quality: PEDro score lower than 6 (PEDro < 6)	Suh, JH	2019	The effect of lumbar stabilization and walking exercises on chronic low back pain: a randomized controlled trial	Medicine (Madr); 2019; 98(26); :e16173	10.1097/MD.0000000000016173	https://www.cochranelibrary.com/central/doi/10.1002/central/CN-01954748/full	English	Suh, JH; Kim, H; Jung, GP; Ko, JY; Ryu, JS	MRG, JMV
144	Excluded	Ineligible intervention: multimodal protocol	Frost, H	1995	Randomised controlled trial for evaluation of fitness programme for patients with chronic low back pain	BMJ; 1995; 310(6973); :151-154	10.1136/bmj.310.6973.151	https://www.cochranelibrary.com/central/doi/10.1002/central/CN-00109597/full	English	Frost, H; Klaber Moffett, JA; Moser, JS; Fairbank, JC	MRG, JMV
145	Excluded	Wrong population	Rydeard, R	2006	Pilates-based therapeutic exercise: effect on subjects with nonspecific chronic low back pain and functional disability: a randomized controlled trial	Journal of orthopaedic and sports physical therapy; 2006; 36(7); :472-484	10.2519/jospt.2006.2144	https://www.cochranelibrary.com/central/doi/10.1002/central/CN-00567007/full	English	Rydeard, R; Leger, A; Smith, D	MRG, JMV
146	Excluded	Wrong study design	Farragher, JB	2019	Effects of lumbar extensor muscle strengthening and neuromuscular control retraining on disability in patients with chronic low back pain: a protocol for a randomised controlled trial	BMJ open; 2019; 9(8); :e028259	10.1136/bmjopen-2018-028259	https://www.cochranelibrary.com/central/doi/10.1002/central/CN-01986979/full	English	Farragher, JB; Pranata, A; Williams, G; El-Ansary, D; Parry, SM; Kasza, J; Bryant, A	MRG, JMV
147	Excluded	Methodological quality: PEDro score lower than 6 (PEDro < 6)	Kliziene, I	2017	Effects of a 16-week Pilates exercises training program for isometric trunk extension and flexion strength	Journal of bodywork and movement therapies; 2017; 21(1); :124-132	10.1016/j.jbmt.2016.06.005	https://www.cochranelibrary.com/central/doi/10.1002/central/CN-01473146/full	English	Kliziene, I; Sipaviciene, S; Vilkiene, J; Astrauskiene, A; Cibulskas, G; Klizas, S; Cizauskas, G	MRG, JMV
148	Excluded	Acute/subacute pain: pain duration of less than 12 weeks	Cleland, JA	2009	Comparison of the effectiveness of three manual physical therapy techniques in a subgroup of patients with low back pain who satisfy a clinical prediction rule: a randomized clinical trial	Spine; 2009; 34(25); :2720-2729	10.1097/BRS.0b013e3181b48809	https://www.cochranelibrary.com/central/doi/10.1002/central/CN-00732899/full	English	Cleland, JA; Fritz, JM; Kulig, K; Davenport, TE; Eberhart, S; Magel, J; Childs, JD	MRG, JMV
149	Excluded	Methodological quality: PEDro score lower than 6 (PEDro < 6)	Brandt, Y	2015	A Randomized Controlled Trial of Core Strengthening Exercises in Helicopter Crewmembers with Low Back Pain	Aerospace medicine and human performance; 2015; 86(10); :889-894	10.3357/AMHP.4245.2015	https://www.cochranelibrary.com/central/doi/10.1002/central/CN-01133111/full	English	Brandt, Y; Currier, L; Plante, TW; Schubert Kabban, CM; Tvaryanas, AP	MRG, JMV
150	Excluded	Ineligible intervention: multimodal protocol	Jalalvandi, F	2022	Effects of back exercises versus transcutaneous electric nerve stimulation on relief of pain and disability in operating room nurses with chronic non-specific LBP: a randomized clinical trial	BMC musculoskeletal disorders; 2022; 23(1); :291	10.1186/s12891-022-05227-7	https://www.cochranelibrary.com/central/doi/10.1002/central/CN-02388370/full	English	Jalalvandi, F; Ghasemi, R; Mirzaei, M; Shamsi, M	MRG, JMV
151	Excluded	Ineligible intervention: heterogeneous cointervention	Ibrahim AA	2023	Effectiveness of patient education plus motor control exercise versus patient education alone versus motor control exercise alone for rural community-dwelling adults with chronic low back pain: a randomised clinical trial	BMC Musculoskeletal Disorders 2023 Feb 23;24(142):Epub	10.1186/s12891-022-06108-9	https://pmc.ncbi.nlm.nih.gov/articles/PMC9948461/	English	Ibrahim AA; Akindele MO; Ganiyu SO	MRG, JMV
152	Excluded	Ineligible intervention: no eligible node	Turci AM	2023	Self-administered stretching exercises are as effective as motor control exercises for people with chronic non-specific low back pain: a randomised trial [with consumer summary]	Journal of Physiotherapy 2023 Apr;69(2):93-99	10.1016/j.jphys.2023.02.016	https://www.sciencedirect.com/science/article/pii/S1836955323000176	English	Turci AM; Nogueira CG; Nogueira Carrer HC; Chaves TC	MRG, JMV
153	Excluded	Ineligible intervention: no eligible node	Hernandez-Lucas P	2023	Effects of a back school-based intervention on non-specific low back pain in adults: a randomized controlled trial	BMC Complementary Medicine and Therapies 2023 Jul 10;23(229):Epub	10.1186/s12906-023-04061-1	https://link.springer.com/article/10.1186/s12906-023-04061-1	English	Hernandez-Lucas P; Leiros-Rodriguez R; Mota J; Garcia-Soidan JL	MRG, JMV
154	Excluded	Ineligible intervention: heterogeneous cointervention	Rodrigues dos Santos Junior I	2023	Pilates method in low back pain: a randomized clinical trial	Fisioterapia Brasil [Physical Therapy Brazil] 2023;24(5):564-579	10.33233/fb.v24i5.5430	https://www.researchgate.net/publication/375049891_Pilates_method_in_low_back_pain_a_randomized_clinical_trial	English	Rodrigues dos Santos Junior I; de Souza Mousinho RR; Parizotto NA; Correia Coutinho CC	MRG, JMV
155	Excluded	Wrong publication type	Mirshahi M	2023	Effectiveness of a Core Stability Exercise Program on Pain and Function in Musicians with Chronic Low Back Pain: A Randomized Controlled Trial	Medical Problems of Performing Artists 2023 Dec;38(4):207-213	10.21091/mppa.2023.4025	https://europepmc.org/article/MED/38041185	English	Mirshahi M; Najafi R; Golbakhsh M; Mirshahi A; Pishkuhi MA	MRG, JMV
156	Excluded	Methodological quality: PEDro score lower than 6 (PEDro < 6)	Kumar GP	2024	A comparative evaluation of core stabilization and back school exercise programs in chronic low back pain management	Journal of Cardiovascular Disease Research 2024;15(8):1213-1217	https://doi.org/10.48047/	https://www.jcdronline.org/index.php/JCDR/article/view/10401	English	Kumar GP; Khan J; Pal R; Khan U	MRG, JMV
157	Excluded	Ineligible intervention: duplicate node	Porwal S	2023	Enhancing functional ability in chronic nonspecific lower back pain: the impact of EMG-guided trunk stabilization exercises	Healthcare 2023 Jul 28;11(15):28	10.3390/healthcare11152153	https://pmc.ncbi.nlm.nih.gov/articles/PMC10418369/	English	Porwal S; Rizvi MR; Sharma A; Ahmad F; Alshahrani MS; Raizah A; Shaik AR; Seyam MK; Miraj M; Alkh...	MRG, JMV

Record No. (merged)	Decision	Exclusion criterion	First author	Year	Title	Journal/Source	DOI	URL	Language	Authors	Reviewers
158	Excluded	Ineligible intervention: duplicate node	Gibbs MT	2022	Does a powerlifting inspired exercise programme better compliment pain education compared to bodyweight exercise for people with chronic low back pain? A multicentre, single-blind, randomised controlled trial [with consumer summary]	Clinical Rehabilitation 2022 Sep;36(9):1199-1213	10.1177/02692155221095484	https://journals.sagepub.com/doi/abs/10.1177/02692155221095484	English	Gibbs MT; Morrison NM; Raftry S; Jones MD; Marshall PW	MRG, JMV
159	Excluded	No possibility of safe data extraction	Dal C	2024	The effects of connective tissue massage and classical massage on pain, lumbar mobility, function, disability, and well-being in chronic low back pain: a three-arm randomized controlled trial [with consumer summary]	Explore 2024 Nov-Dec;20(6):103029	10.1016/j.explore.2024.103029	https://www.sciencedirect.com/science/article/abs/pii/S1550830724001368	English	Dal C; Koc M; Bayar B	MRG, JMV
160	Excluded	Ineligible intervention: duplicate node	Popovich JM	2024	The effects of osteopathic manipulative treatment on pain and disability in patients with chronic low back pain: a single-blinded randomized controlled trial	Journal of Osteopathic Medicine 2024 Jan;124(5):219-230	10.1515/jom-2022-0124	https://jom.osteopathic.org/abstract/the-effects-of-osteopathic-manipulative-treatment-on-pain-and-disability-in-patients-with-chronic-low-back-pain-a-single-blinded-randomized-controlled-trial/	English	Popovich JM; Cholewicki J; Reeves NP; DeStefano LA; Rowan JJ; Francisco TJ; Prokop LL; Zatkin MA; ...	MRG, JMV
161	Excluded	Ineligible intervention: heterogeneous cointervention	Khan K	2024	Comparison of the effects of dry needling and spinal manipulative therapy versus spinal manipulative therapy alone on functional disability and endurance in patients with nonspecific chronic low back pain: an experimental study	Medicine 2024 Sep 20;103(38):e39734	10.1097/MD.0000000000039734	https://journals.lww.com/md-journal/fulltext/2024/09200/comparison_of_the_effects_of_dry_needling_and.70.aspx	English	Khan K; Ahmad A; Mohseni Bandpei MA; Kashif M	MRG, JMV
162	Excluded	Wrong outcome	Tottoli CR	2024	Effectiveness of Pilates compared with home-based exercises in individuals with chronic non-specific low back pain: Randomised controlled trial.	Clinical rehabilitation	10.1177/02692155241277041	https://pubmed.ncbi.nlm.nih.gov/39275840/	English	Tottoli CR; Ben ÂJ; da Silva EN; Bosmans JE; van Tulder M; Carregaro RL	MRG, JMV
163	Excluded	No ≥4-week follow-up	Dar G	2025	Dry needling of the gluteus-medius muscle, combined with standard care, for chronic low back pain - a pilot randomized sham-controlled trial.	The Journal of manual & manipulative therapy	10.1080/10669817.2025.2465726	https://pubmed.ncbi.nlm.nih.gov/39954045/	English	Dar G; Goldberg A	MRG, JMV
164	Excluded	Ineligible intervention: duplicate node	Verbrugghe J	2023	High intensity training improves symptoms of central sensitization at six-month follow-up in persons with chronic nonspecific low back pain: Secondary analysis of a randomized controlled trial.	Brazilian journal of physical therapy	10.1016/j.bjpt.2023.100496	https://pubmed.ncbi.nlm.nih.gov/36963161/	English	Verbrugghe J; Agten A; Stevens S; Vandenabeele F; Roussel N; Verbunt J; Goossens N; Timmermans A	MRG, JMV
165	Excluded	Ineligible intervention: multimodal protocol	Passos MHPD	2024	Effects of an eight-week physical exercise program on low back pain and function in fruit workers: A randomized controlled trial.	Journal of back and musculoskeletal rehabilitation	10.3233/BMR-230201	https://pubmed.ncbi.nlm.nih.gov/38160342/	English	Passos MHPD; Pícon SPB; Batista GA; Nascimento VYS; Oliveira FADS; Locks F; Pitangui ACR; de Araújo RC	MRG, JMV
166	Excluded	Methodological quality: PEDro score lower than 6 (PEDro < 6)	Liu M	2025	The effect of core stability training combined with fascial release on patients with nonspecific low back pain.	Technology and health care : official journal of the European Society for Engineering and Medicine	10.3233/THC-240902	https://pubmed.ncbi.nlm.nih.gov/39177625/	English	Liu M; Huang Z; Wang X; You P; Cai X	MRG, JMV
167	Excluded	Wrong population	Abdelazeim AS	2025	Effect of segmental control program on hamstring peak torque, pain and disability in patients with non-specific low back pain: A randomized controlled trial.	Journal of bodywork and movement therapies	10.1016/j.jbmt.2025.06.014	https://pubmed.ncbi.nlm.nih.gov/40954623/	English	Abdelazeim AS; Elhafez HM; Abdullah HH; Essa SA; Zahran MR	MRG, JMV
168	Excluded	Wrong population	Elgendy MH	2026	Core stability exercises versus intermittent traction in management of nonspecific chronic low back pain: a randomized controlled trial.	European journal of applied physiology	10.1007/s00421-025-05931-x	https://pubmed.ncbi.nlm.nih.gov/40796699/	English	Elgendy MH; Khalifa MH; Kentiba E; Abdelhay MI; Elgendy OM	MRG, JMV
169	Excluded	Ineligible intervention: no eligible node	Kwok BC	2026	DMA Clinical Pilates(TM) exercises improved clinical and biomechanical outcomes for adults with nonspecific chronic low back pain: a randomized controlled trial.	Disability and rehabilitation	10.1080/09638288.2025.2563762	https://pubmed.ncbi.nlm.nih.gov/40974606/	English	Kwok BC; Lim JXL; Wong JKH; Tan JJR; Kumar K; Smith HE; Kong PW	MRG, JMV
170	Excluded	Ineligible intervention: heterogeneous cointervention	Farragher, JB	2024	Neuromuscular control and resistance training for people with chronic low back pain: a randomized controlled trial	Journal of orthopaedic and sports physical therapy	10.2519/jospt.2024.12349	https://www.cochranelibrary.com/central/doi/10.1002/central/CN-02678585/full	English	Farragher, JB; Pranata, A; Williams, GP; El-Ansary, D; Parry, SM; Clark, RA; Mentiplay, B; Kasza, J; Crofts, S; Bryant, AL	MRG, JMV
171	Excluded	Ineligible intervention: duplicate node	Ulug, N	2025	Role of Latissimus Dorsi-Thoracolumbar Fascia Complex Stretching on Pain and Pain-Related Parameters in Patients With Chronic Low Back Pain: a Randomised Clinical Trial	European journal of pain (London, England)	10.1002/ejp.70143	https://www.cochranelibrary.com/central/doi/10.1002/central/CN-02911917/full	English	Ulug, N; Kodak, SB; Kodak, MI; Aslan, SN	MRG, JMV

Note. Extracted from S3.xlsx, worksheet "Excluded articles". Values were transcribed without recalculation.

Table S4a

General Characteristics of the Included Studies

Characteristic	Value
Included studies	45 studies
Analyzed comparisons	48 comparisons
Unique study arms analyzed	93 arms
Participants analyzed	N = 3,620
Participants per study	M = 80.44; range = 24–271
Countries represented	20 countries
Publication years	2007–2026
Treatment duration	M = 6.79 weeks; range = 2–12
Weighted mean age	44.88 years (N = 3,620)
Sex distribution	Women = 60.59%; men = 39.41% (n = 3,509 with available data)
Weighted mean body mass index	27.54 kg/m² (n = 3,181 with available data)
Disability outcome measures	ODI (24 studies); RMDQ (21 studies)
PEDro scale score	M = 7.36; min = 6; max = 9 (by unique study ID); M = 7.31 by comparison
RoB 2 overall judgement	Some concerns (SC) = 29; low risk (L) = 15; high risk (H) = 4

Note. The total sample size and group distribution were calculated by retaining both treatment arms when a multiarm study contributed two analyzed intervention arms, while counting the shared comparator only once by study ID. For studies coded as multiarm but contributing only one eligible/used arm, the original treatment and comparator sample sizes were retained. Sex distribution was computed using arms with available data; sex data were unavailable for n = 111 participants. Countries were harmonized as follows: Brazil (8); Spain (5); Turkey (5); Iran (4); Australia (3); China (3); United States (3); India (2); Egypt (1); Italy (1); Kosovo (1); Netherlands (1); Nigeria (1); Norway (1); Pakistan (1); South Korea (1); Sweden (1); Switzerland (1); Thailand (1); Tunisia (1). BMI = body mass index; ODI = Oswestry Disability Index; RMDQ = Roland-Morris Disability Questionnaire; RoB 2 = revised Cochrane risk-of-bias tool for randomized trials.

Table S4b

Distribution of Intervention Nodes in the Analytical Network

Node	Operational definition	Direct contrast appearances, k	Unique arms, k	Unique participants, n
CTRL	Control, no intervention, placebo, or minimal intervention	22	20	661
STMC	Stabilization exercises with motor control	20	19	674
STR	Progressive strengthening or resistance exercise	14	13	308
GE	General exercise	10	9	434
ST	Stabilization exercises without specific motor-control feedback	10	8	196
PILATES MAT	Mat Pilates	7	6	207
MDT	McKenzie/Mechanical Diagnosis and Therapy	4	4	297
SM	Spinal manipulation or mobilization	4	4	147
PILATES APP	Equipment-based Pilates	4	3	151
STM	Soft-tissue therapy, massage, or myofascial release	3	3	184
UC	Usual care	2	2	270
BS	Back School	1	1	74
STRET	Stretching	1	1	17

Note. Direct contrast appearances were calculated from the analytical CINeMA/network dataset and therefore correspond to the structure represented in the network graph. Unique arms and unique participants were counted once per study arm; in multiarm trials, shared comparator arms were counted once for arm-level summaries but contributed to multiple direct contrasts in the network. CTRL = control; STMC = stabilization exercises with motor control; STR = strengthening/resistance exercise; GE = general exercise; ST = stabilization exercises; PILATES MAT = mat Pilates; PILATES APP = equipment-based Pilates; MDT = McKenzie/Mechanical Diagnosis and Therapy; SM = spinal manipulation/mobilization; STM = soft-tissue therapy; UC = usual care; BS = Back School; STRET = stretching.

Table S5

Characteristics of the Included Comparisons and Intervention Extraction

ID	Study (author, year)	Country	DOI/URL	Treatment code	Control code	Arm analysed	Comparator analysed	Population/type	n treatment	n comparator	Duration treatment (weeks)	Duration comparator (weeks)	Frequency/session details	Detailed intervention	Detailed comparator/control	Co-interventions / common components	Outcomes extracted in Excel
1	Huang et al., 2025	China	https://doi.org/10.1186/s12891-025-08417-1	STMC	STR	Dynamic neuromuscular stabilization (DNS)	Conventional core exercises	Adults with chronic non-specific low back pain (CLBP), 18–60 years.	30	30	4	4	4 weeks; supervised sessions; 5-min warm-up + main block + 5-min cool-down.	Dynamic neuromuscular stabilization (DNS): 4 weeks of supervised sessions. The programme included a 5-min warm-up and six DNS exercises: supine diaphragmatic breathing, dead bug, side-lying rolling, bear-crawl, high side plank and kneeling-to-sitting transfer. Dose: 2 sets of 10 repetitions per exercise with 2-min rests; progression to 2 × 16 repetitions, increased task complexity and elastic bands from weeks 3–4. Emphasis was placed on abdominal breathing, intra-abdominal pressure, sagittal alignment and agonist/antagonist co-contraction.	Conventional core exercises for CLBP with the same general structure: single-/double-leg bridge, side bridge, crunch, prone plank and bird-dog. Exercises were performed slowly with natural breathing, 2 sets of 10 repetitions, progressing to 2 × 16 repetitions and elastic bands; warm-up and cool-down were also included.	Physiotherapist education/feedback; warm-up and cool-down in both groups.	RMDQ
2	Alqhtani et al., 2024	India	https://doi.org/10.3390/jcm13020475	STMC	STR	Core-strengthening exercises (CSE)	Intensive dynamic back exercises (IDBE)	Young adults with chronic non-specific low back pain (CNLBP).	15	15	6	6	6 weeks; CSE on 3 alternate days/week; IDBE with intensive progression of repetitions.	Core-strengthening exercise (CSE) plus standard intervention. Standard intervention: hot pack for 20–30 min, active stretching and isometric exercises. CSE: isolated lumbar stabilization training with hollowing/TrA and multifidus activation, TrA–multifidus co-contraction in sitting/standing and integration into functional tasks. Tracks included floor and ball bridge, dead bug, quadruped/cross crawl, supine crunch on fitball, side-support, prone/superman on fitball and standing squat/lunge with fitball. Frequency: 3 alternate days/week for 6 weeks; progression according to tolerance.	Intensive dynamic back exercise (IDBE) plus the same standard intervention. Main exercises: prone trunk lifting with the pelvis at the edge of the table, prone leg lifting with the hips at the edge, and seated pull-to-neck with resistance. Ten repetitions with 1-min pauses; progression from 50 repetitions per exercise to 60 repetitions and up to completion of the programme; hot pack between cycles.	Both groups received hot packs, active stretching and isometric exercises.	ODI
4	Rubí-Carnacea et al., 2023	Spain	https://doi.org/10.1186/s12875-023-02140-3	STMC	CTRL	TrA preactivation re-education with PBU	Primary-care usual care	Adults with chronic low back pain in primary care.	16	19	4	4	4 weeks; individual sessions of approximately 30 min.	Re-education programme for transversus abdominis (TrA) preactivation: individual sessions of approximately 30 min with warm-up and TrA training using a Pressure Biofeedback Unit for feedback; abdominal drawing-in manoeuvre; individualized dosing at 70%–79% of maximum contraction capacity, up to 3 sets, avoiding fatigue.	Conventional primary-care management according to guidelines: education about low back symptoms, advice to stay active and use of paracetamol/NSAIDs when appropriate.	The control group received usual care; the intervention included education and PBU feedback.	RMDQ
7	Khaledi & Gheitasi, 2024	Iran	https://doi.org/10.5812/aapm-144046	ST	CTRL	Isometric core stabilization exercises (ISOM CSE)	Waitlist control / education only	Men and women with chronic non-specific low back pain (NSCLBP) for >3 months, aged 22–56 years; VAS ≥2; BMI ≤30; no physical therapy, acupuncture or other treatments in the previous 3 months.	13	14	8	8	8 weeks; 3 sessions/week; 40–60 min/session: 5–7-min warm-up, 40–50-min main block and 3–5-min cool-down. All groups received postural education and abdominal bracing instruction.	ISOM CSE: isometric core stabilization programme for 8 weeks. Exercises: abdominal hollowing, prone straight leg raise, superman, teaser, curl-up, side bridge and supine extension bridge. Each exercise was performed in 4 sets, holding the contraction for 8–15 s, with 5-s rest between sets and 1-min rest between exercises. Participants were instructed to maintain abdominal contraction/bracing and normal breathing during each repetition.	Waitlist control for 8 weeks. No active therapeutic intervention; education only via a booklet about correct posture and abdominal bracing to avoid low back pain. Telephone monitoring every 2 weeks for pain status/adherence.	Common education in all three groups: postural-hygiene booklet and light abdominal bracing (~10%–20% of maximum); initial educational session with a specialist; additional exercise booklet for active groups.	ODI
7	Khaledi & Gheitasi, 2024	Iran	https://doi.org/10.5812/aapm-144046	STR	CTRL	Isotonic core stabilization exercises (ISOT CSE)	Waitlist control / education only	Men and women with chronic non-specific low back pain (NSCLBP) for >3 months, aged 22–56 years; VAS ≥2; BMI ≤30; no physical therapy, acupuncture or other treatments in the previous 3 months.	14	14	8	8	8 weeks; 3 sessions/week; 40–60 min/session: 5–7-min warm-up, 40–50-min main block and 3–5-min cool-down. All groups received postural education and abdominal bracing instruction.	ISOT CSE: isotonic core stabilization programme for 8 weeks. Same exercise selection as ISOM: abdominal hollowing, prone straight leg raise, superman, teaser, curl-up, side bridge and supine extension bridge. Each exercise was performed in 4 sets of 6–12 repetitions with dynamic back-and-forth movements and no long contraction holds; 1-min rest between exercises.	Waitlist control for 8 weeks. No active therapeutic intervention; education only via a booklet about correct posture and abdominal bracing to avoid low back pain. Telephone monitoring every 2 weeks for pain status/adherence.	Common education in all three groups: postural-hygiene booklet and light abdominal bracing (~10%–20% of maximum); initial educational session with a specialist; additional exercise booklet for active groups.	ODI
9	Alshehre et al., 2023	USA	https://doi.org/10.26603/001c.68075	ST	GE	Spinal stabilization exercises (SSE)	General flexibility/ROM exercise	Adults with chronic low back pain.	20	20	8	8	8 weeks; 4–8 supervised sessions initially + home exercise.	Spinal stabilization exercises (SSE): 4–8 supervised physiotherapy sessions during the first 4 weeks plus home exercises; weeks 5–8 consisted of home exercise only. Focus on lumbar stability and control with home-based progression.	General exercise: flexibility and range-of-motion exercises with a comparable supervision/home schedule.	Home programme in both groups.	ODI
10	Yalfani et al., 2023	Iran	https://doi.org/10.32598/ptj.13.3.442.5	ST	CTRL	Core stability + abdominal hollowing	No intervention	Women with chronic non-specific low back pain.	15	15	8	8	8 weeks; exact frequency not visible in the	Core stability exercises combined with abdominal hollowing: 8 weeks of core exercises emphasizing abdominal hollowing/deep activation to improve postural control, proprioception and balance.	Control with no intervention.	Not described in the abstract.	ODI

ID	Study (author, year)	Country	DOI/URL	Treatment code	Control code	Arm analysed	Comparator analysed	Population/type	n treatment	n comparator	Duration treatment (weeks)	Duration comparator (weeks)	Frequency/session details	Detailed intervention	Detailed comparator/control	Co-interventions / common components	Outcomes extracted in Excel
													extraction; exact frequency was not clearly reported in the extracted study information.				
14	Güler et al., 2026	Turkey	https://doi.org/10.1186/s12891-025-09433-x	STMC	STR	Core stabilization exercises	Conventional physiotherapy/strengthening	Chronic non-specific low back pain with lumbar multifidus morphological changes.	18	18	8	8	8 weeks total: 4 supervised weeks + 4 home-based weeks.	Core stabilization exercise group: a 4-week supervised clinical phase followed by a 4-week home phase. Programme focused on neuromuscular control and activation of deep trunk musculature, especially TrA and lumbar multifidus.	Conventional physiotherapy/strengthening programme: conventional strengthening and stretching exercises, also with supervised and home phases.	No systematic pharmacological co-interventions described.	ODI
16	Coutinho et al., 2026	Brazil	https://doi.org/10.1590/1414-431X2025e14863	STRET	CTRL	Active trunk stretching + lumbar segmental stabilization	Placebo stretching + lumbar segmental stabilization	Subjects with chronic non-specific low back pain.	17	17	6	6	6 weeks; 2 sessions/week; 1 h/session; follow-up at 12 and 24 weeks.	Active trunk stretching + lumbar segmental stabilization: 1-h sessions, twice weekly for 6 weeks. Combines active trunk stretching with lumbar segmental stabilization exercises.	Placebo stretching + lumbar segmental stabilization: same segmental stabilization programme with placebo stretching.	Both groups received segmental stabilization; main contrast was active versus placebo stretching.	RMDQ
17	Sıglan et al., 2023	Turkey	https://doi.org/10.1016/j.jbmt.2022.09.029	STM	CTRL	Diaphragmatic/iliopsoas myofascial release + TPM	Sham MFR + TPM	Patients with chronic low back pain.	21	21	4	4	4 weeks; TPM 20 sessions, 5/week; MFR 12 sessions, 3/week.	Diaphragmatic and iliopsoas myofascial release + traditional physiotherapy modalities (TPM). TPM: TENS, hot pack, ultrasound and exercise. MFR: 12 sessions, 3/week; bilateral diaphragm work in costal, sternal and lumbar regions, 2 sets with 10 deep breaths/min; bilateral iliopsoas release until pulse, warmth or relaxation was perceived.	TPM + sham diaphragm/iliopsoas MFR; same contact, position and duration, but light touch only, without pressure, elevation or traction.	TENS, hot pack, ultrasound and exercise in both groups.	RMDQ
18	Wongcharoen et al., 2025	Thailand	https://doi.org/10.1016/j.jbmt.2025.09.036	STMC	CTRL	Core stabilization	Control/no active exercise	People aged 18–45 years with chronic non-specific low back pain.	15	15	6	6	6 weeks; 3 sessions/week.	Core stabilization exercise: 6 weeks, 3 sessions/week. CSE aimed at improving neuromuscular control and TrA/multifidus activation	Control according to Excel: no active intervention/CON. The trial also included a CSE-flossing technique only arm, which was not used in the Excel row.	Not described outside the intervention.	ODI
20	Xu et al., 2024	China	https://doi.org/10.1016/j.heliyon.2024.e32818	STMC	STR	Core stability training	Traditional waist strength training	Young male university students with CNLBP.	30	30	8	8	8 weeks; training performed at a fixed time and under supervision; exact frequency not visible in the extraction.	Core stability training: 8-week programme designed to improve core/lumbar stability and muscle function through stability and control exercises for the waist/lumbar region and trunk; supervised by a rehabilitation therapist.	Traditional waist/lumbar strengthening/conventional strength training.	No systematic co-interventions described.	ODI
21	Gevers-Montoro et al., 2024	Spain	https://doi.org/10.1016/j.jpain.2024.02.014	SM	CTRL	Spinal manipulative therapy (HVLA)	Placebo SMT	Patients with chronic primary low back pain (CPLBP).	49	49	4	4	4 weeks; 3 sessions/week; approximately 10 min/session.	Chiropractic spinal manipulative therapy (SMT): 12 sessions over 4 weeks, 3/week, approximately 10 min. Bilateral HVLA manipulation of the most painful vertebral segment; manual prone palpation to localize the segment; side-posture lumbar manipulation with sufficient force to produce cavitation; if cavitation did not occur, the procedure was repeated once.	Validated placebo SMT: same room/time/instructions; palpation of the painful segment and side posture, but force applied bilaterally to the gluteal region with lower velocity and no cavitation.	No active co-interventions described; placebo participants were offered SMT after the protocol.	ODI
22	Raoufi et al., 2026	Iran	https://doi.org/10.1016/j.msksp.2026.103501	STMC	STR	Core stability + cognitive dual-task training	General exercise + same dual-task training	People with chronic non-specific low back pain.	24	23	5	5	5 weeks; 16 sessions; 5-min cycling warm-up + 15-min stretching; home exercise programme on non-session days.	Core stability exercises + cognitive dual-task training (CSD): 16 sessions over 5 weeks, average 3/week; 3 sets × 10 repetitions, 30-s rest. Sessions 1–4 were single-task and taught TrA/multifidus contraction; sessions 5–16 combined progressive exercises with cognitive tasks. Progression moved from low-load isometrics to functional activities and walking with increasing speed/load.	General exercise + same dual-task training (GED): strengthening of extensors/paraspinals and abdominals, without specific TrA/multifidus cues; same progression and same cognitive tasks.	Both groups received cycling, static stretching, the same cognitive tasks (word naming, n-back, sequences, counting) and a home exercise programme.	ODI
24	Cherkin et al., 2011	USA	https://doi.org/10.1059/0003-4819-155-1-201107050-00002	STM	UC	Relaxation massage	Usual care	Adults with chronic non-specific low back pain.	136	133	10	10	10 weeks; 10 weekly sessions; first visit 75–90 min and follow-up visits 50–60 min.	Relaxation massage: massage intended to induce general relaxation, using effleurage, petrissage, circular friction, vibration, rocking/jostling and holding. Ten weekly treatments; first visit 75–90 min and later visits 50–60 min; optional recommendation of home relaxation/exercises.	Continued usual care, with no special study care.	Optional home self-care exercises recommended by the massage therapist; usual care was allowed.	RMDQ
25	Harts et al., 2008	Netherlands	https://doi.org/10.1016/s0004-9514(08)70062-x	STR	CTRL	High-intensity lumbar extensor strengthening	Waiting-list control	Male military personnel with chronic non-specific low back pain.	23	21	8	8	8 weeks; 10 sessions.	High-intensity isolated lumbar extensor strengthening: 8 weeks; first 2 weeks with 2 sessions/week and the next 6 weeks with 1 session/week (10 sessions). Modified	Waiting-list control for the first 8 weeks with no intervention for low back pain.	Physiotherapist supervision; participants were asked to stop other low-back treatments during the intervention.	RMDQ

ID	Study (author, year)	Country	DOI/URL	Treatment code	Control code	Arm analysed	Comparator analysed	Population/type	n treatment	n comparator	Duration treatment (weeks)	Duration comparator (weeks)	Frequency/session details	Detailed intervention	Detailed comparator/control	Co-interventions / common components	Outcomes extracted in Excel
														lower-back machine with pelvis/hip fixation; initial load approximately 50% of maximal isometric strength; goal 15–20 repetitions. If >20 reps were completed, load increased by 2.5 kg; if <15 reps, load decreased by 2.5 kg. Slow, controlled movement: 2-s concentric/4-s eccentric; 5-min ergometer warm-up.			
26	Cruz-Diaz et al., 2017	Spain	https://doi.org/10.1016/j.ctim.2017.06.004	PILATES APP	CTRL	Equipment-based Pilates (apparatus/reformer)	Control / no intervention / placebo / minimal intervention	Patients with chronic non-specific low back pain.	34	30	12	12	12 weeks; 2 sessions/week; approximately 50 min.	Equipment-based Pilates with apparatus/reformer: 12 weeks, 2 sessions/week, approximately 50 min; warm-up with powerhouse/drawing-in and breathing; exercises such as footwork toes, leg series, shoulder bridge, hundred, arm pull, kneeling pull back, seated rotations, camel, elephant, spine stretch/back extensions, mermaid and roll down; groups of 4 supervised by an expert physiotherapist.	Control/no treatment.	No other physical therapy treatment during the trial.	RMDQ
26	Cruz-Diaz et al., 2017	Spain	https://doi.org/10.1016/j.ctim.2017.06.004	PILATES MAT	CTRL	Pilates Mat	Control / no intervention / placebo / minimal intervention	Patients with chronic non-specific low back pain.	34	30	12	12	12 weeks; 2 sessions/week; approximately 50 min.	Mat Pilates: 12 weeks, 2 sessions/week, approximately 50 min; warm-up with powerhouse/drawing-in and breathing; mat exercises such as single/double leg stretch, criss cross, single straight leg, roll up, rolling, side kick, spine twist, rowing, pull straps, swimming, teaser, leg pull, mermaid and rolling down; groups of 4 supervised.	Control/no treatment.	No other physical therapy treatment during the trial.	RMDQ
27	Senna et al., 2011	Egypt	https://doi.org/10.1097/BRS.0b013e3181f5dfe0	SM	CTRL	Spinal manipulation therapy (non-maintained)	Sham SMT	Patients with chronic non-specific low back pain for ≥6 months.	26	37	4	4	4 weeks; 12 sessions; no maintenance care in the Excel row.	Non-maintained spinal manipulation therapy: 12 sessions over 1 month, 3/week. Supine/side-lying HVLA manipulation according to symptomatic side, with thrust over the ASIS; maximum two attempts per side. After manipulation, the pelvic tilt ROM exercise was taught.	Sham spinal manipulation for 12 sessions over 1 month with minimal forces, avoiding therapeutic areas, plus pelvic tilt ROM exercise.	Both groups received back-care instructions and pelvic tilt ROM: 10 repetitions after each session and 10 repetitions 3 times/day on non-session days.	ODI
28	Macedo et al., 2012	Australia	https://doi.org/10.2522/ptj.20110290	STMC	GE	Motor control exercises	Graded activity	Patients with chronic non-specific low back pain.	86	86	8	8	Initial 8 weeks + boosters; 14 sessions; home exercise.	Motor control exercises: 14 individualized supervised sessions (12 over 8 weeks + 2 boosters at 4 and 10 months). Retraining of spine/pelvis control and deep musculature (TrA, multifidus, pelvic floor, diaphragm), with palpation/ultrasound feedback; progression from isolated 10 × 10-s contractions to static/dynamic functional tasks while controlling posture, movement and breathing. Exercises were pain-guided.	Graded activity: individualized programme of problematic activities with daily quotas and time-contingent rather than pain-contingent progression; cognitive-behavioural principles, reinforcement of wellness behaviours, pain education and relapse plan; strength/fitness/stretching exercises according to goals.	Home exercises in both groups.	RMDQ
29	van Dillen et al., 2021	USA	https://doi.org/10.1001/jamaneurol.2020.4821	STMC	GE	Motor skill training in functional activities	Strength and flexibility exercise	People with CLBP ≥12 months, functional limitation and ≥3 activities limited by LBP.	74	75	6	6	6 weeks; 1 session/week of 1 h; boosters at 6 months were possible but did not modify outcomes.	Motor skill training (MST): six weekly 1-h sessions; supervised massed practice of patient-specific functional activities limited by LBP, chosen by the participant and guided by movement classification. Training aimed to delay/reduce pain-provoking lumbar movements, increase hip/other joint use and avoid end-range lumbar positioning; extrinsic feedback was minimized, with progression based on difficulty and problem-solving. Home programme focused on functional practice.	Strength and flexibility exercise (SFE): strengthening of all trunk muscles and flexibility of trunk/lower limbs in all planes, prescribed/progressed according to ACSM guidelines; pain did not guide prescription.	Education in both groups; progressive home programme.	ODI
30	Sengul et al., 2021	Turkey	https://doi.org/10.3233/WOR-213557	STMC	ST	Stabilization exercises	Conventional exercises	Patients with chronic non-specific low back pain.	18	19	6	6	6 weeks; 3 sessions/week; no home programme.	Stabilization exercises: 3 days/week for 6 weeks, supervised 40–60-min sessions. Neutral lumbar position, transversus abdominis and pelvic floor contraction during diaphragmatic exhalation; progression by position (supine, prone, quadruped, bridge, sitting, Swiss ball) and limb movements while maintaining neutral; 5–10 s, 10 repetitions.	Conventional exercises: cat-camel mobility, posterior pelvic tilt, double knee to chest, supine cycling, bridging and lower abdominal crunch. Strength exercises: 2–4 sets of 8–12 reps; flexibility: 4–10 reps with 10–30-s stretching; progression by pain/fatigue.	Physiotherapist supervision; no HEP.	ODI
31	Gatti et al., 2011	Italy	https://doi.org/10.2519/jospt.2011.3413	ST	STR	Trunk balance exercises + flexibility	Strengthening + flexibility	Patients with chronic low back pain.	34	45	5	5	5 weeks; 10 sessions; 60 min.	Trunk balance + flexibility: 10 group sessions over 5 weeks, 2/week, 60 min. All participants: treadmill walking 15 min + spine/lower-limb flexibility 30 min. Experimental group added 15 min of trunk balance in sitting, kneeling, quadruped and supine; positions held for 30 s–2 min, progressed by hard/soft support base, eyes closed and head/upper-limb movements; 2–3 min per exercise.	Strengthening + same flexibility: 15 min strengthening for limbs/trunk at 50% MVC (quadriceps, hamstrings, latissimus dorsi) and supine abdominal strengthening, plus the same walking and flexibility.	Both groups received treadmill walking and flexibility exercises.	RMQ
32	Michaelson et al.,	Sweden	https://doi.org/10.23	STR	STMC	High-load lifting/deadlift	Low-load motor control	Patients with	35	35	8	8	8 weeks; 12 sessions.	High-load lifting (deadlift): 12 treatments	Low-load motor control: motor control	Both groups received education	RMDQ

ID	Study (author, year)	Country	DOI/URL	Treatment code	Control code	Arm analysed	Comparator analysed	Population/type	n treatment	n comparator	Duration treatment (weeks)	Duration comparator (weeks)	Frequency/session details	Detailed intervention	Detailed comparator/control	Co-interventions / common components	Outcomes extracted in Excel
	2016		40/16501977-2091					mechanical/nociceptive low back pain for >3 months.						over 8 weeks (2/week in the first month and 1/week thereafter); deadlift with barbell, starting at 10 kg, neutral spine technique, Valsalva and abdominal bracing; individualized progression by load/repetitions/sets up to approximately 70%–85% 1RM if pain did not increase; groups of 2–6.	exercises for provocative/relieving movement patterns; 3 stages: activation of stabilizers in neutral (supine, sitting, quadruped, standing), postural correction and movement dissociation, and implementation in dynamic/functional tasks; 1–3 daily home exercises.	about pain mechanisms, fear management and movement technique.	
33	Garcia et al., 2017	Brazil	https://doi.org/10.1136/bjsports-2016-097327	MDT	CTRL	McKenzie MDT	Detuned ultrasound + detuned shortwave placebo	Patients with chronic non-specific low back pain.	74	73	5	5	5 weeks; 10 sessions.	McKenzie/MDT: 10 sessions over 5 weeks, 2/week, 30–40 min + The Back Book. MDT assessment with derangement/dysfunction/postural classification; repeated exercises or sustained postures according to directional preference (flexion, extension or lateral shift). Home exercises: 10–15 repetitions, 3–5 times/day; Treat Your Own Back education and self-management.	Placebo: detuned pulsed ultrasound for 5 min in side-lying + detuned short-wave diathermy for 25 min in supine; devices disconnected but handled as active. Also received The Back Book.	The Back Book in both groups; home-exercise adherence monitored only in MDT.	RMDQ
34	Unsgaard-Tøndel et al., 2010	Norway	https://doi.org/10.2522/ptj.20090421	ST	GE	High-load sling exercises	General exercise	Patients with chronic non-specific low back pain.	36	37	8	8	8 weeks; 1 session/week.	High-load sling exercises: 8 weekly sessions of 40 min; lumbopelvic control exercises in slings with unloading elastic bands to maintain neutral spine. Positions were challenging but pain-free and progressed by reducing support; dose adjusted according to pain/fatigue.	General exercises: general trunk/limb strengthening and stretching in groups of 2–8, 1 h/week, 10 repetitions × 3 sets on equipment; home stretching if necessary.	All participants received general information and advice to stay active.	ODI
34	Unsgaard-Tøndel et al., 2010	Norway	https://doi.org/10.2522/ptj.20090421	STMC	GE	Low-load motor control exercises	General exercise	Patients with chronic non-specific low back pain.	36	37	8	8	8 weeks; 1 session/week.	Low-load motor control exercises: 8 weekly 40-min sessions; ADIM/TrA activation with real-time ultrasound, multifidus and pelvic floor contraction, progression from supine to sitting/standing and incorporation into activities of daily living; 10 home contractions 2–3 times/day.	General exercises: general trunk/limb strengthening and stretching in groups of 2–8, 1 h/week, 10 repetitions × 3 sets on equipment; home stretching if necessary.	All participants received general information and advice to stay active.	ODI
36	Valenza et al., 2017	Spain	https://doi.org/10.1177/0269215516651978	PILATES MAT	CTRL	Pilates Mat	Advice leaflet/usual activity	Patients with chronic non-specific low back pain.	27	27	8	8	8 weeks; 2 sessions/week; 45 min.	Mat Pilates: 8 weeks, 2/week, 45 min. Introduction to core activation (TrA, pelvic floor, multifidus) during diaphragmatic exhalation. Floor exercises with a 55-cm ball on a mat: spine stretch, saw, mermaid, one-/double-leg stretch, crisscross, swan dive, swimming, spine twist, one-/double-leg kick, shoulder bridge, one-leg circle, side kick and relaxation with a roller. Three levels: basic, intermediate and advanced, individually adapted.	Control with usual activities and an advice leaflet covering postural care, physical activity, lifting, sedentary activities, beliefs and active lifestyle.	Usual medication/activity without new programmes.	RMDQ
37	França et al., 2010	Brazil	https://doi.org/10.1590/S1807-59322010001000015	STMC	STR	Segmental stabilization	Superficial strengthening	Patients with chronic non-specific low back pain.	15	15	6	6	6 weeks; 2 sessions/week; 30 min.	Segmental stabilization: 6 weeks, 2 sessions/week, 30 min. Focus on TrA and multifidus: TrA exercises in quadruped and supine with knees flexed, multifidus exercises in prone, and TrA/LM co-contraction in standing. Three sets of 15 repetitions.	Superficial strengthening: strengthening of rectus abdominis, internal/external obliques and spinal erectors: trunk flexion, trunk flexion + rotation, hip flexion in supine, trunk extension in prone. Three sets of 15 repetitions.	Participants were instructed not to perform other physical programmes or home exercises.	ODI
38	Noormohammadpour et al., 2018	Iran	https://doi.org/10.4184/asj.2018.12.3.490	STMC	CTRL	Multi-step core stability exercise	Waiting-list control	Female nurses with CLBP.	18	18	8	8	8 weeks; weekly teaching session + daily home exercise.	Multi-step core stability programme: 8 home-based weeks with a weekly supervised visit. Education about the core; ADiM and paraspinal/multifidus activation in supine/quadruped; progression to functional movements, sitting, slow limb movements on the floor or Swiss ball, and daily activities. Each exercise: 3 sets/day (morning/noon/night), 10 repetitions with 10-s hold; 2 floor exercises and 2 Swiss-ball exercises per week; progression according to tolerance.	Waiting-list control with no exercise programme during the study.	Weekly telephone contact to support adherence.	RMDQ
39	Miyamoto et al., 2018	Brazil	https://doi.org/10.1136/bjsports-2017-098825	PILATES APP	CTRL	Pilates 2x/week + advice	Booklet/advice	Patients with chronic non-specific low back pain.	74	73	6	6	6 weeks; 2 sessions/week; 1 h.	Pilates twice weekly for 6 weeks, individual 1-h sessions; mat exercises with accessories (ball, magic circle, toning ball) and apparatus exercises (Barrel, Cadillac, Chair, Reformer). First session: Pilates principles and powerhouse activation (pelvic floor, gluteus maximus, multifidus, TrA) during exhalation. Session structure: 5-min warm-up, 50-min Pilates (trunk/limb stretching and strengthening), 5-min cool-down; 1 set of 8–12 reps, approximately 60%–70% 1RM by Borg; basic/intermediate/advanced levels.	Booklet/advice group: educational booklet with recommendations on posture, movements in activities of daily living, information about LBP and spine/pelvis anatomy; no additional treatment, with Pilates offered after 12 months.	All groups received advice/booklet; usual medication was allowed and monitored; co-interventions discouraged.	RMDQ
40	Murtezani et al.,	Kosovo	https://doi.org/10.32	MDT	UC	McKenzie therapy	Electrophysical agents	Workers with work-related	134	137	4	4	4 weeks; maximum 7	McKenzie therapy: MDT assessment and	Electrophysical agents: IR lamp 15 min,	No additional exercise	ODI

ID	Study (author, year)	Country	DOI/URL	Treatment code	Control code	Arm analysed	Comparator analysed	Population/type	n treatment	n comparator	Duration treatment (weeks)	Duration comparator (weeks)	Frequency/session details	Detailed intervention	Detailed comparator/control	Co-interventions / common components	Outcomes extracted in Excel
	2015		33/BMR-140511					chronic low back pain.					McKenzie sessions; 10 EPA sessions.	classification; individualized treatment with repeated movements/specific postures, manual overpressure and/or therapist-assisted mobilization. Principle: encourage the direction that centralizes pain and avoid movements that peripheralize symptoms. Exercises 5 times/day, 10–15 repetitions; maximum 7 treatments over 4 weeks; first visit 1 h and subsequent visits 30–45 min.	continuous ultrasound 1 MHz at 1.5 W/cm² for 5 min, interferential current 3.85 kHz/100–130 Hz for 30 min; 10 sessions over 4 weeks, with no physical activity.	mentioned in the EPA group; follow-up at 2 and 3 months.	
41	Arora et al., 2012	India	https://www.primescholars.com/articles/a-single-investigator-blind-randomized-controlled-trial-comparing-mckenzieexercises-and-lumbar-stabilization-exercises-in-chronic.pdf	MDT	STMC	McKenzie + standard physiotherapy	Lumbar stabilization + standard physiotherapy	Patients with chronic low back pain.	15	15	4	4	4 weeks; 6 days/week.	McKenzie + standard physical therapy: standard therapy was hot pack 10 min + Russian current; McKenzie exercises: prone lying 5 min, prone on elbows, prone press-ups/extension in lying, sustained extension with pillows, standing extension; 3 sets × 5 repetitions progressing up to 20; 6 days/week for 4 weeks; postural/ergonomic education.	Lumbar stabilization + same standard physical therapy: pelvic tilts, abdominal drawing-in with pressure feedback, SLR, circles/squares with raised leg, half-kneeling lunge, neutral-spine control; same dosage and education.	Hot packs + Russian current in both groups; medication and other therapies were not allowed.	ODI
42	Ferreira et al., 2007	Australia	https://doi.org/10.1016/j.pain.2006.12.008	STMC	GE	Motor control exercises	General exercise	Adults with chronic non-specific low back pain.	80	80	8	8	8 weeks; up to 12 sessions.	Motor control exercise: up to 12 sessions over 8 weeks. Retraining of TrA, multifidus, diaphragm and pelvic floor; ultrasound feedback; progression to functional positions and coordination of trunk muscles in tasks tailored to the patient; daily home exercises.	General exercise: Back-to-Fitness-type group programme, 1 h, up to 12 sessions; warm-up, ten 1-min exercises, warm-down, relaxation and educational advice; strengthening/stretching of major muscle groups and cardiovascular fitness; encouraged staying active.	Cognitive-behavioural principles in both exercise groups; home exercise.	RMDQ
43	Wang et al., 2012	China	https://www.semanticscholar.org/paper/Effect-of-Core-Stability-Training-on-patients-with-Wang-Lt/596494ce353b11ef649088db1f060cc0e8ee46a5	ST	STR	Core stability training	Conventional strengthening exercise	Patients with chronic low back pain.	32	28	12	12	12 weeks; 3 sessions/week; 40 min.	Core stability training: 12 weeks, 3/week, approximately 40–45 min. 5-min warm-up; neutral alignment/posture exercises (sitting alignment, finding neutral-prone); core stability exercises such as bridge with leg lifts, bridge and double knee flex, reverse bridge; 5-min cool-down; progression of difficulty.	Conventional exercise: 5-min warm-up, 15 min abdominal strengthening (sit-up with feet fixed, single/bilateral straight leg raise), 15 min trunk extensor strengthening (prone trunk extension), 5-min cool-down; progression of load and difficulty.	Supervision by registered physiotherapists.	ODI
44	Moon et al., 2013	South Korea	https://doi.org/10.5535/arm.2013.37.1.110	STMC	STR	Lumbar stabilization exercises	Dynamic lumbar strengthening	Patients with chronic non-specific low back pain.	12	12	8	8	8 weeks; 2 sessions/week; 60 min.	Lumbar stabilization exercises: 8 weeks, 2 sessions/week, 1 h. Warm-up 15 min, cool-down 10 min. Sixteen exercises for TrA, multifidus and internal oblique; hollowing with breathing, verbal/tactile feedback, 10 repetitions, 10-s hold, 3-s pause between reps and 60-s rest between exercises; progression according to tolerance.	Dynamic lumbar strengthening exercises: 14 exercises for erector spinae and rectus abdominis, with the same dosage (10 reps, 10-s hold, rests) and session structure.	Supervision in the treatment room.	ODQ
46	Garcia et al., 2013	Brazil	https://doi.org/10.2522/ptj.20120414	MDT	BS	McKenzie method	Back School method	Patients with chronic non-specific low back pain.	74	74	4	4	4 weeks; 1 session/week; daily home exercise.	McKenzie method: four weekly 1-h sessions + daily home exercises. MDT assessment and repeated exercises/sustained postures according to directional preference/centralization; progression of forces, overpressure or mobilization when symptoms did not change; education about spine care and self-management.	Back School: four weekly 1-h sessions (first individual and three group sessions), theory and practice: anatomy/biomechanics, epidemiology, muscle function, pathophysiology, treatment; breathing, kinesthetic training, lumbar/quadriceps/hamstring stretching, abdominal strengthening, joint protection and daily home exercises.	Both groups received education and home exercise.	RMDQ
47	da Luz et al., 2014	Brazil	https://doi.org/10.2522/ptj.20130277	PILATES APP	PILATES MAT	Equipment-based Pilates	Mat Pilates	Patients with chronic non-specific low back pain.	43	43	6	6	6 weeks; 2 sessions/week; 1 h.	Equipment-based Pilates: 12 sessions over 6 weeks, 2/week, 1 h, individualized and supervised treatment; Powerhouse activation in the first session; 15–20 exercises/session, ≤10 repetitions, basic/intermediate/advanced levels. Apparatus: Cadillac, Reformer, Ladder Barrel and Step Chair with springs/pulleys.	Mat Pilates: same duration, supervision and principles, but exercises performed on a mat with Swiss ball and elastic bands; 15–20 exercises/session, adapted/progressed without compensations or pain.	No adverse events reported; no recent other therapies.	RMDQ
48	Batıbay et al., 2020	Turkey	https://doi.org/10.1016/j.jos.2020.10.026	PILATES MAT	GE	Pilates Mat	Home exercise program	Women with chronic non-specific low back pain.	30	30	8	8	8 weeks; 3 sessions/week; 60 min.	Mat Pilates: 8 weeks, 3/week, 1 h. Training in Pilates principles in supine/prone/side-lying/standing; basic/intermediate exercises adapted to each patient. Core muscles were trained and muscle thickness was measured by ultrasound.	Home exercise programme: 8 weeks, 3/week, 1 h, 3 sets × 10 reps; pelvic tilt, hamstring stretch, hip flexor/lumbar extensor stretch, bridge, abdominal strengthening, cat/camel, prone forearm lean, back extensor strengthening and crossed arm/leg lift. Illustrated booklet + diary + phone calls every 2 weeks.	Supervised Pilates; HEP with telephone follow-up.	ODI
50	Miyamoto et al., 2013	Brazil	https://doi.org/10.2522/ptj.20120190	PILATES MAT	CTRL	Modified Pilates + educational booklet	Educational booklet only	Patients with chronic non-specific low back pain.	43	43	6	6	6 weeks; 2 sessions/week; 1 h.	Modified Pilates + educational booklet: 12 individual supervised sessions over 6 weeks, 2/week, 1 h. Pilates principles (centering/powerhouse, concentration, control, precision, flow, breathing). Five	Educational booklet alone: information about spine/pelvis anatomy, low back pain and posture/movement recommendations for activities of daily living; phone calls twice weekly for 6 weeks for clarification;	Booklet in both groups; usual medication allowed.	RMDQ

ID	Study (author, year)	Country	DOI/URL	Treatment code	Control code	Arm analysed	Comparator analysed	Population/type	n treatment	n comparator	Duration treatment (weeks)	Duration comparator (weeks)	Frequency/session details	Detailed intervention	Detailed comparator/control	Co-interventions / common components	Outcomes extracted in Excel
														warm-up exercises for spine/pelvis mobility + protocol of 8 exercises for breathing with core stability, posture, abdominal and multifidus strengthening, gluteal and hip flexor/extensor/adductor/abductor strengthening and flexibility; 5–10 reps, basic/intermediate/advanced levels.	Pilates offered after follow-up.		
51	Balthazard et al., 2012	Switzerland	https://doi.org/10.1186/1471-2474-13-162	SM	CTRL	Manual therapy + active exercises	Detuned ultrasound + active exercises	Patients with chronic non-specific low back pain of 12–26 weeks.	22	20	8	8	8 sessions; 4–8 weeks; 30 min/session.	Manual therapy + active exercises: 8 sessions over 4–8 weeks. MT 5–10 min: passive accessory intervertebral movements, muscle-energy techniques for iliac dysfunction, and/or HVLA rotation-lateral flexion manipulation on a stiff segment. Then active exercises: lumbar mobility, stretching (erectors, hamstrings, iliopsoas, rectus femoris, piriformis), TrA/multifidus motor control and strengthening with a band at 60%–70% MVC.	Sham therapy + same active exercises: detuned ultrasound for 5–10 min over the painful/inflammatory area, followed by the same active exercises.	Initial assessment/education and home exercises in both groups.	ODI
52	Costa et al., 2009	Australia	https://doi.org/10.2522/ptj.20090218	STMC	CTRL	Motor control exercise	Detuned SWD/ultrasound placebo	Patients with chronic low back pain >12 weeks.	77	77	8	8	8 weeks; 12 sessions; 30 min.	Motor control exercise: twelve 30-min sessions over 8 weeks (2/week in the first month, 1/week in the second). Stage 1: independent activation of TrA/multifidus, reduction of superficial overactivity, real-time ultrasound feedback, 10 reps × 10 s. Stage 2: coordination and stability in static, dynamic and functional tasks, posture/movement control; daily home exercise and discharge session 12.	Placebo: detuned short-wave diathermy for 20 min + detuned ultrasound for 5 min over 12 sessions, with a credible clinical routine and the same contact duration.	Participants were not allowed to reveal allocation; placebo was structurally equivalent.	RMDQ
53	Natour et al., 2015	Brazil	https://doi.org/10.1177/0269215514538981	PILATES MAT	CTRL	Pilates method	NSAID only	Patients with chronic non-specific low back pain.	30	30	12	12	12 weeks; 2 sessions/week; 50 min.	Pilates method + NSAID: 50-min classes, twice weekly for 90 days (12 weeks), in groups of 3–4, delivered by a certified physical educator with 10 years of Pilates experience; pre-established Pilates protocol. Note: the main PDF text does not clearly distinguish mat versus apparatus exercises.	NSAID only: 50 mg sodium diclofenac as needed (VAS >7 cm), with no other intervention.	Both groups were allowed diclofenac; intake was recorded.	RMDQ
54	Waseem et al., 2019	Pakistan	https://doi.org/10.3233/BMR-171114	STMC	GE	Core stabilization + US/TENS	Routine physical therapy + US/TENS	Pakistani patients with chronic non-specific low back pain.	60	60	6	6	6 weeks; 1 supervised session/week + 2 home sessions/week.	Core stabilization exercises + baseline US/TENS: pressure-feedback core exercise in supine/prone, multifidus exercise, front/side plank, pelvic floor, diaphragmatic strengthening, single-leg standing on foam and tandem standing with perturbation by rapid arm movements. Supervision once/week + 2 home sessions/week.	Routine physical therapy exercises + baseline US/TENS: stretching of hamstrings, calves, hip flexors and lumbar extensors; abdominal curl-up in supine, back extensors in prone and hip extensors in prone. Supervision once/week + home sessions.	Both groups received therapeutic ultrasound 3 MHz for 10 min at 50% intensity + continuous TENS for 10 min over the lumbar region.	ODI
55	Sertpoyraz et al., 2009	Turkey	https://doi.org/10.1177/0269215508099862	STR	GE	Isokinetic trunk exercise	Standard exercise program	Patients aged 20–45 years with chronic low back pain.	20	20	3	3	3 weeks; 5 sessions/week.	Isokinetic exercise: 3 weeks, 5 days/week. Warm-up walking for 10 min; Cybex Norm training for trunk flexion/extension with 5 maximal repetitions at 60°/s and 90°/s, 3 sets, 60-s rest between sets; medical supervision and feedback/motivation.	Standard exercise: 3 weeks, 5 days/week, approximately 40 min. Exercises: passive lumbar extension, passive lumbar flexion, pelvic tilt, flexor and extensor strengthening, spinal mobilization and stretching, 10 reps once/day or 5 reps twice/day.	Both groups received education to avoid aggravating movements/postures and to take care of the back.	ODI
56	Arguisuelas et al., 2017	Spain	https://doi.org/10.1097/BRS.0000000000001897	STM	CTRL	Myofascial release	Sham myofascial release	Patients with chronic non-specific low back pain.	27	27	2	2	2 weeks; 4 sessions; 40 min.	Isolated myofascial release: 4 sessions over 2 weeks, 40 min. Techniques: longitudinal sliding of lumbar paravertebral muscles with the olecranon (3 times each side), thoracolumbar fascia release with crossed hands for 5 min, quadratus lumborum release 7 min per side with oblique pressure + gentle leg traction, psoas release with transverse sliding 15 times bilaterally.	Sham MFR: gentle hand placement over the same areas without sliding, only sufficient contact for the same duration.	Usual pharmacological treatment for LBP was maintained during the study.	RMQ
58	Bello et al., 2018	Nigeria	https://doi.org/10.12968/ijtr.2018.25.9.493	ST	GE	Lumbar stabilization (McGill)	Treadmill walking	Patients with chronic mechanical low back pain.	27	26	8	8	8 weeks; 3 sessions/week.	Lumbar stabilization exercises following McGill: 3 sessions/week for 8 weeks, 30 min. Cat-camel 6–8 reps; curl-up, side bridge and bird-dog 10 reps, holds ≤7 s, 1-min rest, progression to more difficult versions according to tolerance.	Treadmill walking exercise: modified Bruce protocol, 3 sessions/week for 8 weeks, target 65%–80% maximum heart rate; progression of speed/incline, with 5-min warm-up/cool-down.	No co-interventions described.	ODI
59	Elleuch et al., 2009	Tunisia	https://doi.org/10.1179/175361409X412601	SM	CTRL	Vertebral manipulation	Sham manipulation	Patients with chronic low back pain >6 months with segmental dysfunction/MID or paravertebral hypertonia.	50	35	4	4	4 weeks; 1 session/week; follow-up at 2 months.	Vertebral manipulation: 4 true manipulations, 1/week for 4 weeks; technique selected according to the initial examination and repeated by the same manipulator.	Sham manipulation: 4 simulated manipulations, 1/week, placing the spine on stretch without manipulative thrust.	Usual analgesic/anti-inflammatory treatments were recorded; standardization not detailed.	ODI

Note. Extracted from S5.xlsx, worksheet "Supplementary_Table". Values were transcribed without recalculation.

Table S6

Studies characteristics and Distribution of Potential Effect Modifiers Across Direct Comparisons

Direct comparison	k	n	Disability scale	Treatment duration, weeks T/C	Baseline disability, % scale T/C	Age, years T/C	Women, % T/C	BMI, kg/m² T/C	PEDro score	RoB 2 overall
MDT vs BS	1	148	RMDQ	4.0 / 4.0	47.2 / 46.2	53.7 / 54.2	78.4 / 68.9	27.7 / 27.4	8.0	L:1
MDT vs CTRL	1	147	RMDQ	5.0 / 5.0	55.3 / 59.7	57.5 / 55.5	78.4 / 74.0	28.4 / 29.7	8.0	L:1
MDT vs STMC	1	30	ODI	4.0 / 4.0	36.6 / 37.0	38.2 / 38.4	60.0 / 73.3	NR / NR	8.0	SC:1
MDT vs UC	1	271	ODI	4.0 / 4.0	38.4 / 35.8	48.8 / 47.5	25.2 / 61.5	25.7 / 26.0	8.0	H:1
Pilates APP vs CTRL	2	211	RMDQ	9.0 / 9.0	50.1 / 47.5	41.3 / 42.5	66.0 / 69.5	25.7 / 26.9	7.5 (7–8)	SC:2
Pilates APP vs Pilates MAT	2	154	RMDQ	9.0 / 9.0	44.6 / 46.2	37.2 / 40.2	68.1 / 73.4	26.9 / 27.0	7.5 (7–8)	L:1/SC:1
Pilates MAT vs CTRL	4	264	RMDQ	9.5 / 9.5	42.8 / 44.5	41.4 / 40.1	76.6 / 74.3	25.0 / 25.7	7.8 (7–8)	SC:4
Pilates MAT vs GE	1	60	ODI	8.0 / 8.0	27.9 / 30.9	49.3 / 48.4	100.0 / 100.0	25.0 / 26.3	6.0	SC:1
SM vs CTRL	4	288	ODI	5.0 / 5.0	23.5 / 23.2	43.0 / 42.4	52.1 / 49.2	24.0 / 25.0	7.2 (6–9)	L:1/SC:3
ST vs CTRL	2	57	ODI	8.0 / 8.0	30.9 / 28.5	43.0 / 39.4	76.9 / 75.0	28.9 / 27.9	6.5 (6–7)	SC:2
ST vs GE	3	166	ODI	8.0 / 8.0	29.2 / 28.5	41.5 / 41.2	49.5 / 57.5	27.2 / 26.7	7.3 (7–8)	L:1/SC:1/H:1
ST vs STR	3	166	Mixed (ODI, RMDQ)	8.3 / 8.3	27.5 / 26.9	46.0 / 43.3	56.1 / 55.8	23.9 / 22.9	7.3 (6–9)	L:2/SC:1
STRET vs CTRL	1	34	RMDQ	6.0 / 6.0	33.8 / 31.9	24.5 / 27.4	76.5 / 88.2	22.5 / 28.2	9.0	L:1
STM vs CTRL	2	96	RMDQ	3.0 / 3.0	56.1 / 56.3	42.3 / 42.6	51.1 / 62.5	26.1 / 26.1	8.5 (8–9)	L:2
STM vs UC	1	269	RMDQ	10.0 / 10.0	48.3 / 43.8	47.0 / 48.0	65.0 / 62.0	28.0 / 29.0	8.0	SC:1
STMC vs CTRL	4	255	Mixed (ODI, RMDQ)	6.5 / 6.5	34.8 / 36.5	43.2 / 42.4	73.1 / 69.0	24.2 / 24.9	7.0 (6–9)	L:1/SC:2/H:1
STMC vs GE	5	674	Mixed (ODI, RMDQ)	7.2 / 7.2	42.8 / 43.3	46.1 / 45.7	63.0 / 55.3	26.3 / 26.2	7.2 (6–8)	L:2/SC:2/H:1
STMC vs ST	2	109	ODI	7.0 / 7.0	13.4 / 16.7	40.9 / 43.4	62.5 / 47.8	24.9 / 25.9	6.5 (6–7)	SC:1/H:1
STMC vs STR	8	357	Mixed (ODI, RMDQ)	6.6 / 6.6	30.7 / 30.8	33.3 / 33.7	46.5 / 48.5	24.4 / 24.4	6.9 (6–8)	SC:8
STR vs CTRL	2	72	Mixed (ODI, RMDQ)	8.0 / 8.0	20.6 / 21.7	39.4 / 38.9	21.4 / 25.0	25.5 / 28.0	7.0 (6–8)	SC:2
STR vs GE	1	40	ODI	3.0 / 3.0	16.6 / 18.8	38.8 / 38.2	80.0 / 75.0	NR / NR	6.0	L:1

Note. Values are reported as treatment/comparator (T/C) means within each direct comparison. Baseline disability was expressed as percentage of the corresponding scale range to improve comparability across ODI and RMDQ. ODI values were treated as 0-100 scores; RMDQ/RMQ values were transformed to a 0-100 metric using the formula RMDQ/24 x 100. For comparisons with more than one study, age, sex, BMI, treatment duration and baseline disability are reported as mean values across the direct comparisons. In multiarm trials, all pairwise contrasts represented in the analytical network were included; therefore, participant counts are contrast-level counts and should not be summed across rows. The BMI values recorded for Cruz-Diaz et al. were excluded from BMI summaries because they corresponded to body weight rather than BMI. Dose, supervision, progression, adherence and home-practice characteristics are reported separately in Table S8 because these variables were heterogeneously described across trials and could not be consistently summarised quantitatively.

Abbreviations. APP = apparatus-based Pilates; BMI = body mass index; BS = back school; CTRL = control; GE = general exercise; H = high risk of bias; L = low risk of bias; MAT = mat Pilates; MDT = McKenzie/Mechanical Diagnosis and Therapy; NR = not reported; ODI = Oswestry Disability Index; RMDQ = Roland-Morris Disability Questionnaire; RoB 2 = revised Cochrane risk-of-bias tool for randomised trials; SC = some concerns; SM = spinal manipulation; ST = stabilisation exercises; STMC = stabilisation exercises with motor control; STM = soft tissue manipulation; STR = strengthening or resistance exercise; STRET = stretching; T/C = treatment/comparator; UC = usual care.

Table S7

Direct-Indirect Decomposition of Network Comparisons With Both Direct and Indirect Evidence

Comparison	k	prop	nma	direct	indirect	Diff	z	p-value
MDT vs CTRL	1	0.49	-0.14	-0.15	-0.13	-0.01	-0.04	0.964
PILATES APP vs CTRL	2	0.69	-0.88	-0.9	-0.84	-0.06	-0.19	0.851
PILATES MAT vs CTRL	4	0.76	-0.64	-0.55	-0.94	0.39	1.57	0.116
ST vs CTRL	2	0.35	-0.76	-0.83	-0.72	-0.11	-0.45	0.651
STM vs CTRL	2	0.82	-0.68	-0.7	-0.59	-0.11	-0.26	0.793
STMC vs CTRL	4	0.50	-0.86	-0.85	-0.86	0	0.02	0.987
STR vs CTRL	2	0.33	-0.31	-0.48	-0.23	-0.25	-1.07	0.283
GE vs PILATES MAT	1	0.33	0.25	0.6	0.08	0.52	1.73	0.084
GE vs ST	3	0.54	0.37	0.31	0.43	-0.12	-0.58	0.561
GE vs STMC	5	0.68	0.46	0.44	0.52	-0.08	-0.45	0.652
GE vs STR	1	0.16	-0.08	-0.16	-0.06	-0.1	-0.36	0.719
MDT vs STMC	1	0.40	0.72	0.78	0.68	0.1	0.27	0.787
MDT vs UC	1	0.69	-0.33	-0.37	-0.26	-0.11	-0.26	0.793
PILATES APP vs PILATES MAT	2	0.69	-0.24	-0.21	-0.29	0.07	0.24	0.81
ST vs STMC	2	0.32	0.1	0.12	0.09	0.04	0.18	0.857
ST vs STR	3	0.49	-0.45	-0.47	-0.43	-0.04	-0.2	0.841
STM vs UC	1	0.69	-0.88	-0.84	-0.95	0.11	0.26	0.793
STMC vs STR	8	0.69	-0.54	-0.55	-0.54	-0.01	-0.06	0.954

Note. *This table reports node-splitting/direct-indirect decomposition estimates only for comparisons for which both direct evidence and an indirect pathway were available. Direct-only comparisons without an alternative indirect pathway, such as SM vs CTRL, STRET vs CTRL, and MDT vs BS, were not decomposable and are therefore not shown in this table. k = number of direct studies; prop = proportion of direct evidence; NMA = network meta-analysis; Diff = difference between the direct and indirect estimates; z = test statistic.*

Table S8

Intervention-Program Characteristics Reported Across the Analyzed Comparisons

ID	Study and comparison	Duration (wk)	Frequency/session dose	Supervision	Progression	Protocol fidelity/adherence	Home practice
1	Huang et al., 2025. STMC vs STR: Dynamic neuromuscular stabilization (DNS) vs Conventional core exercises.	T/C: 4/4	supervised sessions; 5-min warm-up + main block + 5-min cool-down.	Supervised sessions.	Yes: repetitions progressed to 2 × 16 with added task complexity and elastic bands.	Physiotherapist education/feedback reported.	Not reported.
2	Alqhtani et al., 2024. STMC vs STR: Core-strengthening exercises (CSE) vs Intensive dynamic back exercises (IDBE).	T/C: 6/6	CSE on 3 alternate days/week; IDBE with intensive progression of repetitions.	Not reported in extraction.	Yes: CSE progressed according to tolerance; IDBE progressed from 50 to 60 repetitions.	Not reported in extraction.	Not reported.
4	Rubí-Carnacea et al., 2023. STMC vs CTRL: TrA preactivation re-education with PBU vs Primary-care usual care.	T/C: 4/4	individual sessions of approximately 30 min.	Individual sessions with pressure-biofeedback guidance.	Individualized dosing at 70%–79% of maximum contraction capacity, up to 3 sets.	Pressure Biofeedback Unit feedback and education reported.	Not reported.
7	Khaledi & Gheitasi, 2024. ST vs CTRL: Isometric core stabilization exercises (ISOM CSE) vs Waitlist control / education only.	T/C: 8/8	3 sessions/week; 40–60 min/session: 5–7-min warm-up, 40–50-min main block and 3–5-min cool-down. All groups received postural education and abdominal bracing instruction.	Not reported in extraction.	Not reported in extraction.	Telephone monitoring every 2 weeks for pain/adherence.	Education booklet; exercise booklet for active groups.
7	Khaledi & Gheitasi, 2024. STR vs CTRL: Isotonic core stabilization exercises (ISOT CSE) vs Waitlist control / education only.	T/C: 8/8	3 sessions/week; 40–60 min/session: 5–7-min warm-up, 40–50-min main block and 3–5-min cool-down. All groups received postural education and abdominal bracing instruction.	Not reported in extraction.	Yes: progressed according to tolerance	Telephone monitoring every 2 weeks for pain/adherence.	Education booklet; exercise booklet for active groups.
9	Alshehre et al., 2023. ST vs GE: Spinal stabilization exercises (SSE) vs General flexibility/ROM exercise.	T/C: 8/8	4–8 supervised sessions initially + home exercise.	Mixed delivery: 4–8 supervised sessions followed by home exercise.	Home-based progression reported.	Not reported in extraction.	Yes: home programme in both groups; weeks 5–8 home-only in the stabilization arm.
10	Yalfani et al., 2023. ST vs CTRL: Core stability + abdominal hollowing vs No intervention.	T/C: 8/8	exact frequency not visible in the extraction; exact frequency was not clearly reported in the extracted study information.	Not reported in extraction.	Not reported in extraction.	Not reported in extraction.	Not reported.
14	Güler et al., 2026. STMC vs STR: Core stabilization exercises vs Conventional physiotherapy/strengthening.	T/C: 8/8	4 supervised weeks + 4 home-based weeks.	Mixed delivery: 4 supervised weeks followed by 4 home-based weeks.	Yes: progressed according to tolerance	Not reported in extraction.	Yes: 4-week home phase in both groups.
16	Coutinho et al., 2026. STRET vs CTRL: Active trunk stretching + lumbar segmental stabilization vs Placebo stretching + lumbar segmental stabilization.	T/C: 6/6	2 sessions/week; 1 h/session; follow-up at 12 and 24 weeks.	Not reported in extraction.	Not reported in extraction.	Follow-up at 12 and 24 weeks reported.	Not reported.
17	Sıglan et al., 2023. STM vs CTRL: Diaphragmatic/iliopsoas myofascial release + TPM vs Sham MFR + TPM.	T/C: 4/4	TPM 20 sessions, 5/week; MFR 12 sessions, 3/week.	Therapist-delivered MFR/TPM sessions; sham contact matched.	Not reported in extraction.	Sham MFR matched contact, position, and duration.	Not reported.
18	Wongcharoen et al., 2025. STMC vs CTRL: Core stabilization vs Control/no active exercise.	T/C: 6/6	3 sessions/week.	Not reported in extraction.	Not reported in extraction.	Not reported in extraction.	Not reported.
20	Xu et al., 2024. STMC vs STR: Core stability training vs Traditional waist strength training.	T/C: 8/8	training performed at a fixed time and under supervision; exact frequency not visible in the extraction.	Training performed at a fixed time and supervised by a rehabilitation therapist.	Not reported in extraction.	Fixed-time supervised training reported.	Not reported.
21	Gevers-Montoro et al., 2024. SM vs CTRL: Spinal manipulative therapy (HVLA) vs Placebo SMT.	T/C: 4/4	3 sessions/week; approximately 10 min/session.	Chiropractic SMT sessions with matched placebo procedures.	Not reported in extraction.	Placebo matched room, time, and instructions; SMT offered after protocol.	Not reported.
22	Raoufi et al., 2026. STMC vs STR: Core stability + cognitive dual-task training vs General exercise + same dual-task training.	T/C: 5/5	16 sessions; 5-min cycling warm-up + 15-min stretching; home exercise programme on non-session days.	Session-based programme with home exercises on non-session days.	Yes: sessions 1–4 single-task; sessions 5–16 combined progressive exercises with cognitive tasks; moved from low-load isometrics to functional/walking tasks.	Same cognitive tasks and home exercise programme across groups.	Yes: home exercise programme on non-session days in both groups.
24	Cherkin et al., 2011. STM vs UC: Relaxation massage vs Usual care.	T/C: 10/10	10 weekly sessions; first visit 75–90 min and follow-up visits 50–60 min.	Massage delivered in weekly treatment sessions.	Not reported in extraction.	Not reported in extraction.	Optional home relaxation/exercises recommended by therapist.
25	Harts et al., 2008. STR vs CTRL: High-intensity lumbar extensor strengthening vs Waiting-list control.	T/C: 8/8	10 sessions.	Physiotherapist supervision reported.	Yes: load adjusted by 2.5 kg according to completed repetitions.	Participants were asked to stop other low-back treatments.	Not reported.
26	Cruz-Díaz et al., 2017. PILATES APP vs CTRL: Equipment-based Pilates (apparatus/reformer) vs Control / no intervention / placebo / minimal intervention.	T/C: 12/12	2 sessions/week; approximately 50 min.	Supervised groups of four led by an expert physiotherapist.	Not reported in extraction.	Expert physiotherapist; no other physical therapy during trial.	Not reported.
26	Cruz-Díaz et al., 2017. PILATES MAT vs CTRL: Pilates Mat vs Control / no intervention / placebo / minimal intervention.	T/C: 12/12	2 sessions/week; approximately 50 min.	Supervised groups of four.	Not reported in extraction.	No other physical therapy during trial.	Not reported.
27	Senna et al., 2011. SM vs CTRL: Spinal manipulation therapy (non-maintained) vs Sham SMT.	T/C: 4/4	12 sessions; no maintenance care in the Excel row.	Therapist-delivered SMT/sham sessions.	Not reported in extraction.	Sham manipulation used minimal forces and avoided therapeutic areas.	Pelvic-tilt ROM exercise after sessions and on non-session days in both groups.
28	Macedo et al., 2012. STMC vs GE: Motor control exercises vs Graded activity.	T/C: 8/8	Initial 8 weeks + boosters; 14 sessions; home exercise.	Individualized supervised sessions plus home exercise.	Yes: isolated contractions progressed to static/dynamic functional tasks; pain-guided.	Palpation/ultrasound feedback reported.	Yes: home exercises in both groups.
29	van Dillen et al., 2021. STMC vs GE: Motor skill training in functional activities vs Strength and flexibility exercise.	T/C: 6/6	1 session/week of 1 h; boosters at 6 months were possible but did not modify outcomes.	Supervised weekly sessions plus home programme.	Yes: progression based on difficulty/problem-solving; SFE progressed according to ACSM guidelines.	Extrinsic feedback was minimized in MST.	Yes: progressive home programme.

ID	Study and comparison	Duration (wk)	Frequency/session dose	Supervision	Progression	Protocol fidelity/adherence	Home practice
30	Sengul et al., 2021. STMC vs ST: Stabilization exercises vs Conventional exercises.	T/C: 6/6	3 sessions/week; no home programme.	Supervised 40–60-min sessions.	Yes: progressed by body position and limb movements; comparator progressed by pain/fatigue.	Physiotherapist supervision reported.	No HEP reported.
31	Gatti et al., 2011. ST vs STR: Trunk balance exercises + flexibility vs Strengthening + flexibility.	T/C: 5/5	10 sessions; 60 min.	Group sessions.	Yes: progressed by support base, eyes closed, and head/upper-limb movements.	Not reported in extraction.	Not reported.
32	Michaelson et al., 2016. STR vs STMC: High-load lifting/deadlift vs Low-load motor control.	T/C: 8/8	12 sessions.	Group format reported for high-load lifting; low-load arm included daily home exercises.	Yes: individualized progression by load/repetitions/sets up to 70%–85% 1RM if pain did not increase.	Education on pain mechanisms, fear management, and movement technique provided.	Yes: 1–3 daily home exercises in the low-load motor-control arm.
33	Garcia et al., 2017. MDT vs CTRL: McKenzie MDT vs Detuned ultrasound + detuned shortwave placebo.	T/C: 5/5	10 sessions.	Clinic-based MDT sessions plus home exercises; placebo clinic sessions.	Yes: repeated exercises/sustained postures according to directional preference.	Home-exercise adherence monitored only in MDT.	Yes: MDT home exercises 3–5 times/day.
34	Unsgaard-Tøndel et al., 2010. ST vs GE: High-load sling exercises vs General exercise.	T/C: 8/8	1 session/week.	Group/sling and general exercise sessions; supervision not otherwise specified.	Yes: sling support reduced; dose adjusted according to pain/fatigue.	Not reported in extraction.	Home stretching if necessary in general-exercise arm.
34	Unsgaard-Tøndel et al., 2010. STMC vs GE: Low-load motor control exercises vs General exercise.	T/C: 8/8	1 session/week.	Low-load motor-control sessions with real-time ultrasound; general exercise in groups.	Yes: progressed from supine to sitting/standing and activities of daily living.	Real-time ultrasound feedback reported.	Yes: 10 home contractions 2–3 times/day in motor-control arm; home stretching if necessary in comparator.
36	Valenza et al., 2017. PILATES MAT vs CTRL: Pilates Mat vs Advice leaflet/usual activity.	T/C: 8/8	2 sessions/week; 45 min.	Not reported in extraction.	Yes: basic, intermediate, and advanced levels individually adapted.	Not reported in extraction.	Advice leaflet/usual activities; no structured exercise HEP reported.
37	França et al., 2010. STMC vs STR: Segmental stabilization vs Superficial strengthening.	T/C: 6/6	2 sessions/week; 30 min.	Not reported in extraction.	Not reported in extraction.	Other physical programmes and home exercises discouraged.	No additional home exercises.
38	Noormohammadpour et al., 2018. STMC vs CTRL: Multi-step core stability exercise vs Waiting-list control.	T/C: 8/8	weekly teaching session + daily home exercise.	Home-based programme with weekly supervised visit.	Yes: progressed to functional movements, sitting, Swiss-ball/floor tasks, and daily activities.	Weekly telephone contact to support adherence.	Yes: daily home exercise.
39	Miyamoto et al., 2018. PILATES APP vs CTRL: Pilates 2x/week + advice vs Booklet/advice.	T/C: 6/6	2 sessions/week; 1 h.	Individual Pilates sessions; advice/booklet comparator.	Yes: basic/intermediate/advanced levels; 60%–70% 1RM by Borg.	Usual medication monitored; co-interventions discouraged; Pilates offered after 12 months.	Booklet/advice in comparator; no additional treatment.
40	Murtezani et al., 2015. MDT vs UC: McKenzie therapy vs Electrophysical agents.	T/C: 4/4	maximum 7 McKenzie sessions; 10 EPA sessions.	Individualized MDT sessions; EPA sessions in comparator.	Yes: repeated movements/postures, manual overpressure, and mobilization guided by centralization/peripheralization.	Not reported in extraction.	Yes: MDT exercises 5 times/day.
41	Arora et al., 2012. MDT vs STMC: McKenzie + standard physiotherapy vs Lumbar stabilization + standard physiotherapy.	T/C: 4/4	6 days/week.	Not reported in extraction.	Yes: repetitions progressed from 3 × 5 up to 20.	Pressure feedback in stabilization arm; medication/other therapies not allowed.	Not reported.
42	Ferreira et al., 2007. STMC vs GE: Motor control exercises vs General exercise.	T/C: 8/8	up to 12 sessions.	Up to 12 treatment sessions; supervision not otherwise specified.	Yes: progressed to functional positions and task-specific trunk coordination.	Ultrasound feedback reported.	Yes: daily home exercises/home exercise.
43	Wang et al., 2012. ST vs STR: Core stability training vs Conventional strengthening exercise.	T/C: 12/12	3 sessions/week; 40 min.	Supervision by registered physiotherapists.	Yes: difficulty progressed in ST; load/difficulty progressed in STR.	Registered physiotherapist supervision reported.	Not reported.
44	Moon et al., 2013. STMC vs STR: Lumbar stabilization exercises vs Dynamic lumbar strengthening.	T/C: 8/8	2 sessions/week; 60 min.	Supervision in the treatment room.	Yes: progressed according to tolerance.	Verbal/tactile feedback reported.	Not reported.
46	Garcia et al., 2013. MDT vs BS: McKenzie method vs Back School method.	T/C: 4/4	1 session/week; daily home exercise.	Four weekly 1-h sessions; Back School included individual and group sessions.	Yes: force progression, overpressure, or mobilization if symptoms did not change.	Not reported in extraction.	Yes: daily home exercises in both groups.
47	da Luz et al., 2014. PILATES APP vs PILATES MAT: Equipment-based Pilates vs Mat Pilates.	T/C: 6/6	2 sessions/week; 1 h.	Individualized and supervised Pilates treatment in both arms.	Yes: basic/intermediate/advanced levels; exercises adapted/progressed without compensation or pain.	No adverse events; no recent other therapies reported.	Not reported.
48	Batıbay et al., 2020. PILATES MAT vs GE: Pilates Mat vs Home exercise program.	T/C: 8/8	3 sessions/week; 60 min.	Supervised Pilates; comparator was home exercise programme with telephone follow-up.	Yes: Pilates exercises adapted to each patient.	Comparator diary and phone calls every 2 weeks.	Yes: comparator home exercise programme.
50	Miyamoto et al., 2013. PILATES MAT vs CTRL: Modified Pilates + educational booklet vs Educational booklet only.	T/C: 6/6	2 sessions/week; 1 h.	Individual supervised Pilates sessions; educational-booklet comparator.	Yes: 5–10 repetitions; basic/intermediate/advanced levels.	Protocol of 8 Pilates exercises; phone calls twice weekly for clarification in comparator.	Educational booklet; no structured exercise HEP reported.
51	Balthazard et al., 2012. SM vs CTRL: Manual therapy + active exercises vs Detuned ultrasound + active exercises.	T/C: 8/8	8 sessions; 4–8 weeks; 30 min/session.	Manual therapy/active-exercise sessions.	Not reported in extraction.	Not reported in extraction.	Yes: initial assessment/education and home exercises in both groups.
52	Costa et al., 2009. STMC vs CTRL: Motor control exercise vs Detuned SWD/ultrasound placebo.	T/C: 8/8	12 sessions; 30 min.	Clinic sessions with contact-matched placebo.	Yes: Stage 1 isolated activation progressed to Stage 2 static/dynamic/functional tasks.	Real-time ultrasound feedback; credible placebo with same contact duration.	Yes: daily home exercise in motor-control arm.
53	Natour et al., 2015. PILATES MAT vs CTRL: Pilates method vs NSAID only.	T/C: 12/12	2 sessions/week; 50 min.	Group classes delivered by a certified physical educator.	Not reported in extraction.	Pre-established Pilates protocol; diclofenac intake recorded.	Not reported.
54	Waseem et al., 2019. STMC vs GE: Core stabilization + US/TENS vs Routine physical therapy + US/TENS.	T/C: 6/6	1 supervised session/week + 2 home sessions/week.	Mixed delivery: one supervised session/week plus two home sessions/week.	Not reported in extraction.	Pressure-feedback exercise reported.	Yes: two home sessions/week in both groups.
55	Sertpoyraz et al., 2009. STR vs GE: Isokinetic trunk exercise vs Standard exercise program.	T/C: 3/3	5 sessions/week.	Medical supervision and feedback/motivation.	Yes: progressed according to tolerance.	Medical supervision and feedback/motivation reported.	Not reported.
56	Arguisuelas et al., 2017. STM vs CTRL: Myofascial release vs Sham myofascial release.	T/C: 2/2	4 sessions; 40 min.	Therapist-delivered MFR/sham sessions.	Not reported in extraction.	Sham contact matched over the same areas and duration.	Not reported.
58	Bello et al., 2018. ST vs GE: Lumbar stabilization (McGill) vs Treadmill walking.	T/C: 8/8	3 sessions/week.	Not reported in extraction.	Yes: stabilization progressed to more difficult versions according to tolerance; treadmill progressed by speed/incline.	Not reported in extraction.	Not reported.
59	Elleuch et al., 2009. SM vs CTRL: Vertebral manipulation vs Sham manipulation.	T/C: 4/4	1 session/week; follow-up at 2 months.	Manipulation repeated by the same manipulator; sham manipulation matched session schedule.	Not reported in extraction.	Same manipulator; analgesic/anti-inflammatory treatments recorded.	Not reported.

Note. T/C = treatment/comparator; wk = weeks; HEP = home exercise programme; ACSM = American College of Sports Medicine; EPA = electrophysical agents; MDT = McKenzie/Mechanical Diagnosis and Therapy; MFR = myofascial release; MST = motor skill training; ROM = range of motion; SMT = spinal manipulative therapy; TPM = traditional physiotherapy modalities.

Table S9

Comparison between the registered PROSPERO protocol and the final review methods

Methodological item	Registered PROSPERO protocol	Final review method	Nature of the modification or clarification	Timing of the decision	Rationale	Potential impact on the review
**Review outcome**	Pain and function/disability outcomes were included in the protocol.	The present manuscript focused exclusively on self-reported disability at the end of the intervention, assessed using the Roland-Morris Disability Questionnaire or the Oswestry Disability Index. Studies reporting pain outcomes only, without eligible ODI or RMDQ disability data, were excluded from the present disability-focused review as wrong outcome. Pain outcomes were not analysed or interpreted in this manuscript.	Restriction of the scope and eligibility criteria for the present disability-focused report.	Defined before the final eligibility set for the disability-focused NMA was locked and before quantitative synthesis of disability outcomes.	To provide a focused analysis of a clinically central outcome and avoid combining pain and disability as different outcome domains within the same network.	This restriction limits the conclusions to post-treatment disability assessed with ODI or RMDQ. Trials reporting pain only, for example VAS without eligible disability data, were not included in this manuscript. Therefore, no conclusions can be drawn regarding pain outcomes or medium-/long-term outcomes. The decision was not based on the direction, magnitude, or statistical significance of treatment effects.
**Intervention nodes**	The protocol prespecified therapeutic exercise and manual therapy categories, including strengthening/resistance exercise, stabilisation exercises, Pilates, McKenzie method, spinal manipulation, soft tissue manipulation and dry needling.	Operational refinements were introduced: stabilisation exercise was separated from stabilisation with an explicit motor control component; Mat Pilates was separated from equipment-based Pilates; general exercise, back school, stretching and usual care were retained as accessory connectivity nodes when they were required to preserve network structure.	Refinement of the nodal classification.	Performed during the intervention classification process and before quantitative network synthesis, based on intervention descriptions rather than treatment-effect estimates.	To improve clinical interpretability, reduce inappropriate pooling of clinically different interventions and make the classification reproducible.	Greater nodal specificity may improve clinical interpretation but can reduce node size and increase reliance on indirect evidence. These implications were considered in the interpretation and CINeMA assessment.
**Dry needling node**	Dry needling was listed among the manual therapy interventions of interest.	Dry needling was not included in the final network because no eligible RCT met the inclusion criteria for this node.	Consequence of eligibility assessment; not a post-hoc analytical modification.	Determined after eligibility assessment and before network synthesis.	NMA can only include interventions represented by eligible studies.	This reduced the representation of manual therapy techniques in the network, but did not involve a selective analytical decision based on treatment effects.
**Statistical model**	A fixed-effect model was specified in the protocol.	A frequentist random-effects NMA was used.	Change in the statistical model.	Adopted after completion of data extraction and before any quantitative network analysis, model fitting, treatment-effect estimation, ranking analysis, or CINeMA confidence adjudication. The decision was based on the clinical and methodological variability identified across the eligible trials, not on the direction, magnitude, or statistical significance of treatment effects.	The eligible trials differed in intervention content, treatment duration, dose, supervision, comparator characteristics and disability instruments. A random-effects model was therefore considered more appropriate than a fixed-effect model for the expected between-study variability.	Random-effects modelling accounts for expected between-study heterogeneity and generally provides more conservative uncertainty estimates than fixed-effect modelling. This choice did not modify study eligibility, intervention nodes, extracted disability data, or the structure of the network. The subsequently observed heterogeneity reinforced the need for cautious interpretation of pooled estimates and rankings, but it was not the basis for selecting the model.
**PEDro eligibility threshold and RoB 2 assessment**	Methodological quality assessment was planned. The protocol included exclusion of RCTs with PEDro score <6.	RoB 2 was used for domain-level risk-of-bias assessment. The PEDro <6 criterion was retained only as an eligibility threshold and was not used to weight studies, rank interventions or determine certainty of evidence.	Clarification of a protocol-specified eligibility criterion and its distinction from domain-level risk-of-bias assessment.	The PEDro threshold was protocol-specified and applied before quantitative synthesis. RoB 2 assessment was conducted before CINeMA confidence adjudication.	PEDro was retained to respect the registered eligibility criteria, while RoB 2 was used to assess domain-specific bias more appropriately.	Excluding studies using a summary methodological score may introduce selection bias. This was acknowledged as a limitation. RoB 2 judgments informed interpretation and CINeMA within-study bias assessment.
**Confidence in network estimates**	CINeMA was not specified in the original PROSPERO protocol.	Confidence in network estimates for post-treatment disability was assessed using CINeMA.	Additional certainty assessment.	Added after protocol registration as a methodological refinement and before final certainty interpretation of the disability network estimates.	CINeMA is a GRADE-based framework specifically developed for assessing confidence in NMA estimates and improves transparency of certainty judgments.	CINeMA did not modify eligibility criteria, intervention nodes, statistical model or effect estimates. It affected only the interpretation of confidence in the network estimates.

Methodological item	Registered PROSPERO protocol	Final review method	Nature of the modification or clarification	Timing of the decision	Rationale	Potential impact on the review
**Decision threshold for CINeMA**	No SMD decision threshold was specified in the original protocol.	A +/-0.20 SMD decision threshold was applied uniformly to CINeMA judgments for imprecision, heterogeneity and incoherence.	Addition of an interpretative decision threshold.	Defined after the decision to use CINeMA and before applying threshold-dependent CINeMA domain judgments for imprecision, heterogeneity and incoherence, and before final confidence adjudication.	Because disability was synthesized using SMDs across ODI and RMDQ, a single instrument-specific MCID could not be applied consistently. The +/-0.20 SMD threshold was used as a conservative boundary for a small standardized effect.	The threshold influenced CINeMA judgments and confidence ratings. It should not be interpreted as an ODI- or RMDQ-specific MCID. Confidence ratings are therefore conservative and threshold-dependent.

Note. The modifications or clarifications listed in this table were not made on the basis of the direction, magnitude, or statistical significance of the treatment effects. Operational decisions regarding node classification were made before quantitative network synthesis. CINeMA was added as an interpretative framework before final certainty interpretation of the disability network estimates. The ±0.20 SMD threshold was then defined before applying threshold-dependent CINeMA judgments and before final confidence adjudication. Neither decision modified study eligibility, extracted disability data, intervention nodes, the statistical model, effect estimates, or the structure of the disability network.

Abbreviations: CINeMA, Confidence in Network Meta-Analysis; MCID, minimal clinically important difference; NMA, network meta-analysis; ODI, Oswestry Disability Index; PROSPERO, International Prospective Register of Systematic Reviews; RCT, randomised controlled trial; RMDQ, Roland-Morris Disability Questionnaire; RoB 2, revised Cochrane risk-of-bias tool for randomised trials; SMD, standardised mean difference; VAS, Visual Analogue Scale.

Figures

Figure S1

Risk-of-bias assessment of included intervention comparisons for disability outcomes (Cochrane Risk of Bias 2 tool)

Intention-to-treat

Unique ID	Study ID	Experimental	Comparator	Outcome	Weigh	D1	D2	D3	D4	D5	Overall
1	Huang	Stabilisation/Motor Cc	Strengthening	Disability	1	+	+	+	+	!	!
2	Alqhtani	Stabilisation/Motor Cc	Strengthening	Disability	1	+	+	+	+	!	!
4	Rubí-Carnacea	Stabilisation/Motor Cc	Control	Disability	1	+	+	+	!	+	!
7	Khaledi	Strengthening	Control	Disability	1	+	!	!	!	!	!
7	Khaledi	Strengthening	Control	Disability	1	+	!	!	!	!	!
9	Alshehre	Stabilisation	General Exercise	Disability	1	!	+	+	+	+	!
10	Yalfani	Stabilisation	Control	Disability	1	+	+	+	!	!	!
14	Güler	Stabilisation/Motor Cc	Strengthening	Disability	1	!	+	!	!	+	!
16	Coutinho	Stretching	Control	Disability	1	+	+	+	+	+	+
17	Siglan	Soft Tissue Manipulat	Control	Disability	1	+	+	+	+	+	+
18	Wongcharoen	Stabilisation/Motor Cc	Control	Disability	1	!	+	+	!	!	!
20	Xu	Stabilisation/Motor Cc	Strengthening	Disability	1	!	+	+	+	!	!
21	Gevers-Montorc	Lumbar Spinal Manipu	Control	Disability	1	+	+	+	+	+	+
22	Raoufi	Stabilisation/Motor Cc	Strengthening	Disability	1	!	+	+	+	+	!
24	Cherkin	Soft Tissue Manipulat	Control	Disability	1	+	+	+	!	+	!
25	Harts	Strengthening	Control	Disability	1	+	+	!	!	+	!
26	Cruz-Díaz	Pilates	Control	Disability	1	+	+	+	!	+	!
26	Cruz-Díaz	Pilates	Control	Disability	1	+	+	+	!	+	!
27	Senna	Lumbar Spinal Manipu	Control	Disability	1	+	!	+	!	!	!
28	Macedo	Stabilisation/Motor Cc	General Exercise	Disability	1	+	+	!	+	+	!
29	van Dillen	Stabilisation/Motor Cc	General Exercise	Disability	1	+	+	+	+	+	+
30	Sengul	Stabilisation/Motor Cc	Strengthening	Disability	1	!	+	+	+	!	!
31	Gatti	Stabilisation	Strengthening	Disability	1	+	+	+	+	+	+
32	Michaelson	Strengthening	Stabilisation/Motor Cor	Disability	1	+	+	!	+	+	!
33	Garcia	McKenzie	Control	Disability	1	+	+	+	+	+	+
34	Unsgaard-Tønd	Stabilisation/Motor Cc	General Exercise	Disability	1	+	+	-	+	+	-
34	Unsgaard-Tønd	Stabilisation/Motor Cc	General Exercise	Disability	1	+	+	-	+	+	-
36	Valenza	Pilates	Control	Disability	1	!	+	+	!	+	!
37	França	Stabilisation/Motor Cc	Strengthening	Disability	1	+	+	+	+	+	+
38	Noormohammac	Stabilisation/Motor Cc	Control	Disability	1	+	!	-	!	+	-
39	Miyamoto	Pilates Reformer	Control	Disability	1	+	+	+	!	+	!
40	Murtezani	McKenzie	Usual Care	Disability	1	+	!	-	!	!	-
41	Arora	McKenzie	Stabilisation/Motor Cor	Disability	1	!	+	+	+	+	!
42	Ferreira	Stabilisation/Motor Cc	General Exercise	Disability	1	+	+	!	!	!	!
43	Wang	Stabilisation	Strengthening	Disability	1	+	+	+	+	+	+
44	Moon	Stabilisation/Motor Cc	Strengthening	Disability	1	!	+	!	+	!	!
46	Garcia	McKenzie	Back School	Disability	1	+	+	+	+	+	+
47	da Luz	Pilates Reformer	Pilates	Disability	1	+	+	+	+	+	+
48	Batıbay	Pilates	General Exercise	Disability	1	!	+	!	+	+	!
50	Miyamoto	Pilates	Control	Disability	1	+	+	+	!	+	!
51	Balthazard	Lumbar Spinal Manipu	Control	Disability	1	+	+	!	!	!	!
52	Costa	Stabilisation/Motor Cc	Control	Disability	1	+	+	+	+	+	+
53	Natour	Pilates	Control	Disability	1	+	+	+	!	+	!
54	Waseem	Stabilisation/Motor Cc	General Exercise	Disability	1	+	+	+	+	+	+
55	Sertpoyraz	Strengthening	General Exercise	Disability	1	+	+	+	+	+	+
56	Arguisuelas	Soft Tissue Manipulat	Control	Disability	1	+	+	+	+	+	+
58	Bello	Stabilisation	General Exercise	Disability	1	+	+	+	+	+	+
59	Elleuch	Lumbar Spinal Manipu	Control	Disability	1	!	+	+	+	+	!

+ Low risk
! Some concerns
- High risk

D1 Randomisation process
D2 Deviations from the intended interventions
D3 Missing outcome data
D4 Measurement of the outcome
D5 Selection of the reported result

Note. Traffic-light plot showing domain-level and overall risk-of-bias judgements for each included comparison. D1 = randomisation process; D2 = deviations from the intended interventions; D3 = missing outcome data; D4 = measurement of the outcome; D5 = selection of the reported result. Green indicates low risk of bias, yellow indicates some concerns, and red indicates high risk of bias.

Figure S2

Direct and Indirect Proportions, Minimum Parallelism Values, and Minimum Trajectory Values for Possible Network Comparisons

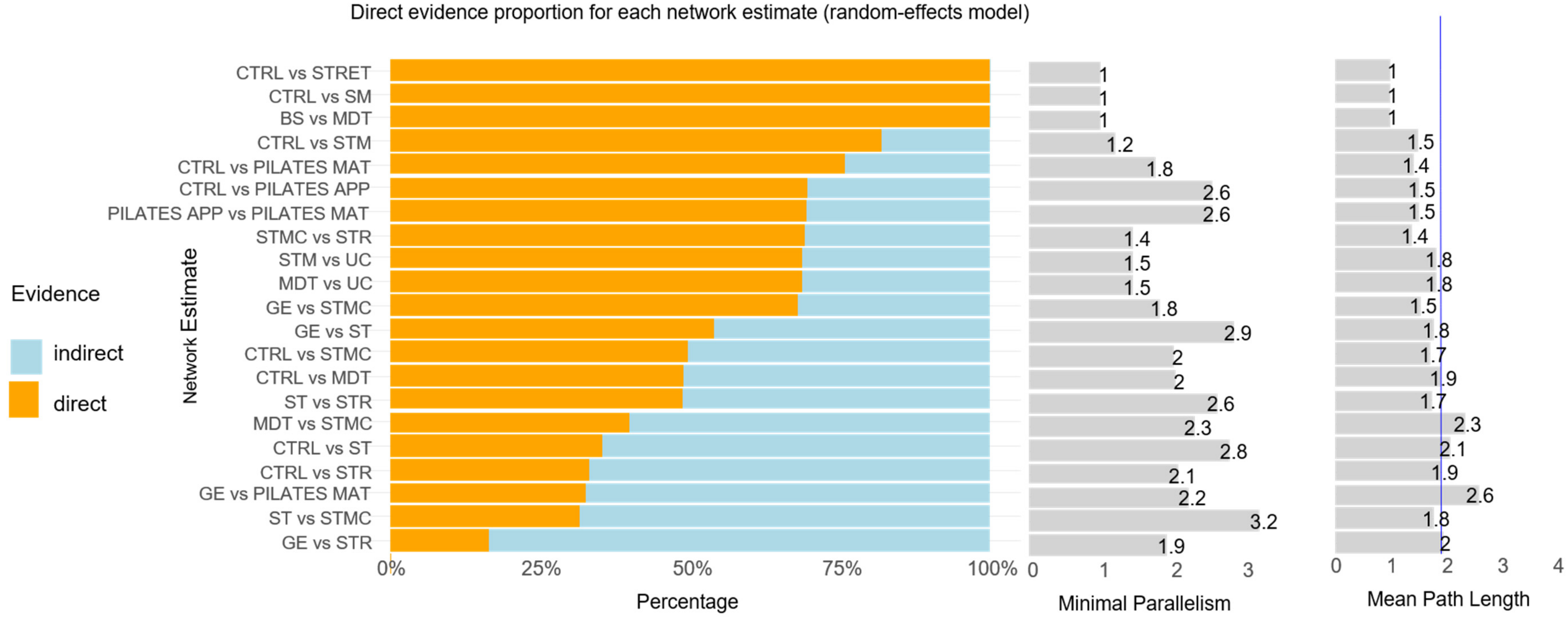

Figure S3

Comparison-Adjusted Funnel Plot and Egger Test

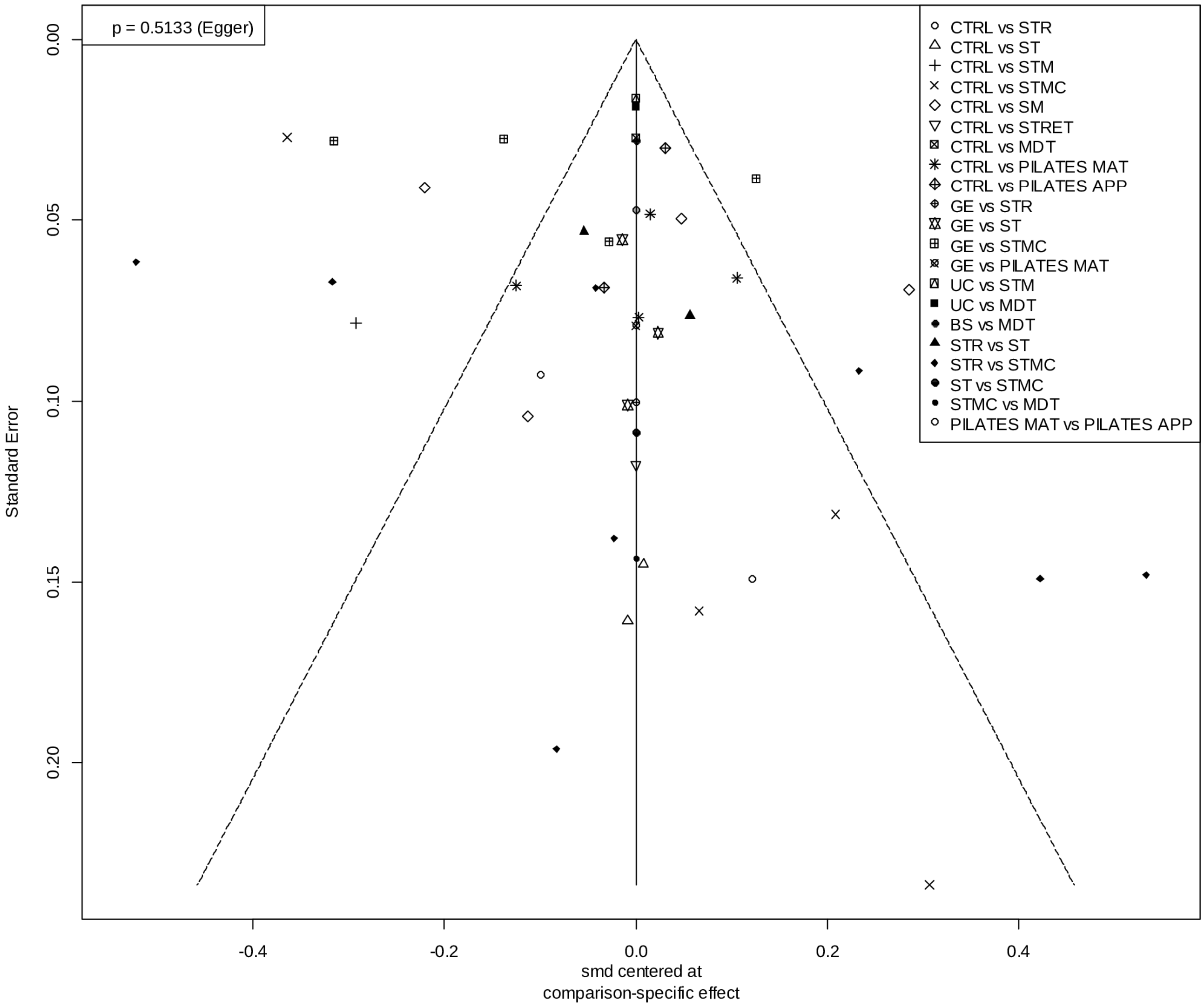

Supplementary Text S1

CINeMA confidence assessment and conservative final adjudication criteria

Purpose. This supplementary text documents the criteria used to review automatic CINeMA domain-level judgments and assign final confidence ratings for the post-treatment disability network meta-analysis.

Protocol status. The CINeMA assessment and the ±0.20 SMD decision threshold were not specified in the original PROSPERO protocol. They were introduced after registration, before final interpretation of the disability NMA, as a methodological refinement to improve transparency. The assessment did not modify eligibility criteria, intervention nodes, the statistical model, or network effect estimates.

Scope. The criteria apply only to the 12 intervention-versus-CTRL comparisons for post-treatment self-reported disability in the final random-effects SMD network.

General principles

Principle	Operational meaning
Automatic output as starting point	Automatic CINeMA domain-level judgments were retained unless additional objective network diagnostics indicated concerns not fully captured by the automatic output.
Downgrade-only adjudication	Manual adjudication could only lower confidence or add a concern. No confidence rating was manually upgraded.
No effect-favourability decisions	No decision was based on whether the intervention appeared beneficial or unfavourable.
Objective diagnostic trigger	Manual downgrades required a documented trigger, such as contribution profiles, prediction intervals, direct-indirect comparisons, design-level sensitivity analyses, minimal

Principle	Operational meaning
	parallelism, mean path length, or the net heat plot.
Uniform application	The same criteria were applied to all 12 comparisons versus CTRL.

Decision threshold

The primary decision threshold was ±0.20 SMD. It was defined before final CINeMA confidence adjudication and applied uniformly across comparisons. It was used as a methodological decision aid for imprecision, heterogeneity, and incoherence judgments, not as an ODI- or RMDQ-specific minimal clinically important difference. Because disability was synthesized using SMD across different instruments, no single instrument-specific MCID could be applied consistently across the network.

Domain-level and network-level criteria

Criterion	Application
Within-study bias	Contribution-weighted average RoB output was used. Manual downgrading was considered only if high-risk studies materially influenced the estimate.
Reporting bias	Reporting bias was judged as undetected/no downgrade unless comparison-specific evidence suggested missing studies, missing outcomes, selective reporting, or strong small-study effects. ROB-MEN was explored but not used as the sole automatic basis because several studies contributed valid contrast-level data without arm-level means/SDs.
Indirectness	No downgrade was applied when studies addressed the review PICO and interventions were assignable to predefined nodes. Differences in dose, supervision, or cointerventions were considered under heterogeneity/incoherence unless they represented PICO-level indirectness.
Imprecision	Automatic CINeMA judgments based on the 95% CI and the ±0.20 SMD threshold were retained unless extraction or threshold entry was incorrect.
Heterogeneity	Automatic CINeMA judgments based on the 95% PrI and ±0.20 SMD threshold were retained unless design-level sensitivity analyses, contribution profiles, or the net heat plot indicated additional instability not captured by the comparison-specific automatic judgment.
Incoherence	SIDE/direct-indirect evidence and design-by-treatment diagnostics were considered. Direct-only or indirect-only estimates retained conservative concern when coherence could not be empirically checked and the global incoherence assessment indicated concern.
Sparse-node cap	High confidence was not assigned to sparse estimates when additional objective network-level diagnostics indicated instability not fully captured by automatic comparison-specific CINeMA judgments.
Pathway/design instability	A one-level conservative downgrade could be applied when an estimate depended materially on a pathway or design flagged by sensitivity analyses or the net heat plot.

Final application to the disability NMA

Comparison	Final confidence	Downgrade domains / final adjudication criteria
STM vs CTRL	Moderate	Network-level sparse-node cap / conservative adjudication
Pilates APP vs CTRL	Moderate	Within-study bias
STMC vs CTRL	Low	Within-study bias; design-level instability
ST vs CTRL	Low	Within-study bias; design-level instability
Pilates MAT vs CTRL	Very low	Within-study bias; incoherence; design-level instability/conservative adjudication
MDT vs CTRL	Low	Imprecision; heterogeneity
SM vs CTRL	Very low	Within-study bias; heterogeneity; incoherence
STR vs CTRL	Very low	Within-study bias; heterogeneity
STRET vs CTRL	Very low	Imprecision; incoherence
GE vs CTRL	Very low	Within-study bias; heterogeneity; incoherence
UC vs CTRL	Very low	Within-study bias; imprecision; incoherence
BS vs CTRL	Very low	Imprecision; incoherence

No manual adjudication changed the effect estimates, confidence intervals, prediction intervals, P-score values, node definitions, statistical model, or eligibility criteria.

Supplementary Checklist S1

PRISMA 2020 Checklist

Section and Topic	Item #	Checklist item	Location where item is reported
**TITLE**			
Title	1	Identify the report as a systematic review.	Title page / manuscript title.
**ABSTRACT**			
Abstract	2	See the PRISMA 2020 for Abstracts checklist.	Structured abstract.
**INTRODUCTION**			
Rationale	3	Describe the rationale for the review in the context of existing knowledge.	Introduction.
Objectives	4	Provide an explicit statement of the objective(s) or question(s) the review addresses.	End of Introduction; Methods > Eligibility Criteria.
**METHODS**			
Eligibility criteria	5	Specify the inclusion and exclusion criteria for the review and how studies were grouped for the syntheses.	Methods > Eligibility Criteria; Methods > Study Design: RCTs; Methods > Classification of Interventions; Supplementary Table S2.
Information sources	6	Specify all databases, registers, websites, organisations, reference lists and other sources searched or consulted to identify studies. Specify the date when each source was last searched or consulted.	Methods > Search Strategy; Supplementary Tables S1a and S1b; PRISMA consistency summary.
Search strategy	7	Present the full search strategies for all databases, registers and websites, including any filters and limits used.	Supplementary Tables S1a and S1b.
Selection process	8	Specify the methods used to decide whether a study met the inclusion criteria of the review, including how many reviewers screened each record and each report retrieved, whether they worked independently, and if applicable, details of automation tools used in the process.	Methods > Search Strategy; Methods > Eligibility Criteria; Figure 1; Supplementary Table S3.
Data collection process	9	Specify the methods used to collect data from reports, including how many reviewers collected data from each report, whether they worked independently, any processes for obtaining or confirming data from study investigators, and if applicable, details of automation tools used in the process.	Methods > Data Extraction.
Data items	10a	List and define all outcomes for which data were sought. Specify whether all results that were compatible with each outcome domain in each study were sought (e.g. for all measures, time points, analyses), and if not, the methods used to decide which results to collect.	Methods > Eligibility Criteria, Outcomes; Methods > Data Extraction.
Data items	10b	List and define all other variables for which data were sought (e.g. participant and intervention characteristics, funding sources). Describe any assumptions made about any missing or unclear information.	Methods > Data Extraction; Supplementary Tables S4a, S5, S6 and S8.

Section and Topic	Item #	Checklist item	Location where item is reported
Study risk of bias assessment	11	Specify the methods used to assess risk of bias in the included studies, including details of the tool(s) used, how many reviewers assessed each study and whether they worked independently, and if applicable, details of automation tools used in the process.	Methods > Risk-of-Bias Assessment; Methods > Certainty of the Evidence.
Effect measures	12	Specify for each outcome the effect measure(s) (e.g. risk ratio, mean difference) used in the synthesis or presentation of results.	Methods > Data Analysis.
Synthesis methods	13a	Describe the processes used to decide which studies were eligible for each synthesis (e.g. tabulating the study intervention characteristics and comparing against the planned groups for each synthesis (item #5)).	Methods > Study Design: RCTs; Methods > Classification of Interventions; Supplementary Table S2; Supplementary Table S4b.
Synthesis methods	13b	Describe any methods required to prepare the data for presentation or synthesis, such as handling of missing summary statistics, or data conversions.	Methods > Data Extraction; Methods > Data Analysis.
Synthesis methods	13c	Describe any methods used to tabulate or visually display results of individual studies and syntheses.	Methods > Data Analysis; Table 1; Figures 3-5; Supplementary Tables S4a-S8; Supplementary Figures S1-S3.
Synthesis methods	13d	Describe any methods used to synthesize results and provide a rationale for the choice(s). If meta-analysis was performed, describe the model(s), method(s) to identify the presence and extent of statistical heterogeneity, and software package(s) used.	Methods > Data Analysis.
Synthesis methods	13e	Describe any methods used to explore possible causes of heterogeneity among study results (e.g. subgroup analysis, meta-regression).	Methods > Data Analysis; Results; Supplementary Tables S6 and S8; Supplementary Figure S2.
Synthesis methods	13f	Describe any sensitivity analyses conducted to assess robustness of the synthesized results.	Methods > Data Analysis; Results; Figure 4; Supplementary Figure S2.
Reporting bias assessment	14	Describe any methods used to assess risk of bias due to missing results in a synthesis (arising from reporting biases).	Methods > Data Analysis; Results; Supplementary Figure S3.
Certainty assessment	15	Describe any methods used to assess certainty (or confidence) in the body of evidence for an outcome.	Methods > Certainty of the Evidence; Supplementary Text S1.
**RESULTS**			
Study selection	16a	Describe the results of the search and selection process, from the number of records identified in the search to the number of studies included in the review, ideally using a flow diagram.	Results; Figure 1.
Study selection	16b	Cite studies that might appear to meet the inclusion criteria, but which were excluded, and explain why they were excluded.	Results; Supplementary Table S3.
Study characteristics	17	Cite each included study and present its characteristics.	Results > Study Characteristics; references 34-78; Supplementary Tables S4a, S4b, S5, S6 and S8.
Risk of bias in studies	18	Present assessments of risk of bias for each included study.	Results > Risk-of-Bias Assessment; Figure 2; Supplementary Figure S1.
Results of individual studies	19	For all outcomes, present, for each study: (a) summary statistics for each group (where appropriate) and (b) an effect estimate and its precision (e.g. confidence/credible interval), ideally using structured tables or plots.	Supplementary Tables S4a, S5 and S7; Table 1; Figure 5.

Section and Topic	Item #	Checklist item	Location where item is reported
Results of syntheses	20a	For each synthesis, briefly summarise the characteristics and risk of bias among contributing studies.	Results; Figure 2; Supplementary Tables S4a, S4b, S5 and S6; Supplementary Figure S1.
Results of syntheses	20b	Present results of all statistical syntheses conducted. If meta-analysis was done, present for each the summary estimate and its precision (e.g. confidence/credible interval) and measures of statistical heterogeneity. If comparing groups, describe the direction of the effect.	Results; Table 1; Figure 5; Supplementary Table S7.
Results of syntheses	20c	Present results of all investigations of possible causes of heterogeneity among study results.	Results; Figure 4; Supplementary Tables S6-S8; Supplementary Figure S2.
Results of syntheses	20d	Present results of all sensitivity analyses conducted to assess the robustness of the synthesized results.	Results; Figure 4; Supplementary Figure S2; Supplementary Table S7.
Reporting biases	21	Present assessments of risk of bias due to missing results (arising from reporting biases) for each synthesis assessed.	Results; Supplementary Figure S3.
Certainty of evidence	22	Present assessments of certainty (or confidence) in the body of evidence for each outcome assessed.	Table 1; Figure 5; Methods > Certainty of the Evidence; Supplementary Text S1.
**DISCUSSION**			
Discussion	23a	Provide a general interpretation of the results in the context of other evidence.	Discussion.
Discussion	23b	Discuss any limitations of the evidence included in the review.	Discussion > Limitations.
Discussion	23c	Discuss any limitations of the review processes used.	Discussion > Limitations.
Discussion	23d	Discuss implications of the results for practice, policy, and future research.	Discussion > Clinical Implications; Discussion > Implications for Research.
**OTHER INFORMATION**			
Registration and protocol	24a	Provide registration information for the review, including register name and registration number, or state that the review was not registered.	Methods; PROSPERO CRD42022331411.
Registration and protocol	24b	Indicate where the review protocol can be accessed, or state that a protocol was not prepared.	Methods; PROSPERO CRD42022331411.
Registration and protocol	24c	Describe and explain any amendments to information provided at registration or in the protocol.	Methods > Deviations from the Protocol; Supplementary Table S9.
Support	25	Describe sources of financial or non-financial support for the review, and the role of the funders or sponsors in the review.	Funding statement.
Competing interests	26	Declare any competing interests of review authors.	Conflicts of Interest statement.
Availability of data, code and other materials	27	Report which of the following are publicly available and where they can be found: template data collection forms; data extracted from included studies; data used for all analyses; analytic code; any other materials used in the review.	Data Availability Statement; Supplementary Materials.

From: Page MJ, McKenzie JE, Bossuyt PM, Boutron I, Hoffmann TC, Mulrow CD, et al. The PRISMA 2020 statement: an updated guideline for reporting systematic reviews. BMJ 2021;372:n71. doi: 10.1136/bmj.n71. Licensed under CC BY 4.0: https://creativecommons.org/licenses/by/4.0/
